# Chemogenomic profiling of *Plasmodium falciparum* as a tool to aid antimalarial drug discovery

**DOI:** 10.1038/srep15930

**Published:** 2015-11-06

**Authors:** Anupam Pradhan, Geoffrey H. Siwo, Naresh Singh, Brian Martens, Bharath Balu, Katrina A. Button-Simons, Asako Tan, Min Zhang, Kenneth O. Udenze, Rays H.Y. Jiang, Michael T. Ferdig, John H. Adams, Dennis E. Kyle

**Affiliations:** 1 Department of Global Health, College of Public Health, University of South Florida, Tampa, FL 33612; 2 Eck Institute for Global Health, Department of Biological Sciences, University of Notre Dame, IN 46556.

## Abstract

The spread of *Plasmodium falciparum* multidrug resistance highlights the urgency to discover new targets and chemical scaffolds. Unfortunately, lack of experimentally validated functional information about most *P. falciparum* genes remains a strategic hurdle. Chemogenomic profiling is an established tool for classification of drugs with similar mechanisms of action by comparing drug fitness profiles in a collection of mutants. Inferences of drug mechanisms of action and targets can be obtained by associations between shifts in drug fitness and specific genetic changes in the mutants. In this screen, *P. falciparum, piggyBac* single insertion mutants were profiled for altered responses to antimalarial drugs and metabolic inhibitors to create chemogenomic profiles. Drugs targeting the same pathway shared similar response profiles and multiple pairwise correlations of the chemogenomic profiles revealed novel insights into drugs’ mechanisms of action. A mutant of the artemisinin resistance candidate gene - “K13-propeller” gene (PF3D7_1343700) exhibited increased susceptibility to artemisinin drugs and identified a cluster of 7 mutants based on similar enhanced responses to the drugs tested. Our approach of chemogenomic profiling reveals artemisinin functional activity, linked by the unexpected drug-gene relationships of these mutants, to signal transduction and cell cycle regulation pathways.

The widespread use of artemisinin derivatives (ART) in combination therapies to treat *Plasmodium falciparum* led to significant reductions in deaths and disease, but resistance to this most potent class of antimalarial treatments has emerged and is spreading^1^,^2^,^3^,^4^. To understand drug resistance or mechanisms of action, find alternative drugs and identify new targets requires new experimental approaches to identify new targets and validate the mechanisms of antimalarial inhibitors on a broader phenotypic level^5^,^6^. In *Plasmodium*, whole-cell chemical-genetic methods that are scalable to identify high value, druggable genes from critical pathways of the parasite life cycle are still nascent^7^,^8^. Traditionally, genes associated with active compounds are identified using drug resistant strains and field isolates; however, these methods are limited in their sensitivity and can yield population-specific conclusions^9^. Other studies used DNA microarray and sequencing to analyze the function of inhibitors (e.g., spiroindolones) at an organelle or whole genome level to identify potential targets^10^,^11^,^12^. Additionally, massive chemical screens have identified novel lead compounds for which targets and mechanisms of action are generally unknown^13^,^14^. The addition of a chemogenomic approach that functionally profiles *P. falciparum piggyBac* mutants can potentially speed up the antimalarial drug discovery process similar to other programs (e.g., NCI60). Functional profiling creates a chemogenomic profile of drug fitness changes in which drugs with similar mechanisms of action lead to similar fitness profiles of various mutants. Comparison of chemogenomic profiles to each drug across a set of mutants can help classify lead compounds with unknown mechanisms of action relative to well-characterized drugs with established mechanisms of action.

The National Cancer Institute operated the NCI60 program for years and screened new compounds for efficacy (IC_50_, IC_90_) against the same collection of 60 different cancer cell lines^15^. Pairwise associations between the response profiles of known and unknown inhibitors determined if the mechanism of action of the new compound was unique. Chemogenomics uses a similar approach to functionally link hypothetical or unknown genes to specific biochemical and metabolic processes by pair-wise associations of drug responses between mutants with known genetic mutations.

Forward genetic approaches in model organisms and some microbial pathogens have provided robust empirical detection of genetic factors associated with phenotypic traits, including drug resistance and mechanisms of action^9^,^16^,^17^,^18^,^19^,^20^. Typically, forward genetics requires an initial mutagenesis step to create diversity in an otherwise uniform genetic background. Random *piggyBac* insertional mutagenesis of *P. falciparum* provides such an unbiased method to create a collection of unique isogenic mutant clones for phenotypic screens^21^,^22^. In this work we establish a chemogenomic profiling method using single insertion *piggyBac* mutant clones of *P. falciparum* to connect molecular mechanism of drug action to gene functions and their metabolic pathways, this includes linking unknown or hypothetical genes to metabolic pathways based on their shared response relationships^23^ (Fig. 1a).

The *piggyBac* mutants used in this chemogenomic screen carry a single genetic lesion in a uniform genetic background (NF54) validated by sequence analysis^21^. The 71 mutant clones of *P. falciparum* formed a *piggyBac* library of disruptions in genes of diverse Gene Ontology (GO) functional categories^21^ (Fig. 1b and Table S1). Each insertion creates a unique phenotypic footprint of the distinct gene-associated processes, which can be mapped to other defined parameters like molecular structure of the drugs, affected metabolic processes, and molecular targets. Changes in expression level, alteration of the temporal pattern of expression, and simple knockouts can all provide suitable changes in metabolic function to reveal a signature that links a phenotype to a specific process or pathway by the associated genetic mutation.

As a proof of concept, we investigated the growth inhibitory effect of the cyclophilin inhibitor cyclosporine A (CsA) on parasites in which loci of two hypothetical RNA binding proteins (PF3D7_1360100 and PF3D7_0812500) were disrupted by *piggyBac* insertions; these genes were previously proposed to interact with a group of six cyclophilins and two peptidyl-prolyl-cis-trans isomerases^24^. Disruption of these two genes resulted in >20 fold resistance to CsA, but had no altered growth effect when exposed to FK-506, an inhibitor of calcineurin through inhibition of the FK-506 binding protein (Fig. 1c)^25^. Both CsA and FK-506 inhibit calcineurin: CsA through binding to cyclophilin and FK-506 through an interaction with FKBP^26^. The observed >20 fold IC50 shifts after treatment of these cyclophilin-related *piggyBac* mutants with CsA but not FK-506 is consistent with the expectation that CsA interferes with cyclophilin activity while FK-506 does not, in spite of the ability of both drugs to inhibit calcineurin. Hence, these observations validate our approach in predicting MOA, even for two highly related compounds that affect the same pathway but through distinct molecular processes. These results indicate that chemogenomic profiling of the *piggyBac* mutant library could also reveal unexpected drug relationships and connect them to gene functions, including hypothetical genes in the malaria parasite. Consequently, we profiled 71 *piggyBac* mutants for altered responses to standard antimalarial drugs and inhibitors of known metabolic pathways (Fig. 1d and Table S2). Phenotypes for the chemogenomic profiles were determined from quantitative dose response at the half maximal inhibitory concentration (IC_50_s) of the parental clone NF54 and each of the mutants to a library of antimalarial drugs and inhibitors of metabolic pathways (Fig. 1e).

Pairwise genotype-phenotype associations based on IC_50_ growth responses of *piggyBac* mutants to a wide range of inhibitors (Table S3) normalized to that of wild type NF54 parasites (Table S4) allowed assessment of genotype-phenotype associations among inhibitors and *piggyBac* mutants. These chemogenomic profiles were visualized by two-dimensional hierarchical clustering to discern chemogenomic interactions (Fig. 2a). Genes with similar chemogenomic signatures were clustered horizontally and the compounds associated with the inhibitors displaying similar phenotypic patterns were clustered on the vertical axis. High-grade resistance to dihydrofolate reductase (DHFR) inhibitors served as a positive control since the human DHFR (hDHFR) was used as the selectable marker in the *piggyBac* insert for most mutants used in this analysis.

We then evaluated complex relationships between drug pairs by constructing drug-drug networks in which nodes are drugs and edges (lines connecting nodes) represent the strength of the Spearman correlation between drug pairs across all mutants to define distinct ‘drug sensitivity’ clusters (Fig. 2b). As a confirmation of the chemogenomic profiles to predict drug mechanism of action, we found that drugs targeting the same pathway were more similar to each other than to drugs targeting other pathways (correlation between chemogenomic profiles, *r* = 0.33 for drugs in the same pathway versus *r* = 0.24 for drugs not in the same pathway; Wilcoxon rank sum test, *P* = 0.01) (Table S5). In all but one of the pathways considered, at least one drug pair with the same mechanism of action is predicted to interact in the drug-drug network (Fig. 2b). Binary associations of classified inhibitors and the known genetic defects of the *P. falciparum* mutants generated chemical-genomic signature profiles reflecting antimalarial drug mechanism of action. The chemogenomic profiles consistently identified antimalarial drug pairs known to have similar activity to each other, an observation that is consistent with other studies using whole cell parasite isolates and chemotypes^9^,^13^,^14^ (Table S5).

An important caveat is that inaccurate annotations of expected drug effects could lead to misinterpretation due to an apparent absence of a reported effect on the pathway. Such lack of information would compromise our ability to differentiate between novel interactions among drugs predicted by the chemogenomic profiles versus false positive correlations (interactions). Conversely, similarity in chemogenomic responses between drugs with unknown mechanisms of action and those with well-characterized targets might lead to identification of unexpected interactions of drugs with targets. For example, we observed that responses to the iron-sulphur cluster inhibitor rotenone and the bc_1_ complex inhibitor atovaquone are positively associated with lumefantrine (Fig. S1), a drug that is used with artemether and is not known to target the mitochondrion^27^. This surprising association between atovaquone and lumefantrine requires further study to ensure it is not an anomaly, yet the result indicates the potential for deciphering novel drug mechanisms of action.

Network analysis can robustly capture these and other important drug-gene interactions related to each drug sensitivity cluster, by using the same data used to define drug:drug relationships. For example, inhibitors acting on related parasite biosynthetic pathways grouped together based on their drug response profiles, which is similar to grouping of compounds targeting the same organelles (*e.g.* inhibitors of hemoglobin digestion; Fig. 2b, red nodes). Therefore, drug-drug (Fig. 2b) and gene-gene networks (Fig. 2c) deduced from the chemogenomic profiles provide new evidence to aid in understanding drugs’ mechanisms of action as well as identify potential drug targets and resistance genes. This approach is complimentary to hierarchical clustering; however, it identifies more complex relationships and is arguably better at defining non-arbitrary clusters. Clear evidence of the predictive ability of our chemogenomic profiling approach is provided in the identification of an ART sensitivity cluster of mutants that includes a mutant of the gene encoding K13-propeller (PF3D7_1343700) linked to artemisinin resistance^1^,^2^,^28^ (Fig. 3a). In this mutant of K13, the transposon is located within the putative promoter region and alters the normal expression pattern. Instead of a maximal time of expression in early ring stage, as in the parent line of NF54, qRT-PCR analysis of the mutant parasite revealed K13 transcription is highly upregulated thereafter in the intraerythrocytic development cycle in an expression pattern likely driven by the promoter of the drug selection cassette (Fig. S2). Bioinformatics analysis of the ART sensitivity cluster was then used to extend our new understanding of the biological basis for ART mechanism of action. The network analysis of gene-gene interactions was integrated with an independent gene co-expression network constructed from a diverse set of transcriptional data^29^. GO enrichment analysis of direct neighbors of K13-propeller in the co-expression network and other genes in the ART sensitivity cluster linked DNA metabolic processes, cellular stress responses, and lipid biosynthesis with gene targets associated with ART mechanism of action (Fig. 3b). The genes that are highlighted in the ART sensitivity cluster and the 159 genes directly connected to K13-propeller in the co-expression network are not implicated directly in any of the recent papers relating to ART resistance. However, further analysis of all SNPs in the recent GWAS^30^ identified eight genes (Table S9) in the independent co-expression network that contain SNPs that are associated with parasite clearance half-life (FDR 0.05). The genes in the ART sensitivity cluster did not contain any SNPs associated with delayed parasite clearance half-life.

Further analysis of the 7 mutants contained in the ART sensitivity cluster (Fig. 3a) identified several drugs that were highly correlated with ART (Fig. 3c). Known targets of these drugs are consistent with pathways that have been tied to proposed ART mechanism of action or resistance (DNA repair, fatty acid synthesis, calcium ion metabolism and hemoglobin metabolism)^31^ and potential new pathways identified in our analysis (*e.g.*, calcineurin signaling, cell cycle, autophagy). Drugs with the highest correlations to ART compounds also target pathways of genes directly connected to K13-propeller in the co-expression network analysis (Fig. 3b) and represent pathways relevant to ART resistance mechanisms. Importantly, excluding the K13 mutant and repeating the hierarchical clustering and bootstrap analysis still produces a highly significant cluster (*p* < 0.05) containing the other mutants in the ART sensitivity cluster from the original analysis (data not shown). Identification of these genes provides new candidates for monitoring the spreading resistance to ART, which is especially important as evidence emerges of alternative resistance mechanisms not linked to known mutations in the K13-propeller^2^,^28^.

This study presents the first chemogenomic screen of *P. falciparum* using a random *piggyBac* insertional mutant library. Chemogenomic profiles from *P. falciparum* mutants provided unique signatures that mapped molecular structures of drugs to their targets demonstrating this technique can be an important tool to annotate mechanism of action of drugs and for validating novelty of compounds to vulnerable pathways of malaria parasites. The necessary addition of many more mutants and drugs will more precisely define the chemical-genomic spaces of the cell-drug interface, which will significantly augment the sensitivity and predictive capacity to define mechanisms of action, functionally annotate hypothetical genes, and identify interactions among metabolic pathways. Thus this approach provides a robust addition to the set of experimental tools for antimalarial drug research to improve target identification and understanding of mechanism of action.

## Materials and Methods

### Selection of pathway specific chemical inhibitors

Growth inhibitors belonging to diverse chemical scaffolds were identified to cover all critical biochemical pathways in *Plasmodium*. These inhibitors are known to kill *Plasmodium* by its association with a specific gene product, were tested for their growth inhibition in the wild type *P. falciparum* NF-54 strain in a 384 well plate format at 12 fold 1:3 serial dilutions (Table S1). After initial calibration assays only 53 inhibitors (which also include common antimalarials with unknown targets) were filtered out and added to the library for chemical-genetics analysis. All inhibitors were purchased from Sigma-Aldrich (St. Louis, MO) unless otherwise mentioned except for Erythro and Threo-PPMP from Matreya, LLC (Pleasant Gap, PA), cycloguanil and atovaquone was a gift from WRAIR, USA.

### Parasite strains, piggyBac mutant library and culture maintenance

The wild type *P. falciparum* clone NF54 and all *piggyBac* single loci mutants were generated from previously reported transfections using pXL-BACII-HDFR/BSD and pHTH^22^. All strains and *piggyBac* parasites were cultured in 4% hematocrit (A+ erythrocytes from Interstate blood bank, Memphis, TN) and 1% Albumex II in RPMI 1640 medium (Invitrogen) supplemented with 50 μg/ml hypoxanthine (Sigma, St. Louis, MO) and 25 mM HEPES (Invitrogen) which is in accordance to the standard method described by Trager and Jensen, 1976^32^. The flasks containing cultures were grown in a dedicated incubator with continuous flow of mixed gas (90% Nitrogen, 5% CO_2_ and 5% O_2_ respectively).

### High-Throughput piggyBac phenotype growth assays

We developed an automated platform for mutant *P. falciparum* functional genetics study using a high-throughput forward genetic screen. A library of 53 inhibitors, at least 2 per pathway targeting at different levels *Plasmodium* metabolism were assayed. In all chemical-genetic screens, compound dilutions and mutant culture dispensing to assay plates was handled robotically with a Beckman-Coulter liquid handling system (BIOMEK 3000 series, Beckman Coulter). All assays were conducted in a 384 well culture plate with a total assay volume of 50 μl where compound stock was added in 10× to the final assay volume.

In each assay, highly synchronous (5% sorbitol treated) ring-stage culture with >80% confluence were considered for growth assays. Briefly, the parasitemia and hematocrit was 0.5 and 1.5% respectively and the cultures were grown in a humidified mixed gas saucer for 72 hours. The growth response for each inhibitor dilution was obtained by reading the fluorescence generated by DNA intercalating dye SYBRGreen I^33^. Each batch of *piggyBac* mutant assays were accompanied with wild type NF54. The growth inhibition concentration affecting 50% and 90% (GI_50_ and GI_90_) parasite growth was calculated by plotting the relative fluorescence unit values (RFU) of the SYBRGreen I added to culture plates. Statistically, each data point was converted to fit a non-linear logistic dose response function (DataAspects Plate Manager, DataAspects Corporation, California, USA). The assay values Mean±SD of least three assays, R^2^ (coefficient of determination) >0.9 was considered significant and considered for chemogenomic correlations.

### Inhibitor validation and phenotype signatures

To generate profiles of the *piggyBac* mutants and to derive chemotype-phenotype associations, three parameters were considered out of chemogenomic assays for each inhibitor: 1) GI_50_, 50% growth inhibitory concentration; 2) Relative Phenotype Response (RPR): [GI_50*pB*_ over GI_50WT_ (GI_50*pB*_/GI_50WT_)]. RPRs are described either as a shift towards resistance (i.e., +ve increment from the wild type GI_50_) or sensitivity (decreased GI_50_ value from the wild type). The RPR for each mutant is scored from the wild type assayed along with the mutants except for *piggyBac* PF3D7_0812500 and PF3D7_1360100.

### Chemotype-genotype association through phenotypic clustering

A genotype-phenotype association was made by cluster analysis of RPRs. Clustering was executed using a standard agglomerative algorithm described by Eisen *et al.*, 1998^34^. Genes were clustered by average linkage to calculate minimum distance using a scaled, uncentered Spearman correlation matrix. All data were transformed to logarithmic base 2 and hierarchical clustering results were visualized in R using the heatmap.2 function in the gplots package. To assess statistical significance of clusters, the R package pvclust was used to calculate *p* for each branch point in the *piggyBac* and drug dendrograms, the package was modified to allow a Spearman correlation based distance metric. The output is displayed graphically, conveying the clustering and the essential similarity in RPR of the gene functions in presence of an inhibitor. Thus patterns like increased resistance is positive shift in GI_50_ and vice-versa for sensitivity. A distinct subset of Gene Ontology (GO) functional annotations relevant to the pathways indicated in the study was used to annotate each gene in the chemogenomic interaction dataset for color coding in cluster analysis. Any gene not falling into the defined category has been designated as ‘other’. The genes falling under multiple annotations, we choose to define it as a most probable on the basis of reviews and similarities published concerning the gene. Information on all genes can be found in the *Plasmodium* genome resource like (http://plasmodb.org/plasmo/), GeneDB (http://www.genedb.org/Homepage/Pfalciparum) and KEGG GENES Database (http://www.genome.jp/kegg/genes.html).

### Construction of drug-drug, gene-gene and drug-gene networks

Correlation between chemogenomic profiles of various drugs was determined using the spearman correlation coefficient. For each drug pair, a permutation test was conducted, in which a random distribution of 1000 correlation coefficients was obtained by permuting the chemogenomic profile of a given drug pair 1000 times, followed by computation of a correlation coefficient in each instance of permutation. Correlation between any drug pair was regarded as significant if the observed correlation coefficient was greater than any of the 1000 correlation coefficients observed in the permutations of the drug pair’s profiles. Drug pairs whose correlation met this criterion were regarded as interacting. Visualization of the drug-drug network interactions was performed in cytoscape^35^. Similarly, a piggyBac gene-gene network was constructed using the spearman correlation co-efficient of each pair of piggyBac mutants across all drugs. For each drug pair, a permutation test was conducted and a correlation between any drug pair was regarded as significant if was greater than any of the 1000 correlation coefficients observed in the permutations of the drug pair’s profiles. Visualization of the gene-gene network interactions and network clustering was performed in cytoscape^35^ using the MCODE algorithm, which identifies clusters of highly interconnected nodes^36^. Drug-gene interactions were considered as significant when a specific mutant exhibited at least 3 fold resistance/ sensitivity to a given drug.

### qRT-PCR method

A comparative CT method was used and for every plate a standard curve was set with 5 dilutions for target gene and reference gene. A comparison of all the time point samples with NF54 Time point 1 (2 hour) was made against a pooled reference comprised of NF54 RNA samples from 5 time points. Genes used in previous studies were used as endogenous control genes as reference, *seryl-tRNA synthetase* (PF07–0073) and *actin* (PFL2215w)^37^, and the qRT-PCR was completed using Agilent Mx3000P qPCR System in reactions of 20 μl volumes using RT2 SYBR Green qPCR Mastermixes (Qiagen). The cycling conditions were 95 C for 15 min followed by 40 cycles of 94 C for 30 s, 54 C for 40 s and 68 C for 50 s with a final extension at 68 C for 10 min.

### Data Availability

The data reported in this paper are tabulated in the supplemental materials and mutant parasites are deposited with MR4 - BEI Resources (http://www.beiresources.org/About/MR4.aspx).

## Additional Information

**How to cite this article**: Pradhan, A. *et al.* Chemogenomic profiling of *Plasmodium falciparum* as a tool to aid antimalarial drug discovery. *Sci. Rep.* **5**, 15930; doi: 10.1038/srep15930 (2015).

## Supplementary Material

Supplementary Information

## Figures and Tables

**Figure 1:**
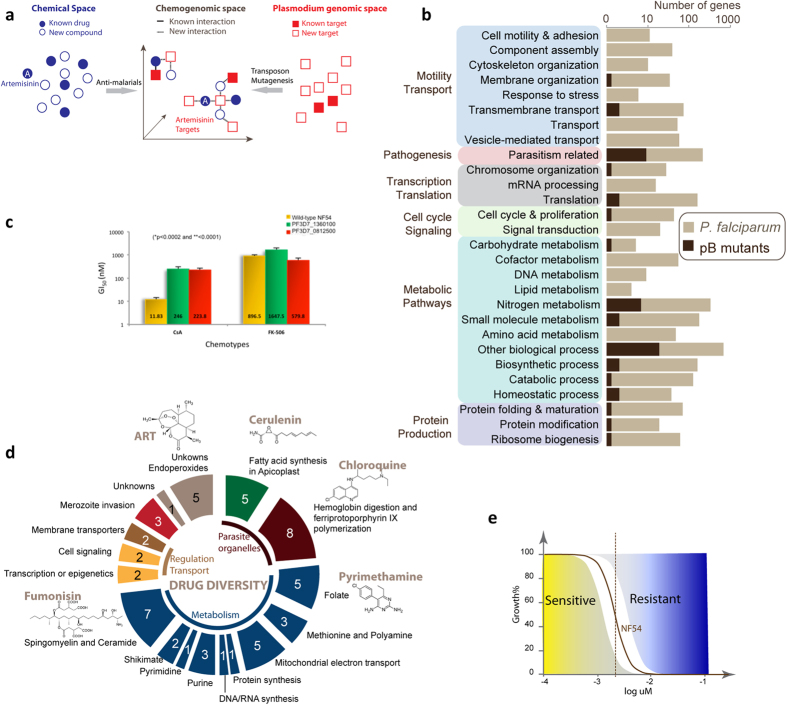
Chemogenomic profiling of *piggyBac* mutant clones of *P. falciparum*. (**a**) Hypothetical chemical-genomic interactions (redrawn from^6^). Similar mechanisms of action of unknown compounds can be defined in chemogenomic analysis by pair-wise comparison of responses with known drugs across mutants. (**b**) Overview of *piggyBac* insertions in the *P. falciparum* genome. The *piggyBac* mutants used in this study included insertions in genes of diverse GO categories and many essential biochemical pathways. The dark brown bars represent the proportion of genes of *piggyBac* insertions in each GO category (Generic GoSlim) with respect to the entire proteome of *P. falciparum*. (**c**) Growth inhibitory effect of CsA, an inhibitor of cyclophilin and FK-506, an inhibitor of FKBP on *piggyBac* mutants in which genes encoding two hypothetical RNA binding proteins that were predicted to interact with 6 different cyclophilins have been disrupted^24^. The resistance manifested in presence of CsA and FK-506 was statistically calculated significant (* = P < 0.0002 and ** < 0.0001). (**d**) Diversity of inhibitors and antimalarial drugs used in screen. Representative drugs are shown for some categories. The mechanism of actions have been derived from science literature search and compilation, as listed in Supplemental Table S2 (the drug mechanism table). (**e**) hypothetical dose response data for *piggyBac* mutants with varying degrees of susceptibility. A dashed line crosses the hypothetical drug response curve at the IC_50_ indicates the 50% growth inhibitory concentration of drug in an assay. The clear area along the drug response curve indicates variations that do not reflect a significant change from the dose response of NF54. A shift to the right would reflect an increased drug concentration necessary to achieve the same effective inhibitory concentration as NF54, or increased resistance for a *piggyBac* mutant. A shift to the left would reflect a decreased drug concentration necessary to achieve the same effective inhibitory concentration as NF54, or increased sensitivity for a *piggyBac* mutant. The blue-yellow color scheme is used in Figure 2a to reflect relative to NF54 *piggyBac* mutant changes in resistance and sensitivity, respectively.

**Figure 2:**
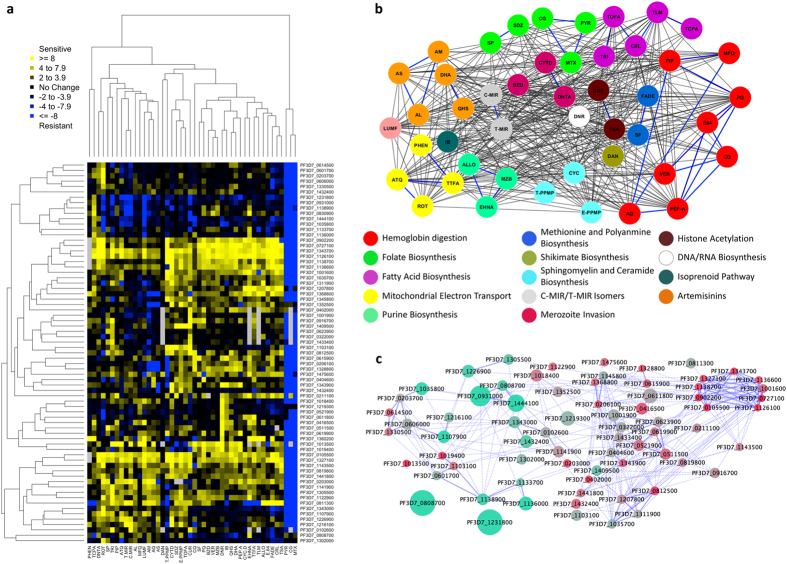
Chemogenomic signatures of *P. falciparum piggyBac* mutants. (**a**) Chemogenomic signatures of *P. falciparum piggyBac* mutants organized according to similarity in phenotypic profiles by 2-dimensional hierarchical clustering. Chemogenomic signatures for each *piggyBac* mutant consist of the RPR [GI_50*pB*_ over GI_50WT_ (GI_50*pB*_/GI_50WT_), where the GI_50_ is based on growth curves, R^2^ of >0.9] for a diverse collection of target-specific inhibitors (Table S8). The intensity of the blue color is proportional to the resistance of a mutant to an inhibitor and intensity of yellow indicates sensitivity. All data were log_2_ transformed and relative phenotypic ratios (RPR) were used to construct correlations. (**b**) A drug-drug network based on chemogenomic profiling of the *piggyBac* mutants contained 47 nodes and 415 edges representing about 19% of the maximum possible pairwise interactions attainable. A drug pair was considered as interacting (blue lines) if its observed correlation coefficient was greater or equal to that observed in 1000 permutations of the same drug pair (Permutation test, P < 0.001). Edges between drug pairs acting in the same pathway demonstrate drug:drug relationships within the chemogenomic profiles. Color coding identifies common GO categories of biological pathways. (**c**) A *piggyBac* *gene:gene* interaction network created from chemogenomic response profiles of 71 *piggyBac* mutants (see also Table S7). The edges represent *piggyBac* mutants with highly correlated chemogenomic response profiles, where the correlation coefficients were greater than or equal to that observed in 1000 permutations (Permutation test, P < 0.001) of the chemogenomic profiles for each node pair). Solid edges indicate a cluster of highly interconnected nodes (as identified by MCODE in cytoscape^36^) and dashed edges indicate non-cluster edges. The largest cluster has increased ART susceptibility, referred to as the K13 Kelch cluster with the addition of PF3D7_1001600 and PF3D7_1327100. Node size and color corresponds to DHA and QHS susceptibility, respectively, for each *piggyBac* mutant. Tightly interconnected regions of the network identified sets of genes with similar function within this gene-gene network^38^,^39^.

**Figure 3:**
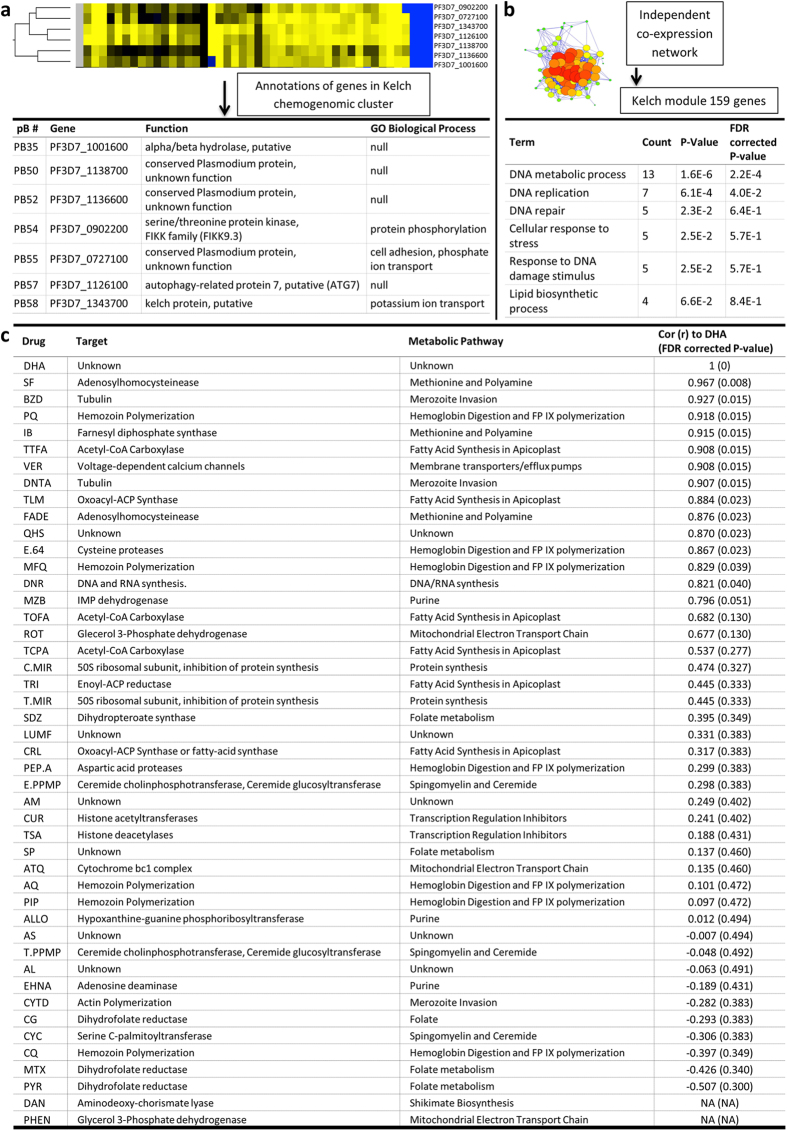
ART sensitivity cluster. (**a**) Annotations of genes affected by *piggyBac* insertions in each of the *piggyBac* mutants in the Kelch sensitivity cluster. (**b**) Initial chemogenomic profiling of 71 *piggyBac* mutants identified a cluster of 7 ART sensitive mutants, including the K13 Kelch mutant (*piggyBac* mutant 58). A. GO enrichment analysis of direct neighbors of K13 propeller from a gene-gene coexpression network identified pathways linked to K13 ascertain gene function. (**c**) Drugs and inhibitors showing a significant correlation with DHA. Metabolic pathways targeted by these compounds may reflect shared mechanisms of action with DHA and other artemisinin compounds.
