# Supplementary material for: Chemogenomic profiling of Plasmodium falciparum as a tool to aid antimalarial drug discovery
Source: Sci Rep. 2015 Nov 6;5:15930. doi: 10.1038/srep15930 (PMC4635350; doi:10.1038/srep15930)
Supplement: Supplementary Information [file srep15930-s1.pdf]

Supplemental information for:

Title: Chemogenomic profiling of *Plasmodium falciparum* as a tool to aid antimalarial drug discovery

Authors: Anupam Pradhan[1], Geoffrey Siwo[2†], Naresh Singh[1], Brian Martens[1] Bharath Balu[1*], Katrina Button-Simons[2], Asako Tan[2], Min Zhang[1], Kenneth O. Udenze[1], Rays H.Y. Jiang[1], Michael T. Ferdig[2], John H. Adams[1*] & Dennis E. Kyle[1*]

[1]Department of Global Health, College of Public Health, University of South Florida, Tampa, FL 33612.

[2]Eck Institute for Global Health, Department of Biological Sciences, University of Notre Dame, IN 46556

[*]Corresponding authors: dkyle@health.usf.edu or jadams3@health.usf.edu.

Figure S1.

.

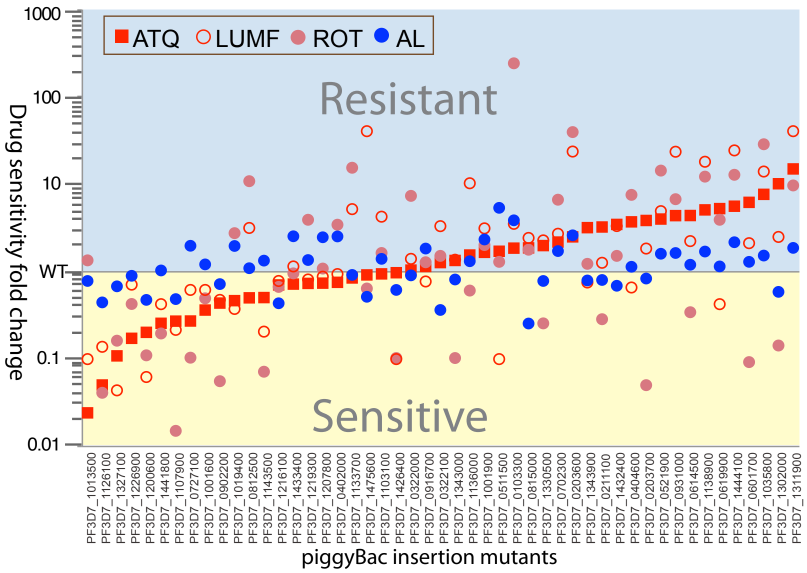

Figure S1: **Inhibitors with diverse scaffolds targeting at different levels of the mitochondrial electron transport chain.** A rank scatter plot of IC50 values of pB mutants towards LUMF, ATQ, and ROT. The drug response of LUMF appears to be close to ATV and ROTE rather than that of ARTL, which is more or less a straight line pattern. A correlation coefficient of 1000 permutations between the drug pair ATV-LUM was observed 0.64, which is greater that observed with AL-LUMF which is 0.36 (p-value <0.001).

Figure S2

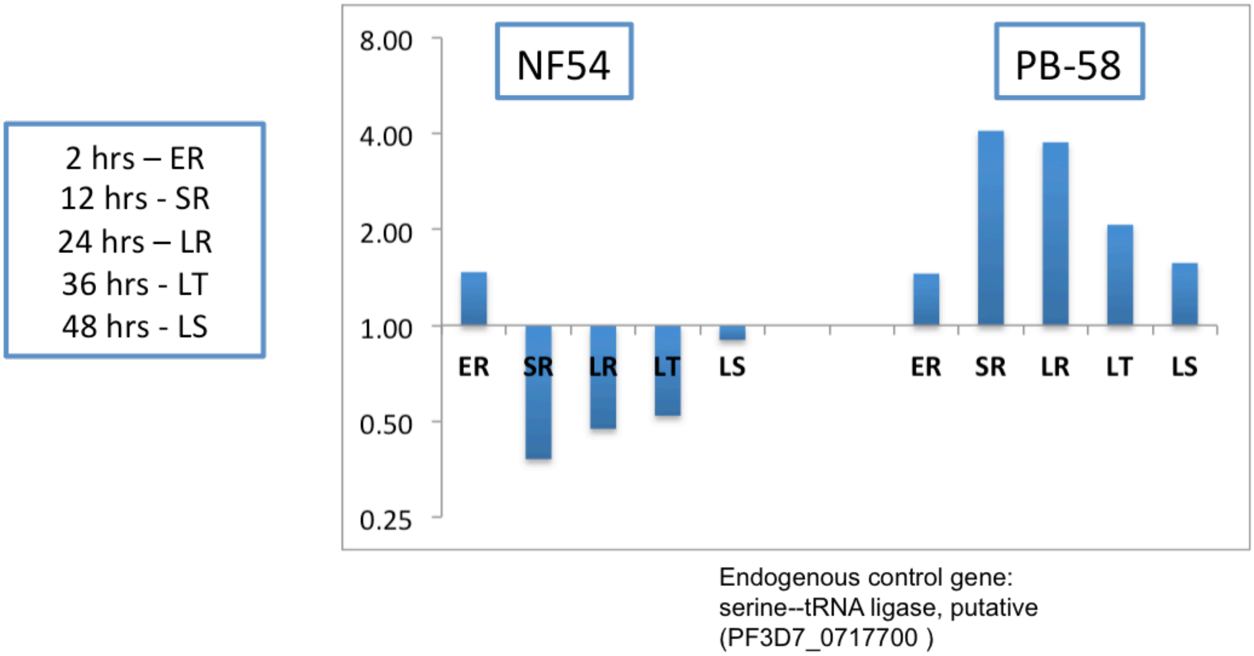

Figure S2: **qRT-PCR.** Quantitative analysis of the expression profile of the K13 propeller gene in WT NF54 and PB58, which carries a *piggyBac* insertion in the putative promoter region of the gene. The profiles are normalized against the constitutive-expressed gene PF3D7_071770. Although the K13 mutant does have slower growth than NF54, overall growth did not correlate with IC50 for most drugs (Pearson's r <0.1).

Table S1. List of P. falciparum piggyBac mutant clones

PB clone#	geneID	old ID	annotation	GO functional pathway
1	PF3D7_0811300	MAL8P1.104	CAF1 family ribonuclease	Membrane/Structural proteins, trafficking and signal transduction
2	PF3D7_1305500	PF13_0027	Protein phosphatase, putative	Biochemical pathways/protein metabolism/protein modification
3	PF3D7_0615900	PFF0770c	Conserved Plasmodium protein	Biochemical pathways/protein metabolism/protein modification
4	PF3D7_1122900	PF11_0240	Dynein heavy chain	Membrane/Structural proteins, trafficking and signal transduction
5	PF3D7_1141900	PF11_0431	inner membrane complex protein 1b, putative	Membrane/Structural proteins, trafficking and signal transduction
6	PF3D7_1475600	PFF0675c	Bromodomain protein, putative	Protein binding
7	PF3D7_0211100	PFB0495w	Conserved Plasmodium protein	Membrane/Structural proteins, trafficking and signal transduction
8	PF3D7_0203000	PFB0145c	Kid domain containg protein	Biochemical pathways/protein metabolism/protein modification
9	PF3D7_0404600	PFD0225w	Conserved Plasmodium membrane protein	Conserved proteins with unknown function
11	PF3D7_0416500	PF10_0178	Conserved Plasmodium protein	Conserved proteins with unknown function
12	PF3D7_1018400	PFD0200c	conserved Plasmodium protein	Conserved proteins with unknown function
13	PF3D7_1360200	MAL13P1.299	Conserved protein	Conserved proteins with unknown function
14	PF3D7_0511500	PFE0570w	RNA pseudouridylate	Biochemical pathways/protein metabolism/protein modification
15	PF3D7_0611800	PFF0575c	Conserved Plasmodium protein	Conserved proteins with unknown function
16	PF3D7_0619900	PFF0970w	Splicing factor 3a subunit	Nucleic acid metabolism/Nucleic acid binding/Transcription
17	PF3D7_0521900	MAL5P1.219	conserved Plasmodium protein, unknown function	conserved Plasmodium protein, unknown function
18	PF3D7_1219300	PFL0935c	Erythrocyte membrane protein 1, PfEMP1	Exported proteins
19	PF3D7_1133700	PF11_0347	Conserved Plasmodium protein	Nucleic acid metabolism/Nucleic acid binding/Transcription
20	PF3D7_0808700	PF08_0106	Erythrocyte membrane protein 1, PfEMP1	Exported proteins
21	PF3D7_1136000	PF11_0371	Conserved Plasmodium protein	Conserved proteins with unknown function
22	PF3D7_0931000	PFI1505c	Elongation factor Tu family protein-related	Biochemical pathways/protein metabolism/protein modification
23	PF3D7_0830900	PF08_0001	Plasmodium exported protein	Conserved proteins with unknown function
24	PF3D7_1231800	PFL1530w	Asparagine-rich protein	Conserved proteins with unknown function
25	PF3D7_1035800	PF10_0350	Probable protein	Conserved proteins with unknown function
26	PF3D7_1444100	PF14_0419	Conserved Plasmodium protein	Biochemical pathways/protein metabolism/protein modification
28	PF3D7_1138900	PF11TR005	Non Protein Coding, unspecified product	Non Protein Coding, unspecified product
29	PF3D7_1441800	PF14_0397	SNF7 family protein	Membrane/Structural proteins, trafficking and signal transduction
30	PF3D7_1345800	PF13_0243	Conserved Plasmodium protein	Conserved proteins with unknown function
31	PF3D7_1432400	PF14_0305	Leucine-rich repeat protein 5	alpha-2 macroglobulin receptor-associated protein activity
32	PF3D7_0819800	MAL8P1.60	conserved Plasmodium protein	Conserved proteins with unknown function
33	PF3D7_1207800	PFL0390c	Conserved Plasmodium protein	Conserved proteins with unknown function
34	PF3D7_1143500	PF11_0448	Conserved Plasmodium protein	Conserved proteins with unknown function
35	PF3D7_1001600	PF10_0020	Alpha/beta hydrolase	Biochemical pathways/protein metabolism/protein modification
36	PF3D7_1019400	PF10_0187	60S ribosomal protein L30e	Biochemical pathways/protein metabolism/protein modification
37	PF3D7_1311900	PF13_0065	Vacuolar ATP synthase, catalytic subunit a	Biochemical pathways/protein metabolism/protein modification
38	PF3D7_1035700	PF10_0348	Erythrocyte membrane protein	Exported proteins
39	PF3D7_1103100	PF11_0043	60S ribosomal protein P1	Biochemical pathways/protein metabolism/protein modification
42	PF3D7_1216100	PFL0775w	Conserved Plasmodium protein	Conserved proteins with unknown function
43	PF3D7_0206100	PFB0270w	Iron-sulfur assembly protein	Biochemical pathways/protein metabolism/protein modification
44	PF3D7_1352500	PF13_0272	Thioredoxin-related protein	Biochemical pathways/protein metabolism/protein modification
45	PF3D7_1226900	PFL1300c	Conserved Plasmodium protein	Conserved proteins with unknown function
46	PF3D7_1368800	MAL13P1.346	DNA repair endonuclease	Nucleic acid metabolism/Nucleic acid binding/Transcription
47	PF3D7_1432400	PF14_0305	Leucine-rich repeat protein 5	nuclear mRNA export factor, TAP homolog, putative
48	PF3D7_1343900	MAL13P1.385	RNA binding protein	Nucleic acid metabolism/Nucleic acid binding/Transcription
49	PF3D7_1328800	PF13_0152	transcriptional regulatory protein sir2a (SIR2A)	Nucleic acid metabolism/Nucleic acid binding/Transcription

Table S1. List of P. falciparum piggyBac mutant clones

50	PF3D7_1138700	PF11_0398	Conserved Plasmodium protein	Conserved proteins with unknown function
51	PF3D7_1327100	MAL13P1.154	Conserved Plasmodium protein	Conserved proteins with unknown function
52	PF3D7_1136600	PF11_0378	Conserved Plasmodium protein	Conserved proteins with unknown function
54	PF3D7_0902200	PFI0105c	Serine/Threonine protein kinase, FIKK family	Biochemical pathways/protein metabolism/protein modification
55	PF3D7_0727100	MAL7P1.149	Conserved Plasmodium protein	Conserved proteins with unknown function
56	PF3D7_0105500	PFA0270c	Conserved Plasmodium protein	Conserved proteins with unknown function
57	PF3D7_1126100	PF11_0271	ThiF family protein	Biochemical pathways/protein metabolism/protein modification
58	PF3D7_1343700	PF13_0238	Kelch protein	Membrane/Structural proteins, trafficking and signal transduction
60	PF3D7_1330500	PF13_0164	Conserved Plasmodium protein	Conserved proteins with unknown function
61	PF3D7_1343000	MAL13P1.214	Phosphoethanolamine N-methyltransferase	Biochemical pathways/protein metabolism/protein modification
62	PF3D7_1302000	PF13_0076	Plasmodium exported protein	Conserved proteins with unknown function
64	PF3D7_1107900	PF11_0092	Mechanosensitive ion channel protein	Membrane/Structural proteins, trafficking and signal transduction
65	PF3D7_0606000	PFF0295c	Conserved Plasmodium protein	Conserved proteins with unknown function
66	PF3D7_0614500	PFF0700c	60S ribosomal protein L19	Biochemical pathways/protein metabolism/protein modification
67	PF3D7_0203700	PFB0175c	Nucleolar preribosomal assembly protein	Biochemical pathways/protein metabolism/protein modification
68	PF3D7_0601700	PFF0085w	Plasmodium exported protein (PHISTa), pseudogene	Exported proteins
71	PF3D7_1001900	PF10_0023	Plasmodium exported protein (hyp16)	Exported proteins
72	PF3D7_1409500	PF14_0093	Conserved Plasmodium protein	Conserved proteins with unknown function
73	PF3D7_0916700	PFI0820c	RNA binding protein	Nucleic acid metabolism/Nucleic acid binding/Transcription
74	PF3D7_0402000	PFD0090c	Plasmodium exported protein (PHISTa)	Exported proteins
75	PF3D7_0102600	PFA0130c	Serine/Threonine protein kinase, FIKK family	Biochemical pathways/protein metabolism/protein modification
76	PF3D7_1433400	PF14_0315	Conserved Plasmodium membrane protein	Biochemical pathways/protein metabolism/protein modification
77	PF3D7_0322000	PFC0975c	Peptidyl-prolyl cis-trans isomerase	Biochemical pathways/protein metabolism/protein modification
78	PF3D7_0623900	PFF1150w	Ribonuclease HII	Nucleic acid metabolism/Nucleic acid binding/Transcription
80	PF3D7_1013500	PF10_0132	Phosphoinositide specific phospholipase C	Membrane/Structural proteins, trafficking and signal transduction
87	PF3D7_0812500	PF08_0086	RNA binding protein	Biochemical pathways/protein metabolism/protein modification

Table S2: Inhibitors and target Metabolic Pathways in *Plasmodium* (an asterisk “” identifies antimalarial drug)

Metabolic Pathway	Target Site	Drug (E.C. Targets)	Start concentration in growth assays (µM)
Pyrimidine	Mitochondral E-Transport	*Atovaquone (1.10.2.2) **(1)**	1.9
Purine	Adenosine deaminase(Adenosine to Inosine)	(erythro-9-(2-hydroxy-3-nonyl)adenine) (3.5.4.4) **(2)**	1061.67
	IMP dehydrogenase(IMP to XMP) inhibits the synthesis of guanosine monophosphate from inosine monophosphate by parasitized erythrocytes	Bredinin (Mizoribine) (1.1.1.205)**(3)**	257.33
	Hypoxanthine-guanine phosphoribosyltransferase (HXP to IMP)	Allopurinol (2.4.2.8)**(4)**	2448.33
Folate	Dihydrofolate synthase (Dihydropteroate to Folate)	Pyrimethamine (1.5.1.3)**(5)**	16.76
	Dihydrofolate synthase (Dihydropteroate to Folate)	Methotrexate (1.5.1.3)**(6, 7)**	9.17
	Dihydrofolate synthase (Dihydropteroate to Folate)	Cycloguanil (1.5.1.3)**(5)**	14.37
		Sulfadoxine	5.37
Fatty Acid Synthesis in Apicoplast	Blocks the synthesis of dihydrofolic acid by inhibiting the enzyme dihydropteroate synthase	Sulfadiazine (2.5.1.15)**(8)**	3330
	Acetyl-CoA Carboxylase (Acetyl-CoA to Malonyl-CoA)	Fenoxaprop (6.4.1.2) or [2-(2,4,5-trichlorophenoxylpropionic acid)]**(9)**	1236.67
	Acetyl-CoA Carboxylase (Acetyl-CoA to Malonyl-CoA)	5-(Tetradecyloxy)-2-furoic acid (6.4.1.2)**(9)**	205.33
	Oxoacyl-ACP Synthase (Acetyl-CoA to Acetyl ACP)	Thiolactomycin (2.3.1.85, 2.3.1.41)**(10, 11)**	475.7
	Oxoacyl-ACP Synthase or fatty-acid synthase (Acetyl ACP to Oxoacyl-ACP)	Cerulenin (2.3.1.41, 2.3.1.85)**(10, 11)**	149.33
	Enoyl-ACP reductase (Enoyl-acyl-ACP to Acyl-ACP)	Triclosan (Irgasan) (1.3.1.9)**(12)**	1151.67

Metabolic Pathway	Target Site	Drug (E.C. Targets)	Start concentration in growth assays
Shikimate Biosynthesis	Phosphoshikimate-1-carboxyvinyltransferase and Enolpyruvylshikimate-3-phosphate synthase (PEP)	Glyphosate (2.5.1.19)**(13)**	4928.33
	Aminodeoxy-chorismate lyase (Glutamate metabolism and Chorismate to 4-amino-4-deoxychorismate)	6-Diazo-5-oxo-D-norleucine (6.3.5.8) **(14, 15)**	195.0
Spingomyelin and Ceremide	Serine C-palmitoyltransferase (Serine or Palmitoyl-Co-A to 3-dehydrosphinganine)	Myriocin (2.3.1.50) **(16)**	166.0
	Serine C-palmitoyltransferase (Serine or Palmitoyl-Co-A to 3-dehydrosphinganine)	D-cycloserine (2.3.1.50) **(16)**	8163.33
	Spingosine N-acyltransferase (Sphinganine to Dihydroceramice)	Fumonisin (B1) (2.3.1.24) **(16)**	233.33
	Spingosine N-acyltransferase (Sphinganine to Dihydroceramice)	Fumonisin (B2) (2.3.1.24) **(16)**	233.33
	Sphingomyelin phosphodiesterase (Sphingomyelin to Ceremide)	N- Butyldeoxynojirimycin (3.1.4.12) **(17)**	
	Ceremide cholinphosphotransferase and Ceremide glucosyltransferase (Ceremide to sphingomyelin and Glucosylceremide)	D,L-*erythro*-1-Phenyl-2-hexadecanoylamino-3-morpholino-1-propanol•HCl (2.7.8.3) **(18)**	326.0
	Ceremide cholinphosphotransferase and Ceremide glucosyltransferase (Ceremide to sphingomyelin and Glucosylceremide)	D-*threo*-1-Phenyl-2-hexadecanoylamino-3-morpholino-1-propanol•HCl (2.4.1.80) **(18)**	651.67

Metabolic Pathway	Target Site	Drug (E.C. Targets)	Start concentration in growth assays
Hemoglobin Digestion and ferriprotoporphyrin IX polymerization	Phospholipase A2<br>(Reversible inhibitor of calcium-dependent phospholipase A2)	Palmityl trifluoromethyl ketone **(19)**	540.33
	Hemozoin Polymerization	Chloroquine **(20-22)**, primaquine **(23, 24)**, mefloquine **(24)** , amodiaquine **(25, 26)**, piperaquine **(22)**.	16.2, 73.3, 2.2, 1.8, 0.8
	Cysteine proteases inhibitor	E-64 (3.4.22.-) **(27)**	93.33
	Aspartic acid proteases inhibitor	Pepstatin A (3.4.23.38) **(28)**	48.67
Methionine and Polyamine	Adenosylhomocysteinease (S-adenosylhomocysteinase to Adenosine)	Sinefungin (3.4.23.39) **(29)**	174.67
	Adenosylhomocysteinease (S-adenosylhomocysteinase to Adenosine)	2-Fluoroadenosine (3.3.1.1, 2.4.2.1) **(30, 31)**	233.67
	DOXP to Isopentyl-PP (DOXP reductoisomerase) Inhibits farnesyl diphosphate synthase	Ibandronate (1.1.1.267) **(32)**	977.0
Merozoite Invasion	Actin Polymerization	Cytochalasin D **(33)**	3.28
	Interacts with Tubulin	Benzimidazole **(34)**	14108.33
	Interacts with Tubulin	2,4-Dinitroaniline **(35)**	9101.67
Mitochondrial Electron Transport Chain	Glecerol 3-Phosphate dehydrogenase (Interferes in Glycolysis esp. Glycerone-P to Glycerol-3P)	1,10-Phenenthroline (1.1.1.8) **(36)**	925.0
	Glecerol 3-Phosphate dehydrogenase (Interferes in Glycolysis esp. Glycerone-P to Glycerol-3P)	Rotenone (1.1.1.8) **(37)**	845.0
	Glecerol 3-Phosphate dehydrogenase (Interferes in Glycolysis esp. Glycerone-P to Glycerol-3P)	Amytal (1.1.1.8) or amobarbital sodium **(38)**	133.67
	Interacts with the iron-sulphur centre of the succninate dehydrogenase (Succinate to Fumerate)	2-Thenoyltrifluoroacetone (1.3.99.1) (39) **(40)**	1500.33
	Interacts with the bc1 complex of the respiratory chain	Atovaquone (1.10.2.2) **(1)**	2.28

Metabolic Pathway	Target Site	Drug (E.C. Targets)	Start concentration in growth assays
Transcription Regulation Inhibitors/HATs or HDACs	Histone deacetylases (HDACs)	Trichostatin A **(41, 42)**	2.51
	Histone acetyltransferases (HATs) (p300/CREB-binding Protein-specific Inhibitor of Acetyltransferase, Represses the Acetylation of Histone/Nonhistone Proteins and Histone Acetyltransferase-dependent Chromatin Transcription).	Curcumin **(43)**	2262.67
DNA/RNA synthesis	Substrate for Pgp, MRP-1 and BCRP. Sequestered into lysosomes according to a pH partitioning type mechanism. Strong inhibitor of DNA and RNA synthesis.	Daunorubicin **(44)**	59.33
Cell Signaling	The CsA-cyclophilin or FK-506-FKBP complex regulates the activities of calcium dependent PP2B (serine/threonine phosphatase)	Cyclosporin A **(45, 46)**	138.33
		FK-506 **(47, 48)**	202.67
Protein synthesis	Binds reversibly to 50S ribosomal subunit causing blockade of transpeptidation/translocation reactions, inhibition of protein synthesis and thus inhibition of cell growth.	Cis-mirincamycin, trans-miricamycin **(49-51)**	1.8, 1.8
Endoperoxides	Likely mode of action is ion-dependent alkylation (principally by Fe^{++}), and the sarcoplasmic endoplasmic reticulum calcium adenosine triphosphatase (PfATPase 6) has been proposed as the primary target.	Artesunate, artelinic acid, artemether, artemisnin (qinghaosu), dihydroartemisnin **(52)**	6.5, 4.0, 2.8, 3.0, 2.9
Membrane transporters/ efflux pumps	Verapamil block voltage-dependent calcium channels. Also known as resistance reversal agents in *Plasmodium*. Desipramine electively blocks reuptake of norepinephrine (noradrenaline) from the neuronal synapse. It also inhibits serotonin reuptake	Verapamil **(53, 54)** and Desipramine **(55, 56)**	3.7, 6.3
Unknowns	Might interfere with the fatty acid metabolism of the parasite	Lumifantrine **(57)**	1.6

*An asterisk identified drugs and compounds for which the MOA has been established in *Plasmodium*.

References:

1. Fry M & Pudney M (1992) Site of action of the antimalarial hydroxynaphthoquinone, 2-[trans-4-(4'-chlorophenyl) cyclohexyl]-3-hydroxy-1,4-naphthoquinone (566C80). *Biochemical pharmacology* 43(7):1545-1553.
2. Tyler PC, Taylor EA, Frohlich RF, & Schramm VL (2007) Synthesis of 5'-methylthio coformycins: specific inhibitors for malarial adenosine deaminase. *J Am Chem Soc* 129(21):6872-6879.
3. Webster HK & Whaun JM (1982) Antimalarial properties of bredinin. Prediction based on identification of differences in human host-parasite purine metabolism. *J Clin Invest* 70(2):461-469.
4. Dawson PA, Cochran DA, Emmerson BT, & Gordon RB (1993) Inhibition of Plasmodium falciparum hypoxanthine-guanine phosphoribosyltransferase mRNA by antisense oligodeoxynucleotide sequence. *Mol Biochem Parasitol* 60(1):153-156.
5. Sixsmith DG, Watkins WM, Chulay JD, & Spencer HC (1984) In vitro antimalarial activity of tetrahydrofolate dehydrogenase inhibitors. *Am J Trop Med Hyg* 33(5):772-776.
6. Galivan J, Inglese J, McGuire JJ, Nimec Z, & Coward JK (1985) gamma-Fluoromethotrexate: synthesis and biological activity of a potent inhibitor of dihydrofolate reductase with greatly diminished ability to form poly-gamma-glutamates. *Proceedings of the National Academy of Sciences of the United States of America* 82(9):2598-2602.
7. Barnes MJ*, et al.* (1999) Impact of polyglutamation on sensitivity to raltitrexed and methotrexate in relation to drug-induced inhibition of de novo thymidylate and purine biosynthesis in CCRF-CEM cell lines. *Clin Cancer Res* 5(9):2548-2558.
8. Triglia T & Cowman AF (1994) Primary structure and expression of the dihydropteroate synthetase gene of Plasmodium falciparum. *Proceedings of the National Academy of Sciences of the United States of America* 91(15):7149-7153.
9. Gornicki P (2003) Apicoplast fatty acid biosynthesis as a target for medical intervention in apicomplexan parasites. *Int J Parasitol* 33(9):885-896.
10. Jones SM*, et al.* (2005) Analogues of thiolactomycin as potential antimalarial agents. *J Med Chem* 48(19):5932-5941.
11. Lack G, Homberger-Zizzari E, Folkers G, Scapozza L, & Perozzo R (2006) Recombinant expression and biochemical characterization of the unique elongating beta-ketoacyl-acyl carrier protein synthase involved in fatty acid biosynthesis of Plasmodium falciparum using natural and artificial substrates. *J Biol Chem* 281(14):9538-9546.
12. Tasdemir D*, et al.* (2005) Anti-protozoal and plasmodial FabI enzyme inhibiting metabolites of Scrophularia lepidota roots. *Phytochemistry* 66(3):355-362.
13. Schonbrunn E*, et al.* (2001) Interaction of the herbicide glyphosate with its target enzyme 5-enolpyruvylshikimate 3-phosphate synthase in atomic detail. *Proceedings of the National Academy of Sciences of the United States of America* 98(4):1376-1380.
14. Coggin JH, Jr. & Martin WR (1965) 6-Diazo-5-Oxo-L-Norleucine Inhibition of Escherichia Coli. *J Bacteriol* 89:1348-1353.

15. Huber KR, Mayer EP, Mitchell DF, & Roberts J (1987) Cell cycle phase perturbations by 6-diazo-5-oxo-L-norleucine and acivicin in normal and neoplastic human cell lines. *Br J Cancer* 55(6):653-656.
16. Gerold P & Schwarz RT (2001) Biosynthesis of glycosphingolipids de-novo by the human malaria parasite Plasmodium falciparum. *Mol Biochem Parasitol* 112(1):29-37.
17. Platt FM, Neises GR, Karlsson GB, Dwek RA, & Butters TD (1994) N-butyldeoxygalactonojirimycin inhibits glycolipid biosynthesis but does not affect N-linked oligosaccharide processing. *J Biol Chem* 269(43):27108-27114.
18. Lauer SA, Ghori N, & Haldar K (1995) Sphingolipid synthesis as a target for chemotherapy against malaria parasites. *Proceedings of the National Academy of Sciences of the United States of America* 92(20):9181-9185.
19. Ackermann EJ, Conde-Frieboes K, & Dennis EA (1995) Inhibition of macrophage Ca(2+)-independent phospholipase A2 by bromoenol lactone and trifluoromethyl ketones. *J Biol Chem* 270(1):445-450.
20. Homewood CA, Warhurst DC, Peters W, & Baggaley VC (1972) Lysosomes, pH and the anti-malarial action of chloroquine. *Nature* 235(5332):50-52.
21. Krogstad DJ & Schlesinger PH (1986) A perspective on antimalarial action: effects of weak bases on Plasmodium falciparum. *Biochemical pharmacology* 35(4):547-552.
22. Warhurst DC*, et al.* (2007) Activity of piperaquine and other 4-aminoquinoline antiplasmodial drugs against chloroquine-sensitive and resistant blood-stages of Plasmodium falciparum. Role of beta-haematin inhibition and drug concentration in vacuolar water- and lipid-phases. *Biochemical pharmacology* 73(12):1910-1926.
23. Basilico N, Pagani E, Monti D, Olliaro P, & Taramelli D (1998) A microtitre-based method for measuring the haem polymerization inhibitory activity (HPIA) of antimalarial drugs. *J Antimicrob Chemother* 42(1):55-60.
24. Vennerstrom JL*, et al.* (1999) 8-Aminoquinolines active against blood stage Plasmodium falciparum in vitro inhibit hematin polymerization. *Antimicrob Agents Chemother* 43(3):598-602.
25. Bray PG, Hawley SR, & Ward SA (1996) 4-Aminoquinoline resistance of Plasmodium falciparum: insights from the study of amodiaquine uptake. *Mol Pharmacol* 50(6):1551-1558.
26. Hawley SR, Bray PG, O'Neill PM, Park BK, & Ward SA (1996) The role of drug accumulation in 4-aminoquinoline antimalarial potency. The influence of structural substitution and physicochemical properties. *Biochemical pharmacology* 52(5):723-733.
27. Bailly E, Jambou R, Savel J, & Jaureguiberry G (1992) Plasmodium falciparum: differential sensitivity in vitro to E-64 (cysteine protease inhibitor) and Pepstatin A (aspartyl protease inhibitor). *J Protozool* 39(5):593-599.
28. Moura PA, Dame JB, & Fidock DA (2009) Role of Plasmodium falciparum digestive vacuole plasmepsins in the specificity and antimalarial mode of action of cysteine and aspartic protease inhibitors. *Antimicrob Agents Chemother* 53(12):4968-4978.

29. Trager W, Tershakovec M, Chiang PK, & Cantoni GL (1980) Plasmodium falciparum: antimalarial activity in culture of sinefungin and other methylation inhibitors. *Exp Parasitol* 50(1):83-89.
30. Reddy MC*, et al.* (2008) Crystal structures of Mycobacterium tuberculosis S-adenosyl-L-homocysteine hydrolase in ternary complex with substrate and inhibitors. *Protein Sci* 17(12):2134-2144.
31. Chaudhary K, Ting LM, Kim K, & Roos DS (2006) Toxoplasma gondii purine nucleoside phosphorylase biochemical characterization, inhibitor profiles, and comparison with the Plasmodium falciparum ortholog. *J Biol Chem* 281(35):25652-25658.
32. Kuntz L*, et al.* (2005) Isoprenoid biosynthesis as a target for antibacterial and antiparasitic drugs: phosphonohydroxamic acids as inhibitors of deoxyxylulose phosphate reducto-isomerase. *Biochem J* 386(Pt 1):127-135.
33. Sampath P & Pollard TD (1991) Effects of cytochalasin, phalloidin, and pH on the elongation of actin filaments. *Biochemistry* 30(7):1973-1980.
34. Skinner-Adams TS, Davis TM, Manning LS, & Johnston WA (1997) The efficacy of benzimidazole drugs against Plasmodium falciparum in vitro. *Trans R Soc Trop Med Hyg* 91(5):580-584.
35. Fennell BJ, Naughton JA, Dempsey E, & Bell A (2006) Cellular and molecular actions of dinitroaniline and phosphorothioamidate herbicides on Plasmodium falciparum: tubulin as a specific antimalarial target. *Mol Biochem Parasitol* 145(2):226-238.
36. Wijayanti MA*, et al.* (2010) Additive in vitro antiplasmodial effect of N-alkyl and N-benzyl-1,10-phenanthroline derivatives and cysteine protease inhibitor e64. *Malar Res Treat* 2010:540786.
37. Uyemura SA, Luo S, Vieira M, Moreno SN, & Docampo R (2004) Oxidative phosphorylation and rotenone-insensitive malate- and NADH-quinone oxidoreductases in Plasmodium yoelii yoelii mitochondria in situ. *J Biol Chem* 279(1):385-393.
38. Gero AM, Brown GV, & O'Sullivan WJ (1984) Pyrimidine de novo synthesis during the life cycle of the intraerythrocytic stage of Plasmodium falciparum. *J Parasitol* 70(4):536-541.
39. Suraveratum N, Krungkrai SR, Leangaramgul P, Prapunwattana P, & Krungkrai J (2000) Purification and characterization of Plasmodium falciparum succinate dehydrogenase. *Mol Biochem Parasitol* 105(2):215-222.
40. Byun HO, Kim HY, Lim JJ, Seo YH, & Yoon G (2008) Mitochondrial dysfunction by complex II inhibition delays overall cell cycle progression via reactive oxygen species production. *J Cell Biochem* 104(5):1747-1759.
41. Andrews KT*, et al.* (2012) Comparative gene expression profiling of P. falciparum malaria parasites exposed to three different histone deacetylase inhibitors. *PLoS One* 7(2):e31847.
42. Andrews KT, Haque A, & Jones MK (2012) HDAC inhibitors in parasitic diseases. *Immunol Cell Biol* 90(1):66-77.

43. Balasubramanyam K*, et al.* (2004) Curcumin, a novel p300/CREB-binding protein-specific inhibitor of acetyltransferase, represses the acetylation of histone/nonhistone proteins and histone acetyltransferase-dependent chromatin transcription. *J Biol Chem* 279(49):51163-51171.
44. Pradhan A & Tuteja R (2006) Plasmodium falciparum DNA helicase 60. dsRNA- and antibody-mediated inhibition of malaria parasite growth and downregulation of its enzyme activities by DNA-interacting compounds. *FEBS J* 273(15):3545-3556.
45. Matsuda S & Koyasu S (2000) Mechanisms of action of cyclosporine. *Immunopharmacology* 47(2-3):119-125.
46. Gavigan CS, Kiely SP, Hirtzlin J, & Bell A (2003) Cyclosporin-binding proteins of Plasmodium falciparum. *Int J Parasitol* 33(9):987-996.
47. Kotaka M*, et al.* (2008) Crystal structure of the FK506 binding domain of Plasmodium falciparum FKBP35 in complex with FK506. *Biochemistry* 47(22):5951-5961.
48. Monaghan P & Bell A (2005) A Plasmodium falciparum FK506-binding protein (FKBP) with peptidyl-prolyl cis-trans isomerase and chaperone activities. *Mol Biochem Parasitol* 139(2):185-195.
49. Powers KG (1969) Activity of chlorinated lincomycin analogues against Plasmodium cynomolgi in rhesus monkeys. *Am J Trop Med Hyg* 18(4):485-490.
50. Schmidt LH (1985) Enhancement of the curative activity of primaquine by concomitant administration of mirincamycin. *Antimicrob Agents Chemother* 27(2):151-157.
51. Khemawoot P*, et al.* (2011) Absolute bioavailability of cis-mirincamycin and trans-mirincamycin in healthy rhesus monkeys and ex vivo antimalarial activity against Plasmodium falciparum. *Antimicrob Agents Chemother* 55(12):5881-5886.
52. White NJ (2008) Qinghaosu (artemisinin): the price of success. *Science* 320(5874):330-334.
53. Martin SK, Oduola AM, & Milhous WK (1987) Reversal of chloroquine resistance in Plasmodium falciparum by verapamil. *Science* 235(4791):899-901.
54. Martiney JA, Cerami A, & Slater AF (1995) Verapamil reversal of chloroquine resistance in the malaria parasite Plasmodium falciparum is specific for resistant parasites and independent of the weak base effect. *J Biol Chem* 270(38):22393-22398.
55. Ridley RG (1998) Malaria: dissecting chloroquine resistance. *Curr Biol* 8(10):R346-349.
56. Bitonti AJ*, et al.* (1988) Reversal of chloroquine resistance in malaria parasite Plasmodium falciparum by desipramine. *Science* 242(4883):1301-1303.
57. Mwai L*, et al.* (2012) Genome wide adaptations of Plasmodium falciparum in response to lumefantrine selective drug pressure. *PLoS One* 7(2):e31623.

52. White NJ (2008) Qinghaosu (artemisinin): the price of success. (Translated from eng) *Science* 320(5874):330-334 (in eng).
53. Martin SK, Oduola AM, & Milhous WK (1987) Reversal of chloroquine resistance in Plasmodium falciparum by verapamil. (Translated from eng) *Science* 235(4791):899-901 (in eng).
54. Martiney JA, Cerami A, & Slater AF (1995) Verapamil reversal of chloroquine resistance in the malaria parasite Plasmodium falciparum is specific for resistant parasites and independent of the weak base effect. (Translated from eng) *J Biol Chem* 270(38):22393-22398 (in eng).
55. Ridley RG (1998) Malaria: dissecting chloroquine resistance. (Translated from eng) *Curr Biol* 8(10):R346-349 (in eng).
56. Bitonti AJ*, et al.* (1988) Reversal of chloroquine resistance in malaria parasite Plasmodium falciparum by desipramine. (Translated from eng) *Science* 242(4883):1301-1303 (in eng).
57. Mwai L*, et al.* (2012) Genome wide adaptations of Plasmodium falciparum in response to lumefantrine selective drug pressure. (Translated from eng) *PloS one* 7(2):e31623 (in eng).

TABLE S3

SUMMARY OF HIGH-THROUGHPUT FORWARD GENETICS SCREEN

All assays were conducted in batches of 10 mutants with Wild Type(NF54) in every batch.
All mutant values are represented as Fold Change derived from the wild type batchwise.

INHIBITOR	Gene ID/Mutant		GI50	R^2
ATQ	PF3D7_0811300	=	0.43	0.93
ATQ	PF3D7_1305500	=	0.39	0.99
ATQ	PF3D7_0615900	=	1.53	0.91
ATQ	PF3D7_1122900	=	0.40	0.91
ATQ	PF3D7_1141900	=	0.54	0.98
ATQ	PF3D7_1475600	=	0.92	0.95
ATQ	PF3D7_0211100	=	3.08	1.00
ATQ	PF3D7_0203000	=	0.25	0.75
ATQ	PF3D7_0404600	=	3.51	0.99
ATQ	PF3D7_0416500	=	2.86	0.87
ATQ	PF3D7_1018400	=	0.64	0.95
ATQ	PF3D7_0511500	=	1.67	0.96
ATQ	PF3D7_1360200	=	1.85	0.95
ATQ	PF3D7_0611800	=	0.68	0.95
ATQ	PF3D7_0619900	=	4.90	0.99
ATQ	PF3D7_0521900	=	3.77	0.98
ATQ	PF3D7_1219300	=	0.74	0.88
ATQ	PF3D7_1133700	=	0.85	0.94
ATQ	PF3D7_0808700	=	0.44	0.92
ATQ	PF3D7_1136000	=	1.52	0.97
ATQ	PF3D7_0931000	=	4.11	0.95
ATQ	PF3D7_0830900	=	2.15	0.91
ATQ	PF3D7_1231800	=	14.15	0.92
ATQ	PF3D7_1035800	=	7.03	0.93
ATQ	PF3D7_1444100	=	5.21	0.90
ATQ	PF3D7_1138900	=	4.75	0.91
ATQ	PF3D7_1441800	=	0.27	0.80
ATQ	PF3D7_1345800	=	0.52	0.91
ATQ	PF3D7_1432400	=	1.31	0.97
ATQ	PF3D7_0819800	=	0.25	0.92
ATQ	PF3D7_1207800	=	0.75	0.94
ATQ	PF3D7_1143500	=	0.52	0.97
ATQ	PF3D7_1001600	=	0.38	0.96
ATQ	PF3D7_1019400	=	0.48	0.91
ATQ	PF3D7_1311900	=	13.35	0.98
ATQ	PF3D7_1035700	=	0.31	0.96
ATQ	PF3D7_1103100	=	0.94	0.98
ATQ	PF3D7_0812500	=	0.52	0.93
ATQ	PF3D7_1216100	=	0.71	0.97
ATQ	PF3D7_0206100	=	1.17	0.97
ATQ	PF3D7_1352500	=	1.26	0.91
ATQ	PF3D7_1226900	=	0.19	0.96
ATQ	PF3D7_1368800	=	1.89	0.97
ATQ	PF3D7_1432400	=	3.29	0.99

ATQ	PF3D7_1343900	=	3.03	0.98
ATQ	PF3D7_1328800	=	1.23	0.91
ATQ	PF3D7_1138700	=	1.09	0.97
ATQ	PF3D7_1327100	=	0.12	0.92
ATQ	PF3D7_1136600	=	0.84	1.00
ATQ	PF3D7_0902200	=	0.45	0.92
ATQ	PF3D7_0727100	=	0.29	0.90
ATQ	PF3D7_0105500	=	0.22	0.85
ATQ	PF3D7_1126100	=	0.06	0.88
ATQ	PF3D7_1343700	=	0.05	0.92
ATQ	PF3D7_1330500	=	1.93	0.94
ATQ	PF3D7_1343000	=	1.33	0.92
ATQ	PF3D7_1302000	=	9.18	0.92
ATQ	PF3D7_1107900	=	0.29	0.89
ATQ	PF3D7_0606000	=	0.51	0.86
ATQ	PF3D7_0614500	=	4.12	0.88
ATQ	PF3D7_0203700	=	3.64	0.91
ATQ	PF3D7_0601700	=	5.74	0.87
ATQ	PF3D7_1001900	=	1.63	0.93
ATQ	PF3D7_1409500	=	1.22	0.92
ATQ	PF3D7_0916700	=	1.14	0.94
ATQ	PF3D7_0402000	=	0.76	0.91
ATQ	PF3D7_0102600	=	1.38	0.96
ATQ	PF3D7_1433400	=	0.73	0.98
ATQ	PF3D7_0322000	=	1.04	0.91
ATQ	PF3D7_0623900	=	0.79	0.92
ATQ	PF3D7_1013500	<	0.017 uM	0.00

MZB	PF3D7_0811300	=	1.14	0.99
MZB	PF3D7_1305500	=	0.75	0.98
MZB	PF3D7_0615900	=	0.95	0.90
MZB	PF3D7_1122900	=	0.52	0.96
MZB	PF3D7_1141900	=	1.73	0.93
MZB	PF3D7_1475600	=	0.65	0.98
MZB	PF3D7_0211100	=	1.55	0.98
MZB	PF3D7_0203000	=	1.18	0.88
MZB	PF3D7_0404600	=	0.66	0.98
MZB	PF3D7_0416500	=	1.64	0.98
MZB	PF3D7_1018400	=	1.63	1.00
MZB	PF3D7_0511500	=	2.43	1.00
MZB	PF3D7_1360200	=	1.54	0.99
MZB	PF3D7_0611800	=	1.25	0.97
MZB	PF3D7_0619900	=	1.21	0.99
MZB	PF3D7_0521900	=	1.34	0.97
MZB	PF3D7_1219300	=	1.59	0.99
MZB	PF3D7_1133700	=	0.71	0.97
MZB	PF3D7_0808700	=	1.45	0.97
MZB	PF3D7_1136000	=	0.94	0.96
MZB	PF3D7_0931000	=	0.77	0.92
MZB	PF3D7_0830900	=	0.52	0.96

MZB	PF3D7_1231800	=	1.60	0.97
MZB	PF3D7_1035800	=	1.19	0.95
MZB	PF3D7_1444100	=	1.68	0.99
MZB	PF3D7_1138900	=	0.42	0.89
MZB	PF3D7_1441800	=	0.52	1.00
MZB	PF3D7_1345800	=	0.63	1.00
MZB	PF3D7_1432400	=	0.83	1.00
MZB	PF3D7_0819800	=	0.65	0.99
MZB	PF3D7_1207800	=	0.75	1.00
MZB	PF3D7_1143500	=	0.92	1.00
MZB	PF3D7_1001600	=	0.51	1.00
MZB	PF3D7_1019400	=	0.25	0.99
MZB	PF3D7_1311900	=	0.54	0.99
MZB	PF3D7_1035700	=	0.53	0.99
MZB	PF3D7_1103100	=	1.37	0.98
MZB	PF3D7_0812500	=	1.01	0.96
MZB	PF3D7_1216100	=	1.61	0.99
MZB	PF3D7_0206100	=	0.62	0.97
MZB	PF3D7_1352500	=	0.67	0.98
MZB	PF3D7_1226900	=	1.12	0.99
MZB	PF3D7_1368800	=	0.57	0.99
MZB	PF3D7_1432400	=	1.65	0.97
MZB	PF3D7_1343900	=	0.93	0.92
MZB	PF3D7_1328800	=	0.47	0.97
MZB	PF3D7_1138700	=	0.37	0.99
MZB	PF3D7_1327100	=	0.38	0.99
MZB	PF3D7_1136600	=	0.32	0.99
MZB	PF3D7_0902200	=	0.15	0.96
MZB	PF3D7_0727100	=	0.06	0.86
MZB	PF3D7_0105500	=	0.25	0.99
MZB	PF3D7_1126100	=	0.08	0.97
MZB	PF3D7_1343700	=	0.15	0.99
MZB	PF3D7_1330500	=	1.70	0.99
MZB	PF3D7_1343000	=	2.07	0.98
MZB	PF3D7_1302000	=	2.07	1.00
MZB	PF3D7_1107900	=	1.32	0.97
MZB	PF3D7_0606000	=	1.37	0.99
MZB	PF3D7_0614500	=	1.24	0.99
MZB	PF3D7_0203700	=	1.37	0.99
MZB	PF3D7_0601700	=	1.38	1.00
MZB	PF3D7_1001900	=	1.03	0.99
MZB	PF3D7_1409500	=	0.98	0.99
MZB	PF3D7_0916700	=	0.51	0.96
MZB	PF3D7_0402000	=	0.55	0.98
MZB	PF3D7_0102600	=	0.92	0.97
MZB	PF3D7_1433400	=	0.89	0.99
MZB	PF3D7_0322000	=	1.04	0.99
MZB	PF3D7_0623900	=	0.80	0.98
MZB	PF3D7_1013500	=	0.02	0.98

MTX	PF3D7_0811300	=	300.00	0.98
MTX	PF3D7_1305500	=	263.95	0.98
MTX	PF3D7_0615900	=	131.40	0.89
MTX	PF3D7_1122900	=	179.07	0.94
MTX	PF3D7_1141900	=	634.88	0.89
MTX	PF3D7_1475600	=	3.60	0.90
MTX	PF3D7_0211100	=	74.42	0.93
MTX	PF3D7_0203000	=	145.35	0.97
MTX	PF3D7_0404600	=	239.53	0.96
MTX	PF3D7_0416500	=	67.83	1.00
MTX	PF3D7_1018400	=	126.55	0.99
MTX	PF3D7_0511500	=	83.99	1.00
MTX	PF3D7_1360200	=	82.69	0.99
MTX	PF3D7_0611800	=	102.10	0.96
MTX	PF3D7_0619900	=	65.79	0.97
MTX	PF3D7_0521900	=	137.86	0.99
MTX	PF3D7_1219300	=	1.45	0.98
MTX	PF3D7_1133700	=	43.31	0.98
MTX	PF3D7_0808700	=	0.80	0.92
MTX	PF3D7_1136000	=	81.14	0.98
MTX	PF3D7_0931000	=	84.98	0.99
MTX	PF3D7_0830900	=	41.74	0.96
MTX	PF3D7_1231800	=	73.43	0.97
MTX	PF3D7_1035800	=	89.36	0.96
MTX	PF3D7_1444100	=	109.98	0.95
MTX	PF3D7_1138900	=	5.00	0.96
MTX	PF3D7_1441800	=	88.20	0.99
MTX	PF3D7_1345800	=	110.58	0.96
MTX	PF3D7_1432400	=	138.79	1.00
MTX	PF3D7_0819800	=	79.26	0.94
MTX	PF3D7_1207800	=	0.91	0.92
MTX	PF3D7_1143500	=	88.73	0.95
MTX	PF3D7_1001600	=	75.38	0.76
MTX	PF3D7_1019400	=	90.16	0.98
MTX	PF3D7_1311900	=	90.44	0.98
MTX	PF3D7_1035700	=	66.36	0.94
MTX	PF3D7_1103100	=	136.19	0.99
MTX	PF3D7_0812500	=	77.29	0.97
MTX	PF3D7_1216100	=	427.92	0.98
MTX	PF3D7_0206100	=	503.84	1.00
MTX	PF3D7_1352500	=	2.03	0.99
MTX	PF3D7_1226900	=	462.16	1.00
MTX	PF3D7_1368800	=	257.21	0.99
MTX	PF3D7_1432400	=	725.94	1.00
MTX	PF3D7_1343900	=	428.73	0.99
MTX	PF3D7_1328800	=	406.40	0.99
MTX	PF3D7_1138700	=	19748.00	0.99
MTX	PF3D7_1327100	=	15717.50	0.98
MTX	PF3D7_1136600	=	14196.00	0.99
MTX	PF3D7_0902200	=	5507.00	0.97
MTX	PF3D7_0727100	=	8063.00	0.98
MTX	PF3D7_0105500	=	12051.00	0.99

MTX	PF3D7_1126100	=	2795.00	0.98
MTX	PF3D7_1343700	=	8948.00	0.99
MTX	PF3D7_1330500	=	609.96	0.99
MTX	PF3D7_1343000	=	1074.43	0.99
MTX	PF3D7_1302000	=	1.36	0.97
MTX	PF3D7_1107900	=	896.79	0.97
MTX	PF3D7_0606000	=	681.93	0.99
MTX	PF3D7_0614500	>	9170 uM	
MTX	PF3D7_0203700	>	9170 uM	
MTX	PF3D7_0601700	=	618.61	1.00
MTX	PF3D7_1001900	=	223.33	0.94
MTX	PF3D7_1409500	=	182.65	0.99
MTX	PF3D7_0916700	=	134.43	0.91
MTX	PF3D7_0402000	=	4.23	0.98
MTX	PF3D7_0102600	=	224.49	1.00
MTX	PF3D7_1433400	=	194.13	0.99
MTX	PF3D7_0322000	=	262.18	0.98
MTX	PF3D7_0623900	=	191.25	0.98
MTX	PF3D7_1013500	=	26.82	0.97
TCPA	PF3D7_0811300	=	1.35	0.99
TCPA	PF3D7_1305500	=	0.78	1.00
TCPA	PF3D7_0615900	=	1.89	0.92
TCPA	PF3D7_1122900	=	1.12	0.94
TCPA	PF3D7_1141900	=	1.22	0.94
TCPA	PF3D7_1475600	=	0.68	0.96
TCPA	PF3D7_0211100	=	0.60	0.93
TCPA	PF3D7_0203000	=	0.77	0.95
TCPA	PF3D7_0404600	=	1.63	0.99
TCPA	PF3D7_0416500	=	0.22	0.94
TCPA	PF3D7_1018400	=	1.30	0.99
TCPA	PF3D7_0511500	=	0.00	0.65
TCPA	PF3D7_1360200	=	0.53	0.97
TCPA	PF3D7_0611800	=	0.76	0.97
TCPA	PF3D7_0619900	=	0.81	0.96
TCPA	PF3D7_0521900	=	1.59	1.00
TCPA	PF3D7_1219300	=	1.78	0.99
TCPA	PF3D7_1133700	=	0.53	0.99
TCPA	PF3D7_0808700	=	12.51	0.95
TCPA	PF3D7_1136000	=	0.24	0.98
TCPA	PF3D7_0931000	=	0.36	1.00
TCPA	PF3D7_0830900	=	0.16	0.96
TCPA	PF3D7_1231800	=	0.43	1.00
TCPA	PF3D7_1035800	=	0.37	1.00
TCPA	PF3D7_1444100	=	0.40	0.98
TCPA	PF3D7_1138900	=	0.26	0.97
TCPA	PF3D7_1441800	=	0.68	0.99
TCPA	PF3D7_1345800	=	0.72	0.99
TCPA	PF3D7_1432400	=	2.05	0.97
TCPA	PF3D7_0819800	=	0.60	0.96

TCPA	PF3D7_1207800	=	0.23	0.96
TCPA	PF3D7_1143500	=	0.69	0.97
TCPA	PF3D7_1001600	=	0.80	0.98
TCPA	PF3D7_1019400	=	0.73	0.97
TCPA	PF3D7_1311900	=	0.69	0.98
TCPA	PF3D7_1035700	=	0.40	0.99
TCPA	PF3D7_1103100	=	1.37	1.00
TCPA	PF3D7_0812500	=	0.72	0.99
TCPA	PF3D7_1216100	=	0.95	0.99
TCPA	PF3D7_0206100	=	1.00	0.99
TCPA	PF3D7_1352500	=	0.50	0.99
TCPA	PF3D7_1226900	=	0.73	0.98
TCPA	PF3D7_1368800	=	0.38	0.96
TCPA	PF3D7_1432400	=	3.53	0.97
TCPA	PF3D7_1343900	=	0.72	0.99
TCPA	PF3D7_1328800	=	1.59	0.89
TCPA	PF3D7_1138700	=	0.74	0.97
TCPA	PF3D7_1327100	=	1.17	0.99
TCPA	PF3D7_1136600	=	0.60	0.98
TCPA	PF3D7_0902200	=	0.38	0.98
TCPA	PF3D7_0727100	=	0.52	0.99
TCPA	PF3D7_0105500	=	0.75	0.99
TCPA	PF3D7_1126100	=	0.09	0.97
TCPA	PF3D7_1343700	=	0.21	0.99
TCPA	PF3D7_1330500	=	1.49	1.00
TCPA	PF3D7_1343000	=	2.91	0.99
TCPA	PF3D7_1302000	=	1.67	1.00
TCPA	PF3D7_1107900	=	0.53	0.96
TCPA	PF3D7_0606000	=	1.34	0.99
TCPA	PF3D7_0614500	=	1.16	0.98
TCPA	PF3D7_0203700	=	0.42	0.97
TCPA	PF3D7_0601700	=	0.61	0.98
TCPA	PF3D7_1001900	=	1.88	0.93
TCPA	PF3D7_1409500	=	1.08	0.98
TCPA	PF3D7_0916700	=	0.67	0.96
TCPA	PF3D7_0402000	=	0.99	0.99
TCPA	PF3D7_0102600	=	1.52	0.99
TCPA	PF3D7_1433400	=	1.29	0.93
TCPA	PF3D7_0322000	=	1.04	0.97
TCPA	PF3D7_0623900	=	0.85	0.96
TCPA	PF3D7_1013500	=	1.15	0.97

TOFA	PF3D7_0811300	=	0.29	0.92
TOFA	PF3D7_1305500	=	0.81	0.98
TOFA	PF3D7_0615900	=	1.09	1.00
TOFA	PF3D7_1122900	=	0.70	0.97
TOFA	PF3D7_1141900	=	0.79	0.97
TOFA	PF3D7_1475600	=	0.75	0.97
TOFA	PF3D7_0211100	=	0.67	0.96
TOFA	PF3D7_0203000	=	4.30	0.79

TOFA	PF3D7_0404600	=	0.66	0.97
TOFA	PF3D7_0416500	=	1.54	0.99
TOFA	PF3D7_1018400	=	0.55	0.93
TOFA	PF3D7_0511500	=	0.99	0.95
TOFA	PF3D7_1360200	=	0.50	0.94
TOFA	PF3D7_0611800	=	0.70	0.97
TOFA	PF3D7_0619900	=	0.60	1.00
TOFA	PF3D7_0521900	=	1.54	0.98
TOFA	PF3D7_1219300	=	1.63	0.98
TOFA	PF3D7_1133700	=	1.32	0.95
TOFA	PF3D7_0808700	=	1.07	0.96
TOFA	PF3D7_1136000	=	0.76	0.97
TOFA	PF3D7_0931000	=	2.11	0.98
TOFA	PF3D7_0830900	=	1.90	0.98
TOFA	PF3D7_1231800	=	2.51	0.97
TOFA	PF3D7_1035800	=	2.04	0.96
TOFA	PF3D7_1444100	=	1.69	0.94
TOFA	PF3D7_1138900	=	2.10	0.97
TOFA	PF3D7_1441800	=	0.33	0.99
TOFA	PF3D7_1345800	=	0.62	0.99
TOFA	PF3D7_1432400	=	0.98	0.99
TOFA	PF3D7_0819800	=	0.50	0.98
TOFA	PF3D7_1207800	=	0.77	0.99
TOFA	PF3D7_1143500	=	0.46	1.00
TOFA	PF3D7_1001600	=	0.70	1.00
TOFA	PF3D7_1019400	=	1.29	1.00
TOFA	PF3D7_1311900	=	0.49	1.00
TOFA	PF3D7_1035700	=	0.41	1.00
TOFA	PF3D7_1103100	=	0.21	0.91
TOFA	PF3D7_0812500	=	0.51	0.99
TOFA	PF3D7_1216100	=	0.61	0.99
TOFA	PF3D7_0206100	=	0.65	0.98
TOFA	PF3D7_1352500	=	0.36	0.99
TOFA	PF3D7_1226900	=	0.76	1.00
TOFA	PF3D7_1368800	=	0.78	0.99
TOFA	PF3D7_1432400	=	0.73	0.99
TOFA	PF3D7_1343900	=	0.49	0.99
TOFA	PF3D7_1328800	=	0.75	0.95
TOFA	PF3D7_1138700	=	0.50	0.98
TOFA	PF3D7_1327100	=	0.27	0.99
TOFA	PF3D7_1136600	=	0.48	0.98
TOFA	PF3D7_0902200	=	0.58	0.99
TOFA	PF3D7_0727100	=	0.52	0.99
TOFA	PF3D7_0105500	=	0.26	0.99
TOFA	PF3D7_1126100	=	0.52	0.99
TOFA	PF3D7_1343700	=	0.27	0.99
TOFA	PF3D7_1330500	=	0.83	0.99
TOFA	PF3D7_1343000	=	0.35	0.99
TOFA	PF3D7_1302000	=	1.22	0.99
TOFA	PF3D7_1107900	=	0.48	0.99
TOFA	PF3D7_0606000	=	1.25	0.98
TOFA	PF3D7_0614500	=	1.14	0.99

TOFA	PF3D7_0203700	=	1.44	1.00
TOFA	PF3D7_0601700	=	1.00	0.99
TOFA	PF3D7_1001900	=	1.21	0.99
TOFA	PF3D7_1409500	=	0.02	0.93
TOFA	PF3D7_0916700	=	1.31	0.98
TOFA	PF3D7_0402000	=	1.02	0.98
TOFA	PF3D7_0102600	=	1.18	1.00
TOFA	PF3D7_1433400	=	0.75	0.99
TOFA	PF3D7_0322000	=	1.17	1.00
TOFA	PF3D7_0623900	=	1.02	0.98
TOFA	PF3D7_1013500	=	6.55	0.97

CRL	PF3D7_0811300		0.02	0.99
CRL	PF3D7_1305500	=	1.13	0.99
CRL	PF3D7_0615900	=	1.21	0.91
CRL	PF3D7_1122900	=	0.76	0.91
CRL	PF3D7_1141900	=	1.13	0.92
CRL	PF3D7_1475600	=	0.28	0.92
CRL	PF3D7_0211100	=	0.38	0.89
CRL	PF3D7_0203000	=	0.65	0.93
CRL	PF3D7_0404600	=	1.50	0.99
CRL	PF3D7_0416500	=	1.17	0.98
CRL	PF3D7_1018400	=	2.33	0.97
CRL	PF3D7_0511500	=	1.47	0.99
CRL	PF3D7_1360200	=	2.10	0.99
CRL	PF3D7_0611800	=	1.11	0.98
CRL	PF3D7_0619900	=	0.68	0.97
CRL	PF3D7_0521900	=	4.44	0.97
CRL	PF3D7_1219300	=	2.44	0.97
CRL	PF3D7_1133700	=	1.12	0.97
CRL	PF3D7_0808700	=	2.42	0.97
CRL	PF3D7_1136000	=	0.75	0.92
CRL	PF3D7_0931000	=	2.06	0.99
CRL	PF3D7_0830900	=	0.74	0.93
CRL	PF3D7_1231800	=	3.77	0.96
CRL	PF3D7_1035800	=	2.73	0.93
CRL	PF3D7_1444100	=	4.92	0.93
CRL	PF3D7_1138900	=	2.29	0.93
CRL	PF3D7_1441800	=	0.64	0.97
CRL	PF3D7_1345800	=	0.98	0.91
CRL	PF3D7_1432400	=	1.25	0.97
CRL	PF3D7_0819800	=	0.68	0.97
CRL	PF3D7_1207800	=	0.13	0.93
CRL	PF3D7_1143500	=	0.28	0.96
CRL	PF3D7_1001600	=	0.17	0.91
CRL	PF3D7_1019400	=	0.66	0.98
CRL	PF3D7_1311900	=	0.49	0.98
CRL	PF3D7_1035700	=	0.47	0.93
CRL	PF3D7_1103100	=	0.98	0.99
CRL	PF3D7_0812500	=	0.52	0.96

CRL	PF3D7_1216100	=	0.53	0.96
CRL	PF3D7_0206100	=	0.96	0.95
CRL	PF3D7_1352500	=	1.13	0.97
CRL	PF3D7_1226900	=	1.56	0.98
CRL	PF3D7_1368800	=	0.29	0.96
CRL	PF3D7_1432400	=	2.13	0.97
CRL	PF3D7_1343900	=	0.97	0.98
CRL	PF3D7_1328800	=	1.07	0.96
CRL	PF3D7_1138700	=	0.32	0.98
CRL	PF3D7_1327100	=	0.13	0.99
CRL	PF3D7_1136600	=	0.16	0.99
CRL	PF3D7_0902200	=	0.06	0.98
CRL	PF3D7_0727100	=	0.06	0.97
CRL	PF3D7_0105500	=	0.19	0.98
CRL	PF3D7_1126100	=	0.01	0.97
CRL	PF3D7_1343700	=	0.05	0.98
CRL	PF3D7_1330500	=	2.54	0.98
CRL	PF3D7_1343000	=	6.32	0.99
CRL	PF3D7_1302000	=	4.23	0.99
CRL	PF3D7_1107900	=	2.26	0.99
CRL	PF3D7_0606000	=	4.48	0.99
CRL	PF3D7_0614500	=	3.03	0.99
CRL	PF3D7_0203700	=	1.40	0.99
CRL	PF3D7_0601700	=	3.82	0.99
CRL	PF3D7_1001900	=	0.99	0.99
CRL	PF3D7_1409500	=	0.75	1.00
CRL	PF3D7_0916700	=	0.65	0.95
CRL	PF3D7_0402000	=	0.92	1.00
CRL	PF3D7_0102600	=	1.41	1.00
CRL	PF3D7_1433400	=	0.85	0.95
CRL	PF3D7_0322000	=	0.99	0.99
CRL	PF3D7_0623900	=	0.90	0.98
CRL	PF3D7_1013500	=	0.33	0.98

TRI	PF3D7_0811300	=	0.15	0.96
TRI	PF3D7_1305500	=	0.14	0.97
TRI	PF3D7_0615900	=	0.57	0.99
TRI	PF3D7_1122900	=	0.12	0.97
TRI	PF3D7_1141900	=	0.08	0.95
TRI	PF3D7_1475600	=	0.30	0.97
TRI	PF3D7_0211100	=	0.52	0.97
TRI	PF3D7_0203000	=	2.55	0.84
TRI	PF3D7_0404600	=	6.30	0.94
TRI	PF3D7_0416500	=	1.10	0.99
TRI	PF3D7_1018400	=	0.54	0.97
TRI	PF3D7_0511500	=	4.43	0.96
TRI	PF3D7_1360200	=	0.42	0.98
TRI	PF3D7_0611800	=	2.72	0.95
TRI	PF3D7_0619900	=	1.38	0.98
TRI	PF3D7_0521900	=	3.08	0.97

TRI	PF3D7_1219300	=	1.32	0.99
TRI	PF3D7_1133700	=	6.03	0.97
TRI	PF3D7_0808700	=	3.50	0.96
TRI	PF3D7_1136000	=	3.91	0.93
TRI	PF3D7_0931000	=	8.89	0.99
TRI	PF3D7_0830900	=	5.75	0.96
TRI	PF3D7_1231800	=	8.82	0.96
TRI	PF3D7_1035800	=	1.35	0.96
TRI	PF3D7_1444100	=	1.94	0.98
TRI	PF3D7_1138900	=	3.87	0.96
TRI	PF3D7_1441800	=	3.31	0.94
TRI	PF3D7_1345800	=	46.21	0.94
TRI	PF3D7_1432400	=	1.60	0.92
TRI	PF3D7_0819800	=	0.82	0.92
TRI	PF3D7_1207800	=	0.86	0.90
TRI	PF3D7_1143500	=	1.93	0.94
TRI	PF3D7_1001600	=	0.63	0.92
TRI	PF3D7_1019400	=	6.41	0.89
TRI	PF3D7_1311900	=	0.24	0.92
TRI	PF3D7_1035700	=	0.86	0.96
TRI	PF3D7_1103100	=	0.37	0.97
TRI	PF3D7_0812500	=	0.56	0.98
TRI	PF3D7_1216100	=	1.16	0.94
TRI	PF3D7_0206100	=	2.03	0.94
TRI	PF3D7_1352500	=	1.67	0.94
TRI	PF3D7_1226900	=	0.37	0.89
TRI	PF3D7_1368800	=	1.41	0.96
TRI	PF3D7_1432400	=	1.86	0.93
TRI	PF3D7_1343900	=	2.05	0.92
TRI	PF3D7_1328800	=	0.47	0.97
TRI	PF3D7_1138700	=	0.93	0.98
TRI	PF3D7_1327100	=	0.22	0.99
TRI	PF3D7_1136600	=	0.74	0.96
TRI	PF3D7_0902200	=	0.45	0.98
TRI	PF3D7_0727100	=	0.23	0.98
TRI	PF3D7_0105500	=	0.05	0.96
TRI	PF3D7_1126100	=	0.03	0.88
TRI	PF3D7_1343700	=	0.06	0.95
TRI	PF3D7_1330500	=	0.58	0.99
TRI	PF3D7_1343000	=	0.75	0.97
TRI	PF3D7_1302000	=	1.71	0.98
TRI	PF3D7_1107900	=	0.27	0.97
TRI	PF3D7_0606000	=	0.98	0.97
TRI	PF3D7_0614500	=	6.78	0.99
TRI	PF3D7_0203700	=	0.71	0.95
TRI	PF3D7_0601700	=	1.20	0.94
TRI	PF3D7_1001900	=	1.10	0.98
TRI	PF3D7_1409500	=	0.31	0.96
TRI	PF3D7_0916700	=	0.06	0.93
TRI	PF3D7_0402000	=	0.62	0.94
TRI	PF3D7_0102600	=	0.03	0.97
TRI	PF3D7_1433400	=	0.17	0.97

TRI	PF3D7_0322000	=	0.33	0.89
TRI	PF3D7_0623900	=	0.31	0.84
TRI	PF3D7_1013500	=	0.01	0.94
CYC	PF3D7_0811300	=	21.85	0.96
CYC	PF3D7_1305500	=	0.87	0.98
CYC	PF3D7_0615900	=	0.55	0.86
CYC	PF3D7_1122900	=	0.60	0.95
CYC	PF3D7_1141900	=	0.47	0.89
CYC	PF3D7_1475600	=	0.66	0.89
CYC	PF3D7_0211100	=	0.30	0.90
CYC	PF3D7_0203000	=	0.55	0.99
CYC	PF3D7_0404600	=	1.36	0.99
CYC	PF3D7_0416500	=	0.88	0.93
CYC	PF3D7_1018400	=	1.29	0.96
CYC	PF3D7_0511500	=	1.09	0.99
CYC	PF3D7_1360200	=	1.63	0.98
CYC	PF3D7_0611800	=	0.28	0.83
CYC	PF3D7_0619900	=	1.79	0.94
CYC	PF3D7_0521900	=	1.49	0.99
CYC	PF3D7_1219300	=	2.22	0.99
CYC	PF3D7_1133700	=	0.88	0.98
CYC	PF3D7_0808700	=	1.07	0.95
CYC	PF3D7_1136000	=	1.63	0.94
CYC	PF3D7_0931000	=	1.34	0.94
CYC	PF3D7_0830900	=	0.76	0.84
CYC	PF3D7_1231800	=	2.70	0.97
CYC	PF3D7_1035800	=	0.90	0.91
CYC	PF3D7_1444100	=	2.75	0.97
CYC	PF3D7_1138900	=	1.22	0.96
CYC	PF3D7_1441800	=	0.71	0.95
CYC	PF3D7_1345800	=	1.17	0.98
CYC	PF3D7_1432400	=	1.27	0.85
CYC	PF3D7_0819800	=	0.34	0.99
CYC	PF3D7_1207800	=	0.10	0.92
CYC	PF3D7_1143500	=	0.60	0.95
CYC	PF3D7_1001600	=	0.18	0.86
CYC	PF3D7_1019400	=	0.92	0.93
CYC	PF3D7_1311900	=	0.71	0.98
CYC	PF3D7_1035700	=	1.13	0.92
CYC	PF3D7_1103100	=	2.05	0.99
CYC	PF3D7_0812500	=	1.13	0.98
CYC	PF3D7_1216100	=	1.04	0.99
CYC	PF3D7_0206100	=	3.10	1.00
CYC	PF3D7_1352500	=	0.75	0.98
CYC	PF3D7_1226900	=	3.03	0.98
CYC	PF3D7_1368800	=	0.26	0.95
CYC	PF3D7_1432400	=	2.14	0.99
CYC	PF3D7_1343900	=	0.93	0.98
CYC	PF3D7_1328800	=	0.81	0.99

CYC	PF3D7_1138700	=	0.36	0.97
CYC	PF3D7_1327100	=	0.37	0.92
CYC	PF3D7_1136600	=	0.39	0.94
CYC	PF3D7_0902200	=	0.07	0.94
CYC	PF3D7_0727100	=	0.24	0.91
CYC	PF3D7_0105500	=	0.21	0.97
CYC	PF3D7_1126100	=	0.16	0.93
CYC	PF3D7_1343700	=	0.28	0.89
CYC	PF3D7_1330500	=	1.00	0.99
CYC	PF3D7_1343000	=	3.17	0.99
CYC	PF3D7_1302000	=	2.44	0.99
CYC	PF3D7_1107900	=	1.86	0.98
CYC	PF3D7_0606000	=	1.30	0.99
CYC	PF3D7_0614500	=	1.46	1.00
CYC	PF3D7_0203700	=	1.64	1.00
CYC	PF3D7_0601700	=	1.51	0.99
CYC	PF3D7_1001900	=	1.33	0.91
CYC	PF3D7_1409500	=	1.18	0.89
CYC	PF3D7_0916700	=	0.86	0.99
CYC	PF3D7_0402000	=	0.52	0.98
CYC	PF3D7_0102600	=	2.74	0.86
CYC	PF3D7_1433400	=	2.59	0.97
CYC	PF3D7_0322000	=	1.24	0.97
CYC	PF3D7_0623900	=	2.03	0.98
CYC	PF3D7_1013500	=	1.28	0.96

E-PPMP	PF3D7_0811300	=	0.31	0.93
E-PPMP	PF3D7_1305500	=	0.21	0.97
E-PPMP	PF3D7_0615900	=	0.47	0.97
E-PPMP	PF3D7_1122900	=	0.20	0.99
E-PPMP	PF3D7_1141900	=	0.61	0.96
E-PPMP	PF3D7_1475600	=	0.42	0.98
E-PPMP	PF3D7_0211100	=	1.59	0.94
E-PPMP	PF3D7_0203000	>	326 uM	
E-PPMP	PF3D7_0404600	=	0.42	0.97
E-PPMP	PF3D7_0416500	=	2.35	0.95
E-PPMP	PF3D7_1018400	=	1.11	0.91
E-PPMP	PF3D7_0511500	=	1.52	0.94
E-PPMP	PF3D7_1360200	=	0.67	0.92
E-PPMP	PF3D7_0611800	=	1.51	0.98
E-PPMP	PF3D7_0619900	=	0.46	0.99
E-PPMP	PF3D7_0521900	=	1.97	0.94
E-PPMP	PF3D7_1219300	=	1.34	0.97
E-PPMP	PF3D7_1133700	=	1.99	0.99
E-PPMP	PF3D7_0808700	=	0.38	0.98
E-PPMP	PF3D7_1136000	=	1.16	0.99
E-PPMP	PF3D7_0931000	=	2.69	0.99
E-PPMP	PF3D7_0830900	=	2.48	0.96
E-PPMP	PF3D7_1231800	=	3.71	0.97
E-PPMP	PF3D7_1035800	=	1.87	0.99

E-PPMP	PF3D7_1444100	=	3.78	0.96
E-PPMP	PF3D7_1138900	=	4.43	0.99
E-PPMP	PF3D7_1441800	=	0.50	0.98
E-PPMP	PF3D7_1345800	=	0.58	0.99
E-PPMP	PF3D7_1432400	=	0.70	0.99
E-PPMP	PF3D7_0819800	=	0.41	1.00
E-PPMP	PF3D7_1207800	=	0.84	1.00
E-PPMP	PF3D7_1143500	=	0.23	0.99
E-PPMP	PF3D7_1001600	=	0.60	1.00
E-PPMP	PF3D7_1019400	=	3.93	1.00
E-PPMP	PF3D7_1311900	=	0.67	0.99
E-PPMP	PF3D7_1035700	=	0.72	1.00
E-PPMP	PF3D7_1103100	=	0.36	0.95
E-PPMP	PF3D7_0812500	=	0.37	0.98
E-PPMP	PF3D7_1216100	=	0.17	0.99
E-PPMP	PF3D7_0206100	=	0.40	0.97
E-PPMP	PF3D7_1352500	=	0.11	0.99
E-PPMP	PF3D7_1226900	=	0.60	0.99
E-PPMP	PF3D7_1368800	=	0.30	0.99
E-PPMP	PF3D7_1432400	=	0.19	0.99
E-PPMP	PF3D7_1343900	=	0.32	0.98
E-PPMP	PF3D7_1328800	=	0.37	0.96
E-PPMP	PF3D7_1138700	=	0.18	0.98
E-PPMP	PF3D7_1327100	=	0.04	0.97
E-PPMP	PF3D7_1136600	=	0.17	0.98
E-PPMP	PF3D7_0902200	=	0.57	0.99
E-PPMP	PF3D7_0727100	=	0.69	0.99
E-PPMP	PF3D7_0105500	=	0.04	0.97
E-PPMP	PF3D7_1126100	=	0.31	0.97
E-PPMP	PF3D7_1343700	=	0.07	0.96
E-PPMP	PF3D7_1330500	=	0.98	0.98
E-PPMP	PF3D7_1343000	=	0.56	0.93
E-PPMP	PF3D7_1302000	=	1.72	0.97
E-PPMP	PF3D7_1107900	=	0.31	0.95
E-PPMP	PF3D7_0606000	=	0.84	0.98
E-PPMP	PF3D7_0614500	=	0.94	0.97
E-PPMP	PF3D7_0203700	=	0.49	0.91
E-PPMP	PF3D7_0601700	=	1.32	0.98
E-PPMP	PF3D7_1001900	=	0.96	0.98
E-PPMP	PF3D7_1409500	=	0.11	0.99
E-PPMP	PF3D7_0916700	=	0.80	0.98
E-PPMP	PF3D7_0402000	=	0.89	0.99
E-PPMP	PF3D7_0102600	=	1.13	0.99
E-PPMP	PF3D7_1433400	=	1.18	0.99
E-PPMP	PF3D7_0322000	=	1.06	0.99
E-PPMP	PF3D7_0623900	=	0.97	0.98
E-PPMP	PF3D7_1013500	=	3.06	0.93
T-PPMP	PF3D7_0811300	=	90.73	0.94
T-PPMP	PF3D7_1305500	=	1.45	0.95

T-PPMP	PF3D7_0615900	=	38.39	0.97
T-PPMP	PF3D7_1122900	=	153.41	0.98
T-PPMP	PF3D7_1141900	=	110.37	0.97
T-PPMP	PF3D7_1475600	=	68.34	0.94
T-PPMP	PF3D7_0211100	=	0.39	0.90
T-PPMP	PF3D7_0203000	=	0.84	0.92
T-PPMP	PF3D7_0404600	=	5.08	0.88
T-PPMP	PF3D7_0416500	=	0.91	0.98
T-PPMP	PF3D7_1018400	=	1.92	0.92
T-PPMP	PF3D7_0511500	=	7.20	0.96
T-PPMP	PF3D7_1360200	=	0.81	0.96
T-PPMP	PF3D7_0611800	=	2.27	0.95
T-PPMP	PF3D7_0619900	=	0.17	0.97
T-PPMP	PF3D7_0521900	=	1.74	0.96
T-PPMP	PF3D7_1219300	=	1.90	0.87
T-PPMP	PF3D7_1133700	=	2.58	0.95
T-PPMP	PF3D7_0808700	=	3.32	0.98
T-PPMP	PF3D7_1136000	=	4.68	0.99
T-PPMP	PF3D7_0931000	=	9.21	0.92
T-PPMP	PF3D7_0830900	=	1.31	0.88
T-PPMP	PF3D7_1231800	=	4.15	0.93
T-PPMP	PF3D7_1035800	=	2.77	0.88
T-PPMP	PF3D7_1444100	=	3.56	0.91
T-PPMP	PF3D7_1138900	=	2.71	0.91
T-PPMP	PF3D7_1441800	=	0.31	0.96
T-PPMP	PF3D7_1345800	=	0.12	0.88
T-PPMP	PF3D7_1432400	=	0.88	0.95
T-PPMP	PF3D7_0819800	=	0.83	0.96
T-PPMP	PF3D7_1207800	=	0.44	0.81
T-PPMP	PF3D7_1143500	=	0.55	0.94
T-PPMP	PF3D7_1001600	=	0.10	0.91
T-PPMP	PF3D7_1019400	=	0.75	0.99
T-PPMP	PF3D7_1311900	=	0.44	0.96
T-PPMP	PF3D7_1035700	=	0.14	0.91
T-PPMP	PF3D7_1103100	=	0.16	0.87
T-PPMP	PF3D7_0812500	=	0.25	0.93
T-PPMP	PF3D7_1216100	=	10.35	0.93
T-PPMP	PF3D7_0206100	=	11.43	0.89
T-PPMP	PF3D7_1352500	=	1.52	0.92
T-PPMP	PF3D7_1226900	=	4.20	0.89
T-PPMP	PF3D7_1368800	=	0.49	0.91
T-PPMP	PF3D7_1432400	=	1.82	0.86
T-PPMP	PF3D7_1343900	=	17.71	0.86
T-PPMP	PF3D7_1328800	=	9.88	0.87
T-PPMP	PF3D7_1138700	=	0.05	0.76
T-PPMP	PF3D7_1327100	=	0.13	0.71
T-PPMP	PF3D7_1136600	=	5.66	0.90
T-PPMP	PF3D7_0902200	=	0.00	0.69
T-PPMP	PF3D7_0727100	=	0.02	0.90
T-PPMP	PF3D7_0105500	=	0.03	0.76
T-PPMP	PF3D7_1126100	=	0.02	0.74
T-PPMP	PF3D7_1343700	=	0.01	0.73

T-PPMP	PF3D7_1330500	=	1.09	0.95
T-PPMP	PF3D7_1343000	=	4.20	0.94
T-PPMP	PF3D7_1302000	=	1.76	0.97
T-PPMP	PF3D7_1107900	=	0.94	0.99
T-PPMP	PF3D7_0606000	=	1.09	0.97
T-PPMP	PF3D7_0614500	=	1.04	0.98
T-PPMP	PF3D7_0203700	=	0.87	0.90
T-PPMP	PF3D7_0601700	=	0.97	0.97
T-PPMP	PF3D7_1001900	=	1.79	0.94
T-PPMP	PF3D7_1409500	=	0.65	0.98
T-PPMP	PF3D7_0916700	=	0.70	0.98
T-PPMP	PF3D7_0402000	=	0.56	0.96
T-PPMP	PF3D7_0102600	=	2.34	0.98
T-PPMP	PF3D7_1433400	=	1.69	0.92
T-PPMP	PF3D7_0322000	=	1.06	0.97
T-PPMP	PF3D7_0623900	=	1.26	0.98
T-PPMP	PF3D7_1013500	=	1.22	0.99

CQ	PF3D7_0811300	=	0.74	0.93
CQ	PF3D7_1305500	=	1.07	0.98
CQ	PF3D7_0615900	=	0.21	0.75
CQ	PF3D7_1122900	=	0.64	0.97
CQ	PF3D7_1141900	=	0.56	0.99
CQ	PF3D7_1475600	=	0.59	0.96
CQ	PF3D7_0211100	=	0.53	0.99
CQ	PF3D7_0203000	=	0.55	0.99
CQ	PF3D7_0404600	=	1.00	0.97
CQ	PF3D7_0416500	=	0.83	0.95
CQ	PF3D7_1018400	=	0.93	0.97
CQ	PF3D7_0511500	=	30.77	0.96
CQ	PF3D7_1360200	=	0.88	0.96
CQ	PF3D7_0611800	=	1.31	0.99
CQ	PF3D7_0619900	=	1.24	0.91
CQ	PF3D7_0521900	=	1.69	0.97
CQ	PF3D7_1219300	=	1.14	1.00
CQ	PF3D7_1133700	=	1.25	0.96
CQ	PF3D7_0808700	=	4.11	1.00
CQ	PF3D7_1136000	=	0.44	0.71
CQ	PF3D7_0931000	=	2.45	0.94
CQ	PF3D7_0830900	=	1.96	0.91
CQ	PF3D7_1231800	=	3.37	0.87
CQ	PF3D7_1035800	=	7.21	0.88
CQ	PF3D7_1444100	=	16.02	1.00
CQ	PF3D7_1138900	=	1.00	0.99
CQ	PF3D7_1441800	=	0.98	0.98
CQ	PF3D7_1345800	=	0.69	0.99
CQ	PF3D7_1432400	=	0.64	0.98
CQ	PF3D7_0819800	=	0.80	0.94
CQ	PF3D7_1207800	=	1.03	1.00
CQ	PF3D7_1143500	=	16.76	1.00

CQ	PF3D7_1001600	=	0.53	0.97
CQ	PF3D7_1019400	=	0.28	0.98
CQ	PF3D7_1311900	=	0.81	0.99
CQ	PF3D7_1035700	=	0.68	0.99
CQ	PF3D7_1103100	=	0.82	0.97
CQ	PF3D7_0812500	=	0.19	0.95
CQ	PF3D7_1216100	=	7.17	0.97
CQ	PF3D7_0206100	=	0.19	0.97
CQ	PF3D7_1352500	=	0.45	0.99
CQ	PF3D7_1226900	=	0.61	0.99
CQ	PF3D7_1368800	=	0.54	0.97
CQ	PF3D7_1432400	=	1.08	0.99
CQ	PF3D7_1343900	=	1.00	0.91
CQ	PF3D7_1328800	=	0.18	0.90
CQ	PF3D7_1138700	=	0.83	0.93
CQ	PF3D7_1327100	=	1.00	0.94
CQ	PF3D7_1136600	=	0.83	0.91
CQ	PF3D7_0902200	=	1.00	0.87
CQ	PF3D7_0727100	=	0.67	1.00
CQ	PF3D7_0105500	=	0.83	0.91
CQ	PF3D7_1126100	=	0.83	0.93
CQ	PF3D7_1343700	=	0.83	0.94
CQ	PF3D7_1330500	=	0.88	0.99
CQ	PF3D7_1343000	=	2.59	0.99
CQ	PF3D7_1302000	=	1.91	0.99
CQ	PF3D7_1107900	=	2.61	1.00
CQ	PF3D7_0606000	=	2.59	0.99
CQ	PF3D7_0614500	=	0.95	0.97
CQ	PF3D7_0203700	=	2.59	1.00
CQ	PF3D7_0601700	=	2.00	0.99
CQ	PF3D7_1001900	=	1.01	0.98
CQ	PF3D7_1409500	=	1.01	0.99
CQ	PF3D7_0916700	=	0.31	0.78
CQ	PF3D7_0402000	=	0.55	0.98
CQ	PF3D7_0102600	=	0.56	0.93
CQ	PF3D7_1433400	=	1.00	0.99
CQ	PF3D7_0322000	=	1.05	0.99
CQ	PF3D7_0623900	=	0.98	0.99
CQ	PF3D7_1013500	=	0.08	0.99

PQ	PF3D7_0811300	=	0.38	0.97
PQ	PF3D7_1305500	=	0.38	0.99
PQ	PF3D7_0615900	=	0.62	0.96
PQ	PF3D7_1122900	=	0.31	0.98
PQ	PF3D7_1141900	=	1.22	1.00
PQ	PF3D7_1475600	=	23.54	0.93
PQ	PF3D7_0211100	=	0.36	1.00
PQ	PF3D7_0203000	=	1.06	0.98
PQ	PF3D7_0404600	=	0.08	0.98
PQ	PF3D7_0416500	=	1.39	0.99

PQ	PF3D7_1018400	=	1.17	0.99
PQ	PF3D7_0511500	=	0.89	0.98
PQ	PF3D7_1360200	=	1.11	0.99
PQ	PF3D7_0611800	=	1.27	0.98
PQ	PF3D7_0619900	=	0.26	0.97
PQ	PF3D7_0521900	=	1.36	0.96
PQ	PF3D7_1219300	=	1.31	0.99
PQ	PF3D7_1133700	=	0.93	0.89
PQ	PF3D7_0808700	=	3.18	0.98
PQ	PF3D7_1136000	=	1.65	0.95
PQ	PF3D7_0931000	=	1.79	0.93
PQ	PF3D7_0830900	=	0.47	0.96
PQ	PF3D7_1231800	=	1.96	0.97
PQ	PF3D7_1035800	=	2.14	0.92
PQ	PF3D7_1444100	=	2.59	0.98
PQ	PF3D7_1138900	=	0.49	0.84
PQ	PF3D7_1441800	=	0.81	1.00
PQ	PF3D7_1345800	=	1.06	0.99
PQ	PF3D7_1432400	=	0.71	0.99
PQ	PF3D7_0819800	=	0.87	1.00
PQ	PF3D7_1207800	=	0.76	1.00
PQ	PF3D7_1143500	=	0.67	1.00
PQ	PF3D7_1001600	=	0.68	1.00
PQ	PF3D7_1019400	=	0.19	0.97
PQ	PF3D7_1311900	=	0.45	0.97
PQ	PF3D7_1035700	=	0.74	0.98
PQ	PF3D7_1103100	=	1.60	0.99
PQ	PF3D7_0812500	=	1.67	0.96
PQ	PF3D7_1216100	=	0.49	0.99
PQ	PF3D7_0206100	=	0.49	0.98
PQ	PF3D7_1352500	=	0.81	0.99
PQ	PF3D7_1226900	=	1.25	0.99
PQ	PF3D7_1368800	=	0.62	0.99
PQ	PF3D7_1432400	=	1.65	0.98
PQ	PF3D7_1343900	=	1.00	0.94
PQ	PF3D7_1328800	=	0.50	0.99
PQ	PF3D7_1138700	=	0.25	0.99
PQ	PF3D7_1327100	=	0.24	0.99
PQ	PF3D7_1136600	=	0.24	0.99
PQ	PF3D7_0902200	=	0.16	0.99
PQ	PF3D7_0727100	=	0.05	0.99
PQ	PF3D7_0105500	=	0.22	0.99
PQ	PF3D7_1126100	=	0.19	0.99
PQ	PF3D7_1343700	=	0.21	1.00
PQ	PF3D7_1330500	=	0.91	0.98
PQ	PF3D7_1343000	=	2.18	0.99
PQ	PF3D7_1302000	=	1.56	0.99
PQ	PF3D7_1107900	=	0.84	0.98
PQ	PF3D7_0606000	=	0.70	0.99
PQ	PF3D7_0614500	=	0.83	0.99
PQ	PF3D7_0203700	=	1.00	0.99
PQ	PF3D7_0601700	=	0.91	0.99

PQ	PF3D7_1001900	=	0.67	0.99
PQ	PF3D7_1409500	=	0.59	0.99
PQ	PF3D7_0916700	=	0.19	0.88
PQ	PF3D7_0402000	=	0.25	1.00
PQ	PF3D7_0102600	=	1.39	0.96
PQ	PF3D7_1433400	=	1.11	0.97
PQ	PF3D7_0322000	=	1.44	0.97
PQ	PF3D7_0623900	=	1.31	0.99
PQ	PF3D7_1013500	=	0.09	0.97

E-64	PF3D7_0811300	=	0.03	0.99
E-64	PF3D7_1305500	=	1.29	0.98
E-64	PF3D7_0615900	=	0.42	0.91
E-64	PF3D7_1122900	=	0.91	0.95
E-64	PF3D7_1141900	=	0.84	0.89
E-64	PF3D7_1475600	=	0.47	0.96
E-64	PF3D7_0211100	=	0.53	0.96
E-64	PF3D7_0203000	=	0.49	0.93
E-64	PF3D7_0404600	=	1.95	0.98
E-64	PF3D7_0416500	=	1.21	0.97
E-64	PF3D7_1018400	=	2.57	0.96
E-64	PF3D7_0511500	=	1.82	0.99
E-64	PF3D7_1360200	=	2.31	0.96
E-64	PF3D7_0611800	=	1.52	0.95
E-64	PF3D7_0619900	=	1.24	0.97
E-64	PF3D7_0521900	=	2.30	0.96
E-64	PF3D7_1219300	=	3.80	0.97
E-64	PF3D7_1133700	=	6.27	0.95
E-64	PF3D7_0808700	=	3.80	0.97
E-64	PF3D7_1136000	=	4.48	0.99
E-64	PF3D7_0931000	=	3.95	0.93
E-64	PF3D7_0830900	=	1.32	0.88
E-64	PF3D7_1231800	=	5.64	0.89
E-64	PF3D7_1035800	=	4.37	0.93
E-64	PF3D7_1444100	=	1.58	0.92
E-64	PF3D7_1138900	=	2.69	0.90
E-64	PF3D7_1441800	=	1.07	0.97
E-64	PF3D7_1345800	=	0.46	0.88
E-64	PF3D7_1432400	=	2.08	0.96
E-64	PF3D7_0819800	=	0.89	0.95
E-64	PF3D7_1207800	=	0.11	0.91
E-64	PF3D7_1143500	=	0.89	0.93
E-64	PF3D7_1001600	=	0.64	0.89
E-64	PF3D7_1019400	=	1.00	0.97
E-64	PF3D7_1311900	=	1.21	0.98
E-64	PF3D7_1035700	=	1.37	0.97
E-64	PF3D7_1103100	=	1.95	0.99
E-64	PF3D7_0812500	=	1.17	0.99
E-64	PF3D7_1216100	=	1.28	0.92
E-64	PF3D7_0206100	=	0.85	0.98

E-64	PF3D7_1352500	=	0.84	0.90
E-64	PF3D7_1226900	=	1.10	0.96
E-64	PF3D7_1368800	=	0.65	0.98
E-64	PF3D7_1432400	=	1.47	0.98
E-64	PF3D7_1343900	=	1.03	0.95
E-64	PF3D7_1328800	=	0.44	0.87
E-64	PF3D7_1138700	=	0.27	0.88
E-64	PF3D7_1327100	=	0.19	0.86
E-64	PF3D7_1136600	=	0.21	0.91
E-64	PF3D7_0902200	=	0.14	0.91
E-64	PF3D7_0727100	=	0.17	0.88
E-64	PF3D7_0105500	=	0.63	0.93
E-64	PF3D7_1126100	=	0.13	0.91
E-64	PF3D7_1343700	=	0.14	0.90
E-64	PF3D7_1330500	=	1.14	0.98
E-64	PF3D7_1343000	=	3.98	0.96
E-64	PF3D7_1302000	=	1.61	0.97
E-64	PF3D7_1107900	=	1.16	0.96
E-64	PF3D7_0606000	=	1.55	0.98
E-64	PF3D7_0614500	=	1.16	0.97
E-64	PF3D7_0203700	=	0.73	0.96
E-64	PF3D7_0601700	=	1.48	0.97
E-64	PF3D7_1001900	=	1.94	0.87
E-64	PF3D7_1409500	=	1.04	0.90
E-64	PF3D7_0916700	=	0.59	0.90
E-64	PF3D7_0402000	=	0.41	0.89
E-64	PF3D7_0102600	=	3.29	0.91
E-64	PF3D7_1433400	=	5.64	0.89
E-64	PF3D7_0322000	=	1.18	0.90
E-64	PF3D7_0623900	=	1.52	0.89
E-64	PF3D7_1013500	=	13.79	0.92

PEP-A	PF3D7_0811300	=	0.95	0.97
PEP-A	PF3D7_1305500	=	0.98	0.98
PEP-A	PF3D7_0615900	=	0.93	0.93
PEP-A	PF3D7_1122900	=	0.62	0.97
PEP-A	PF3D7_1141900	=	0.54	0.91
PEP-A	PF3D7_1475600	=	4.07	0.83
PEP-A	PF3D7_0211100	=	0.64	0.96
PEP-A	PF3D7_0203000	=	0.20	0.92
PEP-A	PF3D7_0404600	=	1.86	0.94
PEP-A	PF3D7_0416500	=	0.83	0.97
PEP-A	PF3D7_1018400	=	1.01	1.00
PEP-A	PF3D7_0511500	=	1.12	0.99
PEP-A	PF3D7_1360200	=	0.83	0.99
PEP-A	PF3D7_0611800	=	0.82	0.98
PEP-A	PF3D7_0619900	=	0.15	0.95
PEP-A	PF3D7_0521900	=	1.10	0.97
PEP-A	PF3D7_1219300	=	0.95	0.99
PEP-A	PF3D7_1133700	=	1.34	0.92

PEP-A	PF3D7_0808700	=	2.51	1.00
PEP-A	PF3D7_1136000	=	1.68	0.99
PEP-A	PF3D7_0931000	=	2.21	0.97
PEP-A	PF3D7_0830900	=	1.23	0.97
PEP-A	PF3D7_1231800	=	107.02	0.96
PEP-A	PF3D7_1035800	=	3.69	0.97
PEP-A	PF3D7_1444100	=	3.62	0.99
PEP-A	PF3D7_1138900	=	1.71	0.96
PEP-A	PF3D7_1441800	=	0.53	0.99
PEP-A	PF3D7_1345800	=	0.46	1.00
PEP-A	PF3D7_1432400	=	1.32	1.00
PEP-A	PF3D7_0819800	=	1.15	0.99
PEP-A	PF3D7_1207800	=	0.81	1.00
PEP-A	PF3D7_1143500	=	0.98	1.00
PEP-A	PF3D7_1001600	=	0.38	1.00
PEP-A	PF3D7_1019400	=	0.16	0.92
PEP-A	PF3D7_1311900	=	1.08	0.99
PEP-A	PF3D7_1035700	=	0.90	1.00
PEP-A	PF3D7_1103100	=	1.52	0.96
PEP-A	PF3D7_0812500	=	1.20	0.99
PEP-A	PF3D7_1216100	=	1.09	0.96
PEP-A	PF3D7_0206100	=	0.37	0.96
PEP-A	PF3D7_1352500	=	7.16	0.99
PEP-A	PF3D7_1226900	=	0.95	0.93
PEP-A	PF3D7_1368800	=	0.45	0.99
PEP-A	PF3D7_1432400	=	1.19	0.93
PEP-A	PF3D7_1343900	=	0.10	0.93
PEP-A	PF3D7_1328800	=	0.29	0.89
PEP-A	PF3D7_1138700	=	0.75	0.96
PEP-A	PF3D7_1327100	=	0.45	0.98
PEP-A	PF3D7_1136600	=	0.58	0.99
PEP-A	PF3D7_0902200	=	0.37	0.98
PEP-A	PF3D7_0727100	=	0.11	0.99
PEP-A	PF3D7_0105500	=	0.46	1.00
PEP-A	PF3D7_1126100	=	0.13	0.94
PEP-A	PF3D7_1343700	=	0.24	0.99
PEP-A	PF3D7_1330500	=	1.67	0.99
PEP-A	PF3D7_1343000	=	2.57	0.99
PEP-A	PF3D7_1302000	=	2.00	0.99
PEP-A	PF3D7_1107900	=	1.68	1.00
PEP-A	PF3D7_0606000	=	1.73	0.98
PEP-A	PF3D7_0614500	=	0.87	0.97
PEP-A	PF3D7_0203700	=	1.53	0.98
PEP-A	PF3D7_0601700	=	1.42	0.98
PEP-A	PF3D7_1001900	=	1.38	1.00
PEP-A	PF3D7_1409500	=	1.07	0.99
PEP-A	PF3D7_0916700	=	0.37	0.95
PEP-A	PF3D7_0402000	=	0.47	1.00
PEP-A	PF3D7_0102600	=	1.34	0.95
PEP-A	PF3D7_1433400	=	0.60	0.97
PEP-A	PF3D7_0322000	=	1.37	0.98
PEP-A	PF3D7_0623900	=	1.08	0.98

PEP-A	PF3D7_1013500	=	0.01	0.68
SF	PF3D7_0811300	=	0.71	0.95
SF	PF3D7_1305500	=	0.35	0.99
SF	PF3D7_0615900	=	0.15	0.86
SF	PF3D7_1122900	=	0.36	0.98
SF	PF3D7_1141900	=	1.40	0.97
SF	PF3D7_1475600	=	0.25	0.98
SF	PF3D7_0211100	=	0.96	0.96
SF	PF3D7_0203000	=	1.00	1.00
SF	PF3D7_0404600	=	1.00	0.86
SF	PF3D7_0416500	=	1.34	0.96
SF	PF3D7_1018400	=	1.66	0.99
SF	PF3D7_0511500	=	0.79	0.99
SF	PF3D7_1360200	=	1.18	1.00
SF	PF3D7_0611800	=	1.03	0.98
SF	PF3D7_0619900	=	0.87	0.96
SF	PF3D7_0521900	=	0.98	0.97
SF	PF3D7_1219300	=	1.16	0.96
SF	PF3D7_1133700	=	1.32	0.98
SF	PF3D7_0808700	=	2.22	0.98
SF	PF3D7_1136000	=	1.54	0.99
SF	PF3D7_0931000	=	1.79	0.96
SF	PF3D7_0830900	=	1.20	0.99
SF	PF3D7_1231800	=	1.97	0.99
SF	PF3D7_1035800	=	1.78	0.96
SF	PF3D7_1444100	=	1.85	0.98
SF	PF3D7_1138900	=	1.45	0.93
SF	PF3D7_1441800	=	1.16	0.99
SF	PF3D7_1345800	=	1.20	0.99
SF	PF3D7_1432400	=	1.44	0.98
SF	PF3D7_0819800	=	1.00	0.98
SF	PF3D7_1207800	=	0.98	0.99
SF	PF3D7_1143500	=	0.39	0.99
SF	PF3D7_1001600	=	0.90	0.99
SF	PF3D7_1019400	=	0.67	0.98
SF	PF3D7_1311900	=	1.38	0.93
SF	PF3D7_1035700	=	0.95	0.99
SF	PF3D7_1103100	=	2.00	0.99
SF	PF3D7_0812500	=	3.74	0.99
SF	PF3D7_1216100	=	0.92	0.99
SF	PF3D7_0206100	=	0.91	0.97
SF	PF3D7_1352500	=	1.13	0.99
SF	PF3D7_1226900	=	2.07	0.99
SF	PF3D7_1368800	=	0.69	0.98
SF	PF3D7_1432400	=	1.36	0.97
SF	PF3D7_1343900	=	0.37	0.98
SF	PF3D7_1328800	=	0.82	0.97
SF	PF3D7_1138700	=	0.38	0.97
SF	PF3D7_1327100	=	0.37	0.97
SF	PF3D7_1136600	=	0.39	0.98

SF	PF3D7_0902200	=	0.41	0.99
SF	PF3D7_0727100	=	0.14	0.88
SF	PF3D7_0105500	=	0.64	0.97
SF	PF3D7_1126100	=	0.37	0.95
SF	PF3D7_1343700	=	0.21	0.92
SF	PF3D7_1330500	=	1.27	0.99
SF	PF3D7_1343000	=	1.73	0.99
SF	PF3D7_1302000	=	1.39	0.98
SF	PF3D7_1107900	=	1.21	0.99
SF	PF3D7_0606000	=	1.82	0.99
SF	PF3D7_0614500	=	0.98	0.99
SF	PF3D7_0203700	=	1.05	1.00
SF	PF3D7_0601700	=	1.65	0.98
SF	PF3D7_1001900	=	0.80	0.96
SF	PF3D7_1409500	=	0.55	0.99
SF	PF3D7_0916700	=	0.30	0.94
SF	PF3D7_0402000	=	0.75	0.99
SF	PF3D7_0102600	=	1.36	0.97
SF	PF3D7_1433400	=	1.37	0.98
SF	PF3D7_0322000	=	1.38	0.99
SF	PF3D7_0623900	=	1.17	0.98
SF	PF3D7_1013500	=	0.32	0.99

CYTD	PF3D7_0811300	=	1.41	0.94
CYTD	PF3D7_1305500	=	0.68	0.96
CYTD	PF3D7_0615900	=	0.98	0.94
CYTD	PF3D7_1122900	=	0.55	0.49
CYTD	PF3D7_1141900	=	2.71	0.97
CYTD	PF3D7_1475600	=	1.37	0.93
CYTD	PF3D7_0211100	=	2.53	0.94
CYTD	PF3D7_0203000	=	1.00	0.95
CYTD	PF3D7_0404600	=	1.66	0.95
CYTD	PF3D7_0416500	=	0.55	0.96
CYTD	PF3D7_1018400	=	0.40	0.92
CYTD	PF3D7_0511500	=	1.15	0.98
CYTD	PF3D7_1360200	=	0.65	0.88
CYTD	PF3D7_0611800	=	0.60	0.96
CYTD	PF3D7_0619900	=	0.71	0.99
CYTD	PF3D7_0521900	=	0.88	0.91
CYTD	PF3D7_1219300	=	0.46	0.96
CYTD	PF3D7_1133700	=	1.89	0.91
CYTD	PF3D7_0808700	=	0.61	0.95
CYTD	PF3D7_1136000	=	1.03	0.93
CYTD	PF3D7_0931000	=	2.09	0.96
CYTD	PF3D7_0830900	=	1.31	0.92
CYTD	PF3D7_1231800	=	4.54	0.95
CYTD	PF3D7_1035800	=	2.68	0.97
CYTD	PF3D7_1444100	=	1.33	0.94
CYTD	PF3D7_1138900	=	8.72	0.96
CYTD	PF3D7_1441800	=	0.21	0.97

CYTD	PF3D7_1345800	=	0.28	0.98
CYTD	PF3D7_1432400	=	1.70	0.98
CYTD	PF3D7_0819800	=	0.27	0.98
CYTD	PF3D7_1207800	=	0.41	0.97
CYTD	PF3D7_1143500	=	0.16	0.97
CYTD	PF3D7_1001600	=	0.17	0.97
CYTD	PF3D7_1019400	=	7.50	1.00
CYTD	PF3D7_1311900	=	0.14	0.97
CYTD	PF3D7_1035700	=	0.24	0.97
CYTD	PF3D7_1103100	=	0.07	0.96
CYTD	PF3D7_0812500	=	0.19	0.96
CYTD	PF3D7_1216100	=	0.06	0.95
CYTD	PF3D7_0206100	=	0.67	0.95
CYTD	PF3D7_1352500	=	0.09	0.85
CYTD	PF3D7_1226900	=	0.01	0.95
CYTD	PF3D7_1368800	=	0.58	0.96
CYTD	PF3D7_1432400	=	0.12	0.98
CYTD	PF3D7_1343900	=	0.10	0.98
CYTD	PF3D7_1328800	=	0.70	0.87
CYTD	PF3D7_1138700	=	0.10	0.97
CYTD	PF3D7_1327100	=	0.04	0.97
CYTD	PF3D7_1136600	=	0.06	0.96
CYTD	PF3D7_0902200	=	0.39	0.97
CYTD	PF3D7_0727100	=	0.77	0.98
CYTD	PF3D7_0105500	=	0.08	0.97
CYTD	PF3D7_1126100	=	0.08	0.96
CYTD	PF3D7_1343700	=	0.08	0.97
CYTD	PF3D7_1330500	=	3.39	0.97
CYTD	PF3D7_1343000	=	0.67	0.96
CYTD	PF3D7_1302000	=	1.33	0.96
CYTD	PF3D7_1107900	=	0.39	0.91
CYTD	PF3D7_0606000	=	2.17	0.96
CYTD	PF3D7_0614500	=	1.94	0.95
CYTD	PF3D7_0203700	=	1.89	0.92
CYTD	PF3D7_0601700	=	2.44	0.96
CYTD	PF3D7_1001900	=	0.77	0.98
CYTD	PF3D7_1409500	=	0.74	0.98
CYTD	PF3D7_0916700	=	1.00	0.98
CYTD	PF3D7_0402000	=	1.62	0.96
CYTD	PF3D7_0102600	=	1.15	0.96
CYTD	PF3D7_1433400	=	1.26	0.97
CYTD	PF3D7_0322000	=	2.26	0.97
CYTD	PF3D7_0623900	=	1.19	0.99
CYTD	PF3D7_1013500	=	1.92	0.99

BZD	PF3D7_0811300	=	0.34	0.96
BZD	PF3D7_1305500	=	0.55	0.99
BZD	PF3D7_0615900	=	0.60	0.98
BZD	PF3D7_1122900	=	0.47	0.94
BZD	PF3D7_1141900	=	0.86	0.98
BZD	PF3D7_1475600	=	0.39	0.99

BZD	PF3D7_0211100	>	42325 uM	
BZD	PF3D7_0203000	=	0.55	0.99
BZD	PF3D7_0404600	=	1.00	0.92
BZD	PF3D7_0416500	=	1.68	0.96
BZD	PF3D7_1018400	=	1.91	0.99
BZD	PF3D7_0511500	=	2.16	0.96
BZD	PF3D7_1360200	=	1.59	0.95
BZD	PF3D7_0611800	=	1.61	0.99
BZD	PF3D7_0619900	=	1.70	0.98
BZD	PF3D7_0521900	=	1.34	0.96
BZD	PF3D7_1219300	=	2.13	0.99
BZD	PF3D7_1133700	=	0.64	0.96
BZD	PF3D7_0808700	=	1.56	0.96
BZD	PF3D7_1136000	=	1.00	0.94
BZD	PF3D7_0931000	=	1.57	0.96
BZD	PF3D7_0830900	=	0.78	0.93
BZD	PF3D7_1231800	=	3.23	0.96
BZD	PF3D7_1035800	=	1.94	0.97
BZD	PF3D7_1444100	=	1.82	0.92
BZD	PF3D7_1138900	=	1.17	0.97
BZD	PF3D7_1441800	=	0.83	0.99
BZD	PF3D7_1345800	=	0.83	0.99
BZD	PF3D7_1432400	=	1.28	1.00
BZD	PF3D7_0819800	=	1.12	0.97
BZD	PF3D7_1207800	=	1.03	0.99
BZD	PF3D7_1143500	=	0.82	1.00
BZD	PF3D7_1001600	=	0.71	0.99
BZD	PF3D7_1019400	=	0.39	0.98
BZD	PF3D7_1311900	=	0.68	0.99
BZD	PF3D7_1035700	=	0.64	0.99
BZD	PF3D7_1103100	=	1.68	0.97
BZD	PF3D7_0812500	=	0.36	1.00
BZD	PF3D7_1216100	=	1.25	0.97
BZD	PF3D7_0206100	=	0.71	0.99
BZD	PF3D7_1352500	=	0.88	0.96
BZD	PF3D7_1226900	=	1.28	0.98
BZD	PF3D7_1368800	=	0.56	0.99
BZD	PF3D7_1432400	=	1.65	0.96
BZD	PF3D7_1343900	=	0.83	0.93
BZD	PF3D7_1328800	=	0.25	0.94
BZD	PF3D7_1138700	=	0.22	0.99
BZD	PF3D7_1327100	=	0.41	0.99
BZD	PF3D7_1136600	=	0.26	0.99
BZD	PF3D7_0902200	=	0.36	1.00
BZD	PF3D7_0727100	=	0.21	1.00
BZD	PF3D7_0105500	=	1.00	1.00
BZD	PF3D7_1126100	=	0.08	0.99
BZD	PF3D7_1343700	=	0.10	0.99
BZD	PF3D7_1330500	=	1.64	0.99
BZD	PF3D7_1343000	=	2.68	0.99
BZD	PF3D7_1302000	=	2.04	1.00
BZD	PF3D7_1107900	=	1.83	0.99

BZD	PF3D7_0606000	=	1.83	0.99
BZD	PF3D7_0614500	=	0.74	0.99
BZD	PF3D7_0203700	=	1.09	1.00
BZD	PF3D7_0601700	=	1.51	0.99
BZD	PF3D7_1001900	=	1.15	0.98
BZD	PF3D7_1409500	=	1.54	0.98
BZD	PF3D7_0916700	=	1.66	0.94
BZD	PF3D7_0402000	=	0.93	1.00
BZD	PF3D7_0102600	=	1.34	0.98
BZD	PF3D7_1433400	=	1.04	1.00
BZD	PF3D7_0322000	=	1.17	0.93
BZD	PF3D7_0623900	=	0.88	0.99
BZD	PF3D7_1013500	=	0.27	1.00
DNTA	PF3D7_0811300	=	0.72	0.95
DNTA	PF3D7_1305500	=	1.43	0.99
DNTA	PF3D7_0615900	=	4.12	0.97
DNTA	PF3D7_1122900	=	1.16	0.97
DNTA	PF3D7_1141900	=	0.92	0.99
DNTA	PF3D7_1475600	=	1.42	0.99
DNTA	PF3D7_0211100	=	0.90	0.91
DNTA	PF3D7_0203000	=	1.02	0.98
DNTA	PF3D7_0404600	=	1.02	0.91
DNTA	PF3D7_0416500	=	1.57	0.94
DNTA	PF3D7_1018400	=	1.56	0.97
DNTA	PF3D7_0511500	=	2.20	0.98
DNTA	PF3D7_1360200	=	1.49	0.90
DNTA	PF3D7_0611800	=	1.39	0.96
DNTA	PF3D7_0619900	=	1.61	0.98
DNTA	PF3D7_0521900	=	2.74	1.00
DNTA	PF3D7_1219300	=	1.78	0.99
DNTA	PF3D7_1133700	=	0.63	0.94
DNTA	PF3D7_0808700	=	0.45	0.96
DNTA	PF3D7_1136000	=	0.65	0.94
DNTA	PF3D7_0931000	=	1.79	0.95
DNTA	PF3D7_0830900	=	2.31	0.96
DNTA	PF3D7_1231800	=	2.26	0.98
DNTA	PF3D7_1035800	=	1.43	0.93
DNTA	PF3D7_1444100	=	0.55	0.89
DNTA	PF3D7_1138900	=	2.11	0.91
DNTA	PF3D7_1441800	=	0.45	0.99
DNTA	PF3D7_1345800	=	0.71	0.99
DNTA	PF3D7_1432400	=	0.71	0.97
DNTA	PF3D7_0819800	=	0.56	0.99
DNTA	PF3D7_1207800	=	0.79	1.00
DNTA	PF3D7_1143500	=	0.43	0.98
DNTA	PF3D7_1001600	=	0.62	0.99
DNTA	PF3D7_1019400	=	1.02	0.99
DNTA	PF3D7_1311900	=	1.63	0.98
DNTA	PF3D7_1035700	=	0.83	0.98

DNTA	PF3D7_1103100	=	0.79	1.00
DNTA	PF3D7_0812500	=	1.00	0.91
DNTA	PF3D7_1216100	=	0.51	0.97
DNTA	PF3D7_0206100	=	1.37	0.97
DNTA	PF3D7_1352500	=	0.97	0.98
DNTA	PF3D7_1226900	=	0.39	0.97
DNTA	PF3D7_1368800	=	1.34	0.93
DNTA	PF3D7_1432400	=	1.11	0.97
DNTA	PF3D7_1343900	=	0.78	0.95
DNTA	PF3D7_1328800	=	0.77	0.87
DNTA	PF3D7_1138700	=	0.23	0.98
DNTA	PF3D7_1327100	=	0.11	0.98
DNTA	PF3D7_1136600	=	0.18	0.99
DNTA	PF3D7_0902200	=	0.11	0.97
DNTA	PF3D7_0727100	=	0.07	0.98
DNTA	PF3D7_0105500	=	0.05	0.97
DNTA	PF3D7_1126100	=	0.05	0.99
DNTA	PF3D7_1343700	=	0.04	0.98
DNTA	PF3D7_1330500	=	0.82	0.98
DNTA	PF3D7_1343000	=	0.69	0.99
DNTA	PF3D7_1302000	=	1.39	0.98
DNTA	PF3D7_1107900	=	0.28	0.98
DNTA	PF3D7_0606000	=	0.55	1.00
DNTA	PF3D7_0614500	=	0.57	0.99
DNTA	PF3D7_0203700	=	0.43	1.00
DNTA	PF3D7_0601700	=	0.47	0.98
DNTA	PF3D7_1001900	=	1.24	0.99
DNTA	PF3D7_1409500	=	0.79	0.97
DNTA	PF3D7_0916700	=	0.60	0.97
DNTA	PF3D7_0402000	=	1.16	0.99
DNTA	PF3D7_0102600	=	0.81	0.99
DNTA	PF3D7_1433400	=	0.48	0.97
DNTA	PF3D7_0322000	=	1.10	0.99
DNTA	PF3D7_0623900	=	0.88	0.98
DNTA	PF3D7_1013500	=	0.40	0.98

TSA	PF3D7_0811300	=	0.08	0.98
TSA	PF3D7_1305500	=	0.99	0.98
TSA	PF3D7_0615900	=	0.69	0.93
TSA	PF3D7_1122900	=	0.90	0.98
TSA	PF3D7_1141900	=	0.43	0.85
TSA	PF3D7_1475600	=	0.32	0.91
TSA	PF3D7_0211100	=	0.26	0.94
TSA	PF3D7_0203000	=	0.52	0.95
TSA	PF3D7_0404600	=	0.86	1.00
TSA	PF3D7_0416500	=	1.15	0.97
TSA	PF3D7_1018400	=	2.77	0.99
TSA	PF3D7_0511500	=	1.03	0.97
TSA	PF3D7_1360200	=	1.08	0.98
TSA	PF3D7_0611800	=	1.79	0.97

TSA	PF3D7_0619900	=	0.38	0.97
TSA	PF3D7_0521900	=	2.90	1.00
TSA	PF3D7_1219300	=	2.85	0.98
TSA	PF3D7_1133700	=	1.22	0.97
TSA	PF3D7_0808700	=	2.23	0.97
TSA	PF3D7_1136000	=	1.25	0.94
TSA	PF3D7_0931000	=	1.04	0.96
TSA	PF3D7_0830900	=	1.56	0.94
TSA	PF3D7_1231800	=	2.37	0.91
TSA	PF3D7_1035800	=	2.60	0.97
TSA	PF3D7_1444100	=	4.06	0.86
TSA	PF3D7_1138900	=	2.09	0.95
TSA	PF3D7_1441800	=	0.61	0.96
TSA	PF3D7_1345800	=	0.75	0.96
TSA	PF3D7_1432400	=	0.81	0.96
TSA	PF3D7_0819800	=	0.21	0.94
TSA	PF3D7_1207800	=	0.06	0.94
TSA	PF3D7_1143500	=	0.10	0.93
TSA	PF3D7_1001600	=	0.30	0.94
TSA	PF3D7_1019400	=	0.56	0.97
TSA	PF3D7_1311900	=	0.43	0.99
TSA	PF3D7_1035700	=	0.54	0.97
TSA	PF3D7_1103100	=	0.51	0.99
TSA	PF3D7_0812500	=	0.20	0.92
TSA	PF3D7_1216100	=	0.72	0.97
TSA	PF3D7_0206100	=	1.06	0.98
TSA	PF3D7_1352500	=	0.91	0.99
TSA	PF3D7_1226900	=	1.24	0.99
TSA	PF3D7_1368800	=	0.35	0.97
TSA	PF3D7_1432400	=	1.95	0.99
TSA	PF3D7_1343900	=	0.76	0.99
TSA	PF3D7_1328800	=	0.72	0.99
TSA	PF3D7_1138700	=	0.47	0.93
TSA	PF3D7_1327100	=	0.20	0.91
TSA	PF3D7_1136600	=	0.17	0.93
TSA	PF3D7_0902200	=	270842.23	0.10
TSA	PF3D7_0727100	=	0.07	0.86
TSA	PF3D7_0105500	=	0.50	0.94
TSA	PF3D7_1126100	=	0.17	0.89
TSA	PF3D7_1343700	=	0.20	0.87
TSA	PF3D7_1330500	=	1.67	0.98
TSA	PF3D7_1343000	=	4.71	0.99
TSA	PF3D7_1302000	=	4.08	0.99
TSA	PF3D7_1107900	=	1.88	0.97
TSA	PF3D7_0606000	=	3.71	1.00
TSA	PF3D7_0614500	=	2.92	0.99
TSA	PF3D7_0203700	=	2.17	0.99
TSA	PF3D7_0601700	=	2.50	0.99
TSA	PF3D7_1001900	=	1.43	0.89
TSA	PF3D7_1409500	=	0.83	0.97
TSA	PF3D7_0916700	=	0.60	0.96
TSA	PF3D7_0402000	=	0.76	0.99

TSA	PF3D7_0102600	=	2.52	0.94
TSA	PF3D7_1433400	=	1.25	0.98
TSA	PF3D7_0322000	=	0.71	0.95
TSA	PF3D7_0623900	=	0.92	0.95
TSA	PF3D7_1013500	=	0.51	0.99

CUR	PF3D7_0811300	=	0.28	0.93
CUR	PF3D7_1305500	=	0.66	0.93
CUR	PF3D7_0615900	=	0.76	0.94
CUR	PF3D7_1122900	=	0.71	0.58
CUR	PF3D7_1141900	=	3.51	0.96
CUR	PF3D7_1475600	=	0.62	0.90
CUR	PF3D7_0211100	=	4.21	0.94
CUR	PF3D7_0203000	=	4.21	0.80
CUR	PF3D7_0404600	=	0.51	0.91
CUR	PF3D7_0416500	=	3.19	0.95
CUR	PF3D7_1018400	=	4.10	0.92
CUR	PF3D7_0511500	=	1.57	0.87
CUR	PF3D7_1360200	=	0.40	0.94
CUR	PF3D7_0611800	=	1.10	0.97
CUR	PF3D7_0619900	=	1.72	0.99
CUR	PF3D7_0521900	=	3.33	0.97
CUR	PF3D7_1219300	=	3.42	0.92
CUR	PF3D7_1133700	=	392.78	0.90
CUR	PF3D7_0808700	=	9.17	0.91
CUR	PF3D7_1136000	=	1.31	0.90
CUR	PF3D7_0931000	=	7.16	0.96
CUR	PF3D7_0830900	=	12.49	0.93
CUR	PF3D7_1231800	=	11.53	0.96
CUR	PF3D7_1035800	=	2.23	0.97
CUR	PF3D7_1444100	=	8.82	0.92
CUR	PF3D7_1138900	=	2.29	0.95
CUR	PF3D7_1441800	=	1.06	0.96
CUR	PF3D7_1345800	=	0.14	0.95
CUR	PF3D7_1432400	=	0.53	0.99
CUR	PF3D7_0819800	=	0.37	0.99
CUR	PF3D7_1207800	=	0.38	1.00
CUR	PF3D7_1143500	=	0.18	0.98
CUR	PF3D7_1001600	=	0.31	0.99
CUR	PF3D7_1019400	>	2262.7 uM	
CUR	PF3D7_1311900	=	0.76	0.96
CUR	PF3D7_1035700	=	0.37	0.97
CUR	PF3D7_1103100	=	0.03	0.94
CUR	PF3D7_0812500	=	0.10	0.84
CUR	PF3D7_1216100	=	1.32	0.98
CUR	PF3D7_0206100	=	0.56	0.95
CUR	PF3D7_1352500	=	0.70	0.98
CUR	PF3D7_1226900	=	2.19	0.98
CUR	PF3D7_1368800	=	9.38	0.92
CUR	PF3D7_1432400	=	0.48	0.99

CUR	PF3D7_1343900	=	0.43	0.95
CUR	PF3D7_1328800	=	0.99	0.97
CUR	PF3D7_1138700	=	0.34	0.96
CUR	PF3D7_1327100	=	0.17	0.97
CUR	PF3D7_1136600	=	0.28	0.98
CUR	PF3D7_0902200	=	0.85	0.99
CUR	PF3D7_0727100	=	0.28	0.98
CUR	PF3D7_0105500	=	0.17	0.99
CUR	PF3D7_1126100	=	0.34	0.98
CUR	PF3D7_1343700	=	0.18	1.00
CUR	PF3D7_1330500	=	2.75	0.99
CUR	PF3D7_1343000	=	1.47	0.98
CUR	PF3D7_1302000	=	2.92	0.99
CUR	PF3D7_1107900	=	1.54	1.00
CUR	PF3D7_0606000	=	2.24	0.96
CUR	PF3D7_0614500	=	2.46	0.99
CUR	PF3D7_0203700	=	1.63	0.97
CUR	PF3D7_0601700	=	2.43	0.97
CUR	PF3D7_1001900		0.15	0.93
CUR	PF3D7_1409500	=	0.98	0.75
CUR	PF3D7_0916700	=	0.11	0.98
CUR	PF3D7_0402000	=	0.18	0.94
CUR	PF3D7_0102600	=	0.19	0.94
CUR	PF3D7_1433400	=	0.21	0.98
CUR	PF3D7_0322000	=	0.24	0.98
CUR	PF3D7_0623900	=	0.63	0.99
CUR	PF3D7_1013500	=	0.40	0.92
DNR	PF3D7_0811300	=	1.03	0.96
DNR	PF3D7_1305500	=	0.60	0.99
DNR	PF3D7_0615900	=	0.25	0.95
DNR	PF3D7_1122900	=	0.47	0.95
DNR	PF3D7_1141900	=	0.69	0.90
DNR	PF3D7_1475600	=	0.50	0.99
DNR	PF3D7_0211100	=	0.25	0.87
DNR	PF3D7_0203000	=	0.69	0.95
DNR	PF3D7_0404600	=	1.00	0.99
DNR	PF3D7_0416500	=	0.09	0.94
DNR	PF3D7_1018400	=	0.10	0.95
DNR	PF3D7_0511500	=	0.20	0.97
DNR	PF3D7_1360200	=	0.11	0.98
DNR	PF3D7_0611800	=	0.17	0.99
DNR	PF3D7_0619900	=	0.00	0.93
DNR	PF3D7_0521900	=	0.14	0.96
DNR	PF3D7_1219300	=	0.15	0.98
DNR	PF3D7_1133700	=	1.09	0.95
DNR	PF3D7_0808700	=	2.45	0.95
DNR	PF3D7_1136000	=	2.06	0.98
DNR	PF3D7_0931000	=	1.76	0.96
DNR	PF3D7_0830900	=	1.38	0.98

DNR	PF3D7_1231800	=	5.17	0.97
DNR	PF3D7_1035800	=	8.52	0.91
DNR	PF3D7_1444100	=	5.49	0.97
DNR	PF3D7_1138900	=	1.05	0.93
DNR	PF3D7_1441800	=	0.70	0.99
DNR	PF3D7_1345800	=	0.59	1.00
DNR	PF3D7_1432400	=	0.56	1.00
DNR	PF3D7_0819800	=	0.69	1.00
DNR	PF3D7_1207800	=	0.55	1.00
DNR	PF3D7_1143500	=	0.47	1.00
DNR	PF3D7_1001600	=	0.45	0.98
DNR	PF3D7_1019400	=	0.04	0.98
DNR	PF3D7_1311900	=	1.04	1.00
DNR	PF3D7_1035700	=	0.94	1.00
DNR	PF3D7_1103100	=	1.06	0.98
DNR	PF3D7_0812500	=	0.84	0.96
DNR	PF3D7_1216100	=	1.90	1.00
DNR	PF3D7_0206100	=	0.76	0.99
DNR	PF3D7_1352500	=	0.87	1.00
DNR	PF3D7_1226900	=	0.80	1.00
DNR	PF3D7_1368800	=	0.96	0.99
DNR	PF3D7_1432400	=	2.22	0.99
DNR	PF3D7_1343900	=	0.09	0.84
DNR	PF3D7_1328800	=	0.44	0.97
DNR	PF3D7_1138700	=	0.31	0.98
DNR	PF3D7_1327100	=	0.42	0.99
DNR	PF3D7_1136600	=	0.40	0.99
DNR	PF3D7_0902200	=	0.32	0.99
DNR	PF3D7_0727100	=	0.17	1.00
DNR	PF3D7_0105500	=	0.33	0.99
DNR	PF3D7_1126100	=	0.16	0.97
DNR	PF3D7_1343700	=	0.19	0.99
DNR	PF3D7_1330500	=	0.94	0.98
DNR	PF3D7_1343000	=	1.73	1.00
DNR	PF3D7_1302000	=	1.42	0.99
DNR	PF3D7_1107900	=	1.57	1.00
DNR	PF3D7_0606000	=	1.30	0.99
DNR	PF3D7_0614500	=	1.14	0.99
DNR	PF3D7_0203700	=	1.44	1.00
DNR	PF3D7_0601700	=	1.30	0.99
DNR	PF3D7_1001900	=	1.07	0.99
DNR	PF3D7_1409500	=	1.03	1.00
DNR	PF3D7_0916700	=	0.70	0.94
DNR	PF3D7_0402000	=	0.54	0.99
DNR	PF3D7_0102600	=	0.92	0.97
DNR	PF3D7_1433400	=	0.99	0.99
DNR	PF3D7_0322000	=	1.05	0.99
DNR	PF3D7_0623900	=	0.97	0.97
DNR	PF3D7_1013500	=	0.04	0.95

QHS	PF3D7_0811300	=	1.12	0.95
QHS	PF3D7_1305500	=	1.58	0.98
QHS	PF3D7_0615900	=	0.51	0.98
QHS	PF3D7_1122900	=	0.51	0.99
QHS	PF3D7_1141900	=	0.86	0.93
QHS	PF3D7_1475600	=	0.51	0.94
QHS	PF3D7_0211100	=	0.79	0.97
QHS	PF3D7_0203000	=	0.33	0.93
QHS	PF3D7_0404600	=	1.00	0.98
QHS	PF3D7_0416500	=	0.35	0.91
QHS	PF3D7_1018400	=	0.67	0.94
QHS	PF3D7_0511500	=	0.49	0.90
QHS	PF3D7_1360200	=	0.50	0.99
QHS	PF3D7_0611800	=	0.98	0.99
QHS	PF3D7_0619900	=	0.63	0.99
QHS	PF3D7_0521900	=	0.59	0.95
QHS	PF3D7_1219300	=	1.21	0.97
QHS	PF3D7_1133700	=	1.48	0.95
QHS	PF3D7_0808700	=	5.17	0.99
QHS	PF3D7_1136000	=	2.63	0.91
QHS	PF3D7_0931000	=	2.70	0.94
QHS	PF3D7_0830900	=	1.78	0.85
QHS	PF3D7_1231800	=	3.70	0.96
QHS	PF3D7_1035800	=	2.02	0.93
QHS	PF3D7_1444100	=	2.12	0.98
QHS	PF3D7_1138900	=	1.93	0.94
QHS	PF3D7_1441800	=	0.60	0.99
QHS	PF3D7_1345800	=	1.12	0.99
QHS	PF3D7_1432400	=	0.50	0.99
QHS	PF3D7_0819800	=	0.75	0.92
QHS	PF3D7_1207800	=	0.81	0.98
QHS	PF3D7_1143500	=	0.66	0.96
QHS	PF3D7_1001600	=	0.50	0.98
QHS	PF3D7_1019400	=	0.24	0.96
QHS	PF3D7_1311900	=	1.00	0.99
QHS	PF3D7_1035700	=	1.24	0.96
QHS	PF3D7_1103100	=	1.15	0.91
QHS	PF3D7_0812500	=	0.22	0.99
QHS	PF3D7_1216100	=	1.37	0.98
QHS	PF3D7_0206100	=	0.44	0.99
QHS	PF3D7_1352500	=	0.93	0.99
QHS	PF3D7_1226900	=	1.85	0.98
QHS	PF3D7_1368800	=	0.52	0.99
QHS	PF3D7_1432400	=	1.70	0.98
QHS	PF3D7_1343900	=	0.42	0.95
QHS	PF3D7_1328800	=	0.30	0.98
QHS	PF3D7_1138700	=	0.29	0.96
QHS	PF3D7_1327100	=	0.28	0.94
QHS	PF3D7_1136600	=	0.34	0.97
QHS	PF3D7_0902200	=	0.19	0.95
QHS	PF3D7_0727100	=	0.05	0.82
QHS	PF3D7_0105500	=	0.14	0.92

QHS	PF3D7_1126100	=	0.15	0.95
QHS	PF3D7_1343700	=	0.15	0.96
QHS	PF3D7_1330500	=	0.64	0.97
QHS	PF3D7_1343000	=	1.51	0.99
QHS	PF3D7_1302000	=	1.26	0.99
QHS	PF3D7_1107900	=	2.16	0.99
QHS	PF3D7_0606000	=	1.07	0.96
QHS	PF3D7_0614500	=	0.51	0.94
QHS	PF3D7_0203700	=	1.01	0.95
QHS	PF3D7_0601700	=	1.14	0.98
QHS	PF3D7_1001900	=	1.07	0.98
QHS	PF3D7_1409500	=	1.47	0.99
QHS	PF3D7_0916700	=	0.82	0.95
QHS	PF3D7_0402000	=	0.27	1.00
QHS	PF3D7_0102600	=	1.01	0.98
QHS	PF3D7_1433400	=	0.94	1.00
QHS	PF3D7_0322000	=	1.03	0.99
QHS	PF3D7_0623900	=	0.96	1.00
QHS	PF3D7_1013500	=	0.17	0.95

DHA	PF3D7_0811300	=	1.26	0.95
DHA	PF3D7_1305500	=	1.32	0.98
DHA	PF3D7_0615900	=	0.52	0.93
DHA	PF3D7_1122900	=	0.55	0.96
DHA	PF3D7_1141900	=	1.65	0.99
DHA	PF3D7_1475600	=	0.52	0.97
DHA	PF3D7_0211100	=	0.81	0.99
DHA	PF3D7_0203000	=	0.52	0.96
DHA	PF3D7_0404600	=	1.00	0.99
DHA	PF3D7_0416500	=	1.69	0.99
DHA	PF3D7_1018400	=	1.89	0.98
DHA	PF3D7_0511500	=	1.08	0.94
DHA	PF3D7_1360200	=	1.45	0.94
DHA	PF3D7_0611800	=	2.75	0.98
DHA	PF3D7_0619900	=	0.64	0.98
DHA	PF3D7_0521900	=	1.51	0.98
DHA	PF3D7_1219300	=	2.70	0.96
DHA	PF3D7_1133700	=	0.54	0.93
DHA	PF3D7_0808700	=	7.39	0.97
DHA	PF3D7_1136000	=	2.50	0.92
DHA	PF3D7_0931000	=	4.90	0.94
DHA	PF3D7_0830900	=	1.33	0.76
DHA	PF3D7_1231800	=	5.40	0.99
DHA	PF3D7_1035800	=	2.10	0.92
DHA	PF3D7_1444100	=	3.70	0.89
DHA	PF3D7_1138900	=	1.67	0.97
DHA	PF3D7_1441800	=	0.56	0.99
DHA	PF3D7_1345800	=	0.86	0.99
DHA	PF3D7_1432400	=	1.05	0.99

DHA	PF3D7_0819800	=	0.63	0.93
DHA	PF3D7_1207800	=	0.87	0.99
DHA	PF3D7_1143500	=	0.37	0.99
DHA	PF3D7_1001600	=	0.58	0.98
DHA	PF3D7_1019400	=	0.31	0.94
DHA	PF3D7_1311900	=	0.59	0.87
DHA	PF3D7_1035700	=	0.83	0.99
DHA	PF3D7_1103100	=	1.50	0.96
DHA	PF3D7_0812500	=	0.61	0.83
DHA	PF3D7_1216100	=	1.06	0.97
DHA	PF3D7_0206100	=	0.28	0.96
DHA	PF3D7_1352500	=	1.27	0.97
DHA	PF3D7_1226900	=	2.10	0.97
DHA	PF3D7_1368800	=	0.34	0.97
DHA	PF3D7_1432400	=	2.10	0.97
DHA	PF3D7_1343900	=	0.35	0.97
DHA	PF3D7_1328800	=	0.18	0.98
DHA	PF3D7_1138700	=	0.20	0.96
DHA	PF3D7_1327100	=	0.17	0.96
DHA	PF3D7_1136600	=	0.20	0.97
DHA	PF3D7_0902200	=	0.30	0.95
DHA	PF3D7_0727100	=	0.03	0.87
DHA	PF3D7_0105500	=	0.27	0.92
DHA	PF3D7_1126100	=	0.13	0.93
DHA	PF3D7_1343700	=	0.13	0.93
DHA	PF3D7_1330500	=	0.45	0.97
DHA	PF3D7_1343000	=	1.80	0.98
DHA	PF3D7_1302000	=	1.25	0.98
DHA	PF3D7_1107900	=	1.98	0.99
DHA	PF3D7_0606000	=	1.50	0.99
DHA	PF3D7_0614500	=	0.50	0.98
DHA	PF3D7_0203700	=	0.65	0.98
DHA	PF3D7_0601700	=	0.85	0.99
DHA	PF3D7_1001900	=	1.72	0.99
DHA	PF3D7_1409500	=	1.29	0.99
DHA	PF3D7_0916700	=	1.22	0.93
DHA	PF3D7_0402000	=	0.30	0.99
DHA	PF3D7_0102600	=	1.31	0.98
DHA	PF3D7_1433400	=	1.01	0.99
DHA	PF3D7_0322000	=	1.19	0.99
DHA	PF3D7_0623900	=	1.23	0.97
DHA	PF3D7_1013500	=	0.22	0.99

VER	PF3D7_0811300	=	0.76	0.96
VER	PF3D7_1305500	=	0.67	0.99
VER	PF3D7_0615900	=	1.02	0.98
VER	PF3D7_1122900	=	0.41	0.90
VER	PF3D7_1141900	=	1.16	1.00
VER	PF3D7_1475600	=	0.46	0.99

VER	PF3D7_0211100	=	0.97	0.96
VER	PF3D7_0203000	=	1.00	0.99
VER	PF3D7_0404600	=	1.00	0.99
VER	PF3D7_0416500	=	1.20	0.96
VER	PF3D7_1018400	=	1.15	0.93
VER	PF3D7_0511500	=	1.61	0.97
VER	PF3D7_1360200	=	1.09	0.98
VER	PF3D7_0611800	=	0.77	0.95
VER	PF3D7_0619900	=	0.75	0.93
VER	PF3D7_0521900	=	0.94	0.96
VER	PF3D7_1219300	=	1.35	0.95
VER	PF3D7_1133700	=	0.60	0.93
VER	PF3D7_0808700	=	1.36	0.95
VER	PF3D7_1136000	=	0.96	0.95
VER	PF3D7_0931000	=	1.24	0.94
VER	PF3D7_0830900	=	0.24	0.89
VER	PF3D7_1231800	=	1.38	0.95
VER	PF3D7_1035800	=	3.06	0.88
VER	PF3D7_1444100	=	1.37	0.99
VER	PF3D7_1138900	=	0.72	0.91
VER	PF3D7_1441800	=	0.61	0.99
VER	PF3D7_1345800	=	0.77	0.99
VER	PF3D7_1432400	=	1.27	0.99
VER	PF3D7_0819800	=	0.88	0.99
VER	PF3D7_1207800	=	0.84	0.98
VER	PF3D7_1143500	=	0.93	0.98
VER	PF3D7_1001600	=	0.76	1.00
VER	PF3D7_1019400	=	0.41	0.99
VER	PF3D7_1311900	=	0.59	0.97
VER	PF3D7_1035700	=	0.90	0.98
VER	PF3D7_1103100	=	1.40	0.95
VER	PF3D7_0812500	=	0.53	0.93
VER	PF3D7_1216100	=	1.21	0.98
VER	PF3D7_0206100	=	0.88	0.98
VER	PF3D7_1352500	=	0.62	0.99
VER	PF3D7_1226900	=	0.96	0.99
VER	PF3D7_1368800	=	0.54	1.00
VER	PF3D7_1432400	=	1.74	0.97
VER	PF3D7_1343900	=	0.64	0.97
VER	PF3D7_1328800	=	0.48	0.99
VER	PF3D7_1138700	=	0.53	0.99
VER	PF3D7_1327100	=	0.48	0.97
VER	PF3D7_1136600	=	0.28	0.99
VER	PF3D7_0902200	=	0.43	0.97
VER	PF3D7_0727100	=	0.02	0.99
VER	PF3D7_0105500	=	0.30	0.98
VER	PF3D7_1126100	=	0.21	1.00
VER	PF3D7_1343700	=	0.31	1.00
VER	PF3D7_1330500	=	1.42	1.00
VER	PF3D7_1343000	=	1.55	1.00
VER	PF3D7_1302000	=	1.79	0.99
VER	PF3D7_1107900	=	1.28	1.00

VER	PF3D7_0606000	=	1.38	0.99
VER	PF3D7_0614500	=	0.87	1.00
VER	PF3D7_0203700	=	1.34	1.00
VER	PF3D7_0601700	=	1.17	0.99
VER	PF3D7_1001900	=	1.10	1.00
VER	PF3D7_1409500	=	0.98	0.94
VER	PF3D7_0916700	=	0.92	0.99
VER	PF3D7_0402000	=	0.76	0.98
VER	PF3D7_0102600	=	0.54	0.99
VER	PF3D7_1433400	=	0.83	0.98
VER	PF3D7_0322000	=	1.26	0.98
VER	PF3D7_0623900	=	1.12	0.99
VER	PF3D7_1013500	=	0.26	0.92

AQ	PF3D7_0811300	=	0.20	0.89
AQ	PF3D7_1305500	=	0.60	0.99
AQ	PF3D7_0615900	=	0.89	0.98
AQ	PF3D7_1122900	=	0.40	0.96
AQ	PF3D7_1141900	=	0.38	0.96
AQ	PF3D7_1475600	=	0.65	0.99
AQ	PF3D7_0211100	=	1.45	1.00
AQ	PF3D7_0203000	=	0.16	0.98
AQ	PF3D7_0404600	=	0.25	0.85
AQ	PF3D7_0416500	=	0.77	0.83
AQ	PF3D7_1018400	=	0.93	0.90
AQ	PF3D7_0511500	=	1.33	1.00
AQ	PF3D7_1360200	=	0.86	0.99
AQ	PF3D7_0611800	=	1.29	0.98
AQ	PF3D7_0619900	=	0.65	0.98
AQ	PF3D7_0521900	=	1.27	0.99
AQ	PF3D7_1219300	=	0.85	0.93
AQ	PF3D7_1133700	=	1.08	1.00
AQ	PF3D7_0808700	=	1.07	0.99
AQ	PF3D7_1136000	=	1.19	1.00
AQ	PF3D7_0931000	=	1.74	0.99
AQ	PF3D7_0830900	=	2.82	0.95
AQ	PF3D7_1231800	=	2.18	1.00
AQ	PF3D7_1035800	=	2.15	1.00
AQ	PF3D7_1444100	=	1.50	0.99
AQ	PF3D7_1138900	=	1.73	0.97
AQ	PF3D7_1441800	=	0.43	0.94
AQ	PF3D7_1345800	=	1.45	0.99
AQ	PF3D7_1432400	=	0.50	0.98
AQ	PF3D7_0819800	=	0.46	0.99
AQ	PF3D7_1207800	=	1.42	0.99
AQ	PF3D7_1143500	=	0.70	0.96
AQ	PF3D7_1001600	=	0.72	0.99
AQ	PF3D7_1019400	=	0.60	0.90
AQ	PF3D7_1311900	=	0.97	0.91

AQ	PF3D7_1035700	=	1.18	0.98
AQ	PF3D7_1103100	=	1.14	0.99
AQ	PF3D7_0812500	=	1.04	0.97
AQ	PF3D7_1216100	=	2.88	0.90
AQ	PF3D7_0206100	=	24.84	0.89
AQ	PF3D7_1352500	=	30.48	0.99
AQ	PF3D7_1226900	=	1.60	0.91
AQ	PF3D7_1368800	=	26.87	0.95
AQ	PF3D7_1432400	=	1.01	0.88
AQ	PF3D7_1343900	=	0.30	0.89
AQ	PF3D7_1328800	=	29.81	0.82
AQ	PF3D7_1138700	=	0.82	0.97
AQ	PF3D7_1327100	=	0.20	0.96
AQ	PF3D7_1136600	=	0.46	0.98
AQ	PF3D7_0902200	=	0.69	0.91
AQ	PF3D7_0727100	=	0.91	0.97
AQ	PF3D7_0105500	=	0.26	0.94
AQ	PF3D7_1126100	=	0.35	0.99
AQ	PF3D7_1343700	=	0.48	0.96
AQ	PF3D7_1330500	=	0.99	0.99
AQ	PF3D7_1343000	=	1.23	0.97
AQ	PF3D7_1302000	=	1.28	0.97
AQ	PF3D7_1107900	=	1.11	0.96
AQ	PF3D7_0606000	=	1.48	0.96
AQ	PF3D7_0614500	=	2.24	0.99
AQ	PF3D7_0203700	=	1.58	0.96
AQ	PF3D7_0601700	=	1.59	0.96
AQ	PF3D7_1001900	=	2.25	0.99
AQ	PF3D7_1409500	=	1.08	0.97
AQ	PF3D7_0916700	=	1.51	0.99
AQ	PF3D7_0402000	=	1.58	0.98
AQ	PF3D7_0102600	=	0.91	0.99
AQ	PF3D7_1433400	=	1.57	0.99
AQ	PF3D7_0322000	=	2.40	0.99
AQ	PF3D7_0623900	=	1.95	0.98
AQ	PF3D7_1013500	=	0.14	1.00
AS	PF3D7_0811300	=	0.44	0.95
AS	PF3D7_1305500	=	0.67	0.99
AS	PF3D7_0615900	=	1.00	0.99
AS	PF3D7_1122900	=	0.44	0.91
AS	PF3D7_1141900	=	0.56	1.00
AS	PF3D7_1475600	=	0.89	0.99
AS	PF3D7_0211100	=	1.22	0.96
AS	PF3D7_0203000	=	0.91	0.17
AS	PF3D7_0404600	=	1.33	0.99
AS	PF3D7_0416500	=	0.85	0.90
AS	PF3D7_1018400	=	0.46	0.91
AS	PF3D7_0511500	=	0.84	0.94
AS	PF3D7_1360200	=	1.48	0.85
AS	PF3D7_0611800	=	1.33	0.95

AS	PF3D7_0619900	=	0.80	0.98
AS	PF3D7_0521900	=	1.60	0.99
AS	PF3D7_1219300	=	1.21	0.89
AS	PF3D7_1133700	=	0.71	0.95
AS	PF3D7_0808700	=	0.80	1.00
AS	PF3D7_1136000	=	1.27	0.97
AS	PF3D7_0931000	=	3.42	0.97
AS	PF3D7_0830900	=	1.95	0.92
AS	PF3D7_1231800	=	4.88	1.00
AS	PF3D7_1035800	=	2.91	0.98
AS	PF3D7_1444100	=	3.04	0.95
AS	PF3D7_1138900	=	2.10	0.94
AS	PF3D7_1441800	=	0.79	0.82
AS	PF3D7_1345800	=	1.75	0.98
AS	PF3D7_1432400	=	1.46	0.97
AS	PF3D7_0819800	=	0.41	0.93
AS	PF3D7_1207800	=	1.53	0.99
AS	PF3D7_1143500	=	0.75	0.99
AS	PF3D7_1001600	=	0.75	1.00
AS	PF3D7_1019400	=	1.41	0.92
AS	PF3D7_1311900	=	1.03	0.99
AS	PF3D7_1035700	=	0.87	0.99
AS	PF3D7_1103100	=	0.75	0.94
AS	PF3D7_0812500	=	0.73	0.94
AS	PF3D7_1216100	=	0.52	0.86
AS	PF3D7_0206100	=	30.48	0.84
AS	PF3D7_1352500	=	33.90	0.99
AS	PF3D7_1226900	=	0.28	0.84
AS	PF3D7_1368800	=	78.80	0.88
AS	PF3D7_1432400	=	76.16	0.79
AS	PF3D7_1343900	=	10.93	0.85
AS	PF3D7_1328800	=	75.88	0.99
AS	PF3D7_1138700	=	0.93	0.98
AS	PF3D7_1327100	=	0.41	0.98
AS	PF3D7_1136600	=	0.49	0.98
AS	PF3D7_0902200	=	0.85	0.99
AS	PF3D7_0727100	=	1.09	0.95
AS	PF3D7_0105500	=	0.24	0.97
AS	PF3D7_1126100	=	0.42	0.97
AS	PF3D7_1343700	=	0.50	0.95
AS	PF3D7_1330500	=	0.84	0.91
AS	PF3D7_1343000	=	0.90	0.89
AS	PF3D7_1302000	=	0.84	0.88
AS	PF3D7_1107900	=	0.62	0.90
AS	PF3D7_0606000	=	0.77	0.89
AS	PF3D7_0614500	=	1.42	0.89
AS	PF3D7_0203700	=	1.13	0.86
AS	PF3D7_0601700	=	1.27	0.57
AS	PF3D7_1001900	=	1.28	0.99
AS	PF3D7_1409500	=	1.44	0.99
AS	PF3D7_0916700	=	2.01	1.00
AS	PF3D7_0402000	=	1.83	0.99

AS	PF3D7_0102600	=	0.68	0.99
AS	PF3D7_1433400	=	1.57	1.00
AS	PF3D7_0322000	=	1.58	0.99
AS	PF3D7_0623900	=	1.52	0.89
AS	PF3D7_1013500	=	2.78	0.92
AL	PF3D7_0811300	=	0.56	0.97
AL	PF3D7_1305500	=	0.58	0.98
AL	PF3D7_0615900	=	0.48	0.98
AL	PF3D7_1122900	=	0.45	0.96
AL	PF3D7_1141900	=	0.30	1.00
AL	PF3D7_1475600	=	0.53	0.99
AL	PF3D7_0211100	=	0.81	1.00
AL	PF3D7_0203000	=	0.31	0.82
AL	PF3D7_0404600	=	1.13	1.00
AL	PF3D7_0416500	=	0.36	0.87
AL	PF3D7_1018400	=	0.53	0.90
AL	PF3D7_0511500	=	5.00	0.99
AL	PF3D7_1360200	=	2.80	0.96
AL	PF3D7_0611800	=	1.64	1.00
AL	PF3D7_0619900	=	1.14	0.99
AL	PF3D7_0521900	=	1.57	0.96
AL	PF3D7_1219300	=	1.34	0.97
AL	PF3D7_1133700	=	0.92	0.98
AL	PF3D7_0808700	=	0.71	0.99
AL	PF3D7_1136000	=	1.30	0.99
AL	PF3D7_0931000	=	1.60	0.97
AL	PF3D7_0830900	=	2.19	0.94
AL	PF3D7_1231800	=	4.50	0.98
AL	PF3D7_1035800	=	1.50	0.97
AL	PF3D7_1444100	=	2.10	0.93
AL	PF3D7_1138900	=	1.66	0.97
AL	PF3D7_1441800	=	1.03	1.00
AL	PF3D7_1345800	=	3.02	0.98
AL	PF3D7_1432400	=	0.80	0.98
AL	PF3D7_0819800	=	1.04	0.99
AL	PF3D7_1207800	=	2.38	0.99
AL	PF3D7_1143500	=	1.32	0.99
AL	PF3D7_1001600	=	1.20	0.95
AL	PF3D7_1019400	=	1.91	1.00
AL	PF3D7_1311900	=	1.82	0.97
AL	PF3D7_1035700	=	1.16	0.97
AL	PF3D7_1103100	=	1.38	0.93
AL	PF3D7_0812500	=	1.09	1.00
AL	PF3D7_1216100	=	0.45	0.94
AL	PF3D7_0206100	=	1.08	0.99
AL	PF3D7_1352500	=	1.08	0.95
AL	PF3D7_1226900	=	0.90	0.96
AL	PF3D7_1368800	=	1.39	0.93
AL	PF3D7_1432400	=	0.70	0.94

AL	PF3D7_1343900	=	0.80	0.98
AL	PF3D7_1328800	=	1.14	0.87
AL	PF3D7_1138700	=	1.44	1.00
AL	PF3D7_1327100	=	0.69	1.00
AL	PF3D7_1136600	=	0.85	1.00
AL	PF3D7_0902200	=	0.73	1.00
AL	PF3D7_0727100	=	1.92	0.99
AL	PF3D7_0105500	=	0.32	0.99
AL	PF3D7_1126100	=	0.46	0.97
AL	PF3D7_1343700	=	0.49	0.99
AL	PF3D7_1330500	=	0.79	1.00
AL	PF3D7_1343000	=	0.82	0.99
AL	PF3D7_1302000	=	0.60	0.98
AL	PF3D7_1107900	=	0.50	0.99
AL	PF3D7_0606000	=	0.72	0.99
AL	PF3D7_0614500	=	1.19	1.00
AL	PF3D7_0203700	=	0.84	0.99
AL	PF3D7_0601700	=	1.28	1.00
AL	PF3D7_1001900	=	2.25	0.98
AL	PF3D7_1409500	=	0.65	0.98
AL	PF3D7_0916700	=	1.79	0.99
AL	PF3D7_0402000	=	2.43	0.95
AL	PF3D7_0102600	=	0.52	0.97
AL	PF3D7_1433400	=	2.44	1.00
AL	PF3D7_0322000	=	0.91	0.98
AL	PF3D7_0623900	=	0.98	1.00
AL	PF3D7_1013500	=	0.79	0.99

C-MIR	PF3D7_0811300	=	0.16	0.95
C-MIR	PF3D7_1305500	=	0.35	0.94
C-MIR	PF3D7_0615900	=	1.32	0.99
C-MIR	PF3D7_1122900	=	1.20	0.93
C-MIR	PF3D7_1141900	=	0.48	0.97
C-MIR	PF3D7_1475600	=	0.38	0.96
C-MIR	PF3D7_0211100	=	0.86	1.00
C-MIR	PF3D7_0203000	=	0.00	0.50
C-MIR	PF3D7_0404600	=	0.71	0.95
C-MIR	PF3D7_0416500	=	3.91	0.91
C-MIR	PF3D7_1018400	=	1.57	0.95
C-MIR	PF3D7_0511500	=	0.75	0.98
C-MIR	PF3D7_1360200	=	0.84	0.90
C-MIR	PF3D7_0611800	=	2.65	0.99
C-MIR	PF3D7_0619900	=	2.21	0.99
C-MIR	PF3D7_0521900	=	4.02	1.00
C-MIR	PF3D7_1219300	=	3.34	0.96
C-MIR	PF3D7_1133700	=	2.34	0.94
C-MIR	PF3D7_0808700	=	1.29	0.98
C-MIR	PF3D7_1136000	=	2.18	0.94
C-MIR	PF3D7_0931000	=	7.02	0.98
C-MIR	PF3D7_0830900	=	8.82	0.94

C-MIR	PF3D7_1231800	=	9.94	0.93
C-MIR	PF3D7_1035800	=	6.01	0.96
C-MIR	PF3D7_1444100	=	4.00	0.95
C-MIR	PF3D7_1138900	=	8.87	0.92
C-MIR	PF3D7_1441800	=	0.61	0.96
C-MIR	PF3D7_1345800	=	2.07	0.99
C-MIR	PF3D7_1432400	=	1.83	0.94
C-MIR	PF3D7_0819800	=	0.32	0.93
C-MIR	PF3D7_1207800	=	2.35	0.96
C-MIR	PF3D7_1143500	=	0.50	0.96
C-MIR	PF3D7_1001600	=	1.44	0.98
C-MIR	PF3D7_1019400	=	1.32	0.91
C-MIR	PF3D7_1311900	=	2.14	0.99
C-MIR	PF3D7_1035700	=	1.26	0.97
C-MIR	PF3D7_1103100	=	1.37	0.96
C-MIR	PF3D7_0812500	=	2.10	0.90
C-MIR	PF3D7_1216100	=	0.06	0.99
C-MIR	PF3D7_0206100	=	0.85	0.99
C-MIR	PF3D7_1352500	=	0.95	0.98
C-MIR	PF3D7_1226900	=	0.24	0.99
C-MIR	PF3D7_1368800	=	0.95	0.98
C-MIR	PF3D7_1432400	=	0.90	0.97
C-MIR	PF3D7_1343900	=	0.60	0.96
C-MIR	PF3D7_1328800	=	0.82	0.96
C-MIR	PF3D7_1138700	=	0.89	1.00
C-MIR	PF3D7_1327100	=	0.18	0.99
C-MIR	PF3D7_1136600	=	0.57	0.98
C-MIR	PF3D7_0902200	=	0.69	0.96
C-MIR	PF3D7_0727100	=	1.27	1.00
C-MIR	PF3D7_0105500	=	0.17	0.99
C-MIR	PF3D7_1126100	=	0.29	0.98
C-MIR	PF3D7_1343700	=	0.22	0.99
C-MIR	PF3D7_1330500	=	2.68	0.99
C-MIR	PF3D7_1343000	=	1.29	0.98
C-MIR	PF3D7_1302000	=	0.77	0.98
C-MIR	PF3D7_1107900	=	1.11	0.99
C-MIR	PF3D7_0606000	=	2.94	0.99
C-MIR	PF3D7_0614500	=	5.85	0.99
C-MIR	PF3D7_0203700	=	4.64	0.97
C-MIR	PF3D7_0601700	=	4.33	0.99
C-MIR	PF3D7_1001900	=	4.96	0.97
C-MIR	PF3D7_1409500	=	2.15	0.98
C-MIR	PF3D7_0916700	=	1.59	0.98
C-MIR	PF3D7_0402000	=	2.59	0.99
C-MIR	PF3D7_0102600	=	0.06	0.79
C-MIR	PF3D7_1433400	=	1.18	0.98
C-MIR	PF3D7_0322000	=	4.49	0.98
C-MIR	PF3D7_0623900	=	2.82	0.99
C-MIR	PF3D7_1013500	=	0.81	0.73

T-MIR	PF3D7_0811300	=	0.10	0.91
T-MIR	PF3D7_1305500	=	0.51	0.97
T-MIR	PF3D7_0615900	=	1.40	1.00
T-MIR	PF3D7_1122900	=	0.95	0.93
T-MIR	PF3D7_1141900	=	0.91	0.99
T-MIR	PF3D7_1475600	=	0.81	0.98
T-MIR	PF3D7_0211100	=	1.33	1.00
T-MIR	PF3D7_0203000	=	0.21	0.94
T-MIR	PF3D7_0404600	=	0.82	1.00
T-MIR	PF3D7_0416500	=	1.76	0.95
T-MIR	PF3D7_1018400	=	0.76	0.97
T-MIR	PF3D7_0511500	=	0.41	0.98
T-MIR	PF3D7_1360200	=	1.81	0.98
T-MIR	PF3D7_0611800	=	1.00	0.98
T-MIR	PF3D7_0619900	=	1.06	1.00
T-MIR	PF3D7_0521900	=	2.25	0.99
T-MIR	PF3D7_1219300	=	1.15	0.98
T-MIR	PF3D7_1133700	=	1.68	0.96
T-MIR	PF3D7_0808700	=	1.12	0.98
T-MIR	PF3D7_1136000	=	1.90	0.98
T-MIR	PF3D7_0931000	=	6.40	0.97
T-MIR	PF3D7_0830900	=	7.99	0.99
T-MIR	PF3D7_1231800	=	8.71	0.98
T-MIR	PF3D7_1035800	=	5.97	0.99
T-MIR	PF3D7_1444100	=	5.48	0.98
T-MIR	PF3D7_1138900	=	8.84	0.99
T-MIR	PF3D7_1441800	=	1.07	0.99
T-MIR	PF3D7_1345800	=	0.75	1.00
T-MIR	PF3D7_1432400	=	2.60	0.99
T-MIR	PF3D7_0819800	=	1.41	0.98
T-MIR	PF3D7_1207800	=	1.70	0.99
T-MIR	PF3D7_1143500	=	0.65	0.99
T-MIR	PF3D7_1001600	=	1.28	1.00
T-MIR	PF3D7_1019400	=	2.26	1.00
T-MIR	PF3D7_1311900	=	1.70	1.00
T-MIR	PF3D7_1035700	=	1.49	0.98
T-MIR	PF3D7_1103100	=	1.23	1.00
T-MIR	PF3D7_0812500	=	2.10	0.96
T-MIR	PF3D7_1216100	=	0.04	0.97
T-MIR	PF3D7_0206100	=	0.93	0.98
T-MIR	PF3D7_1352500	=	0.88	0.99
T-MIR	PF3D7_1226900	=	0.17	0.98
T-MIR	PF3D7_1368800	=	0.93	0.98
T-MIR	PF3D7_1432400	=	0.70	0.96
T-MIR	PF3D7_1343900	=	0.61	0.97
T-MIR	PF3D7_1328800	=	0.77	0.93
T-MIR	PF3D7_1138700	=	0.94	1.00
T-MIR	PF3D7_1327100	=	0.22	0.99
T-MIR	PF3D7_1136600	=	0.43	0.97
T-MIR	PF3D7_0902200	=	1.05	0.96
T-MIR	PF3D7_0727100	=	1.23	0.99
T-MIR	PF3D7_0105500	=	0.30	0.98

T-MIR	PF3D7_1126100	=	0.33	0.98
T-MIR	PF3D7_1343700	=	0.29	0.99
T-MIR	PF3D7_1330500	=	1.76	1.00
T-MIR	PF3D7_1343000	=	0.97	0.99
T-MIR	PF3D7_1302000	=	1.27	0.99
T-MIR	PF3D7_1107900	=	0.59	0.98
T-MIR	PF3D7_0606000	=	1.71	0.98
T-MIR	PF3D7_0614500	=	2.47	0.99
T-MIR	PF3D7_0203700	=	2.71	0.98
T-MIR	PF3D7_0601700	=	2.74	0.99
T-MIR	PF3D7_1001900	=	4.58	0.99
T-MIR	PF3D7_1409500	=	2.06	0.98
T-MIR	PF3D7_0916700	=	1.60	0.98
T-MIR	PF3D7_0402000	=	3.30	0.99
T-MIR	PF3D7_0102600	=	1.02	0.98
T-MIR	PF3D7_1433400	=	1.88	0.99
T-MIR	PF3D7_0322000	=	6.12	1.00
T-MIR	PF3D7_0623900	=	1.64	0.98
T-MIR	PF3D7_1013500	=	2.76	1.00

SP	PF3D7_0811300	=	0.51	0.96
SP	PF3D7_1305500	=	0.41	0.96
SP	PF3D7_0615900	=	0.94	0.99
SP	PF3D7_1122900	=	0.57	0.94
SP	PF3D7_1141900	=	0.42	0.98
SP	PF3D7_1475600	=	0.67	1.00
SP	PF3D7_0211100	=	1.03	1.00
SP	PF3D7_0203000	=	0.17	0.86
SP	PF3D7_0404600	=	1.10	1.00
SP	PF3D7_0416500	=	1.08	1.00
SP	PF3D7_1018400	=	0.87	0.99
SP	PF3D7_0511500	=	1.00	1.00
SP	PF3D7_1360200	=	0.00	0.89
SP	PF3D7_0611800	=	1.12	0.98
SP	PF3D7_0619900	=	0.52	1.00
SP	PF3D7_0521900	=	1.15	1.00
SP	PF3D7_1219300	=	0.97	0.96
SP	PF3D7_1133700	=	0.64	0.91
SP	PF3D7_0808700	=	1.20	1.00
SP	PF3D7_1136000	=	1.20	0.91
SP	PF3D7_0931000	=	2.09	0.98
SP	PF3D7_0830900	=	1.97	0.97
SP	PF3D7_1231800	=	2.08	0.95
SP	PF3D7_1035800	=	1.96	0.99
SP	PF3D7_1444100	=	2.04	0.98
SP	PF3D7_1138900	=	2.64	0.97
SP	PF3D7_1441800	=	0.64	0.99
SP	PF3D7_1345800	=	0.64	0.99
SP	PF3D7_1432400	=	1.02	1.00
SP	PF3D7_0819800	=	0.91	1.00

SP	PF3D7_1207800	=	0.91	1.00
SP	PF3D7_1143500	=	0.98	1.00
SP	PF3D7_1001600	=	0.76	1.00
SP	PF3D7_1019400	=	0.74	0.99
SP	PF3D7_1311900	=	0.82	1.00
SP	PF3D7_1035700	=	1.65	0.97
SP	PF3D7_1103100	=	0.82	0.99
SP	PF3D7_0812500	=	0.25	0.90
SP	PF3D7_1216100	=	0.80	0.99
SP	PF3D7_0206100	=	1.00	0.99
SP	PF3D7_1352500	=	0.98	0.99
SP	PF3D7_1226900	=	0.60	0.99
SP	PF3D7_1368800	=	1.81	0.99
SP	PF3D7_1432400	=	1.01	0.97
SP	PF3D7_1343900	=	0.90	0.99
SP	PF3D7_1328800	=	0.96	0.95
SP	PF3D7_1138700	=	1.35	0.99
SP	PF3D7_1327100	=	0.54	0.99
SP	PF3D7_1136600	=	0.62	1.00
SP	PF3D7_0902200	=	0.83	0.96
SP	PF3D7_0727100	=	0.88	1.00
SP	PF3D7_0105500	=	0.61	0.99
SP	PF3D7_1126100	=	0.32	0.98
SP	PF3D7_1343700	=	0.35	0.99
SP	PF3D7_1330500	=	1.30	1.00
SP	PF3D7_1343000	=	0.95	0.98
SP	PF3D7_1302000	=	1.25	0.99
SP	PF3D7_1107900	=	0.76	0.99
SP	PF3D7_0606000	=	1.26	0.99
SP	PF3D7_0614500	=	1.34	0.97
SP	PF3D7_0203700	=	1.28	1.00
SP	PF3D7_0601700	=	1.26	1.00
SP	PF3D7_1001900	=	0.58	0.97
SP	PF3D7_1409500	=	0.76	0.98
SP	PF3D7_0916700	=	0.43	0.98
SP	PF3D7_0402000	=	1.09	0.99
SP	PF3D7_0102600	=	0.63	0.99
SP	PF3D7_1433400	=	0.33	0.95
SP	PF3D7_0322000	=	1.48	1.00
SP	PF3D7_0623900	=	0.27	0.97
SP	PF3D7_1013500	=	1.44	0.95

AM	PF3D7_0811300	=	0.17	0.90
AM	PF3D7_1305500	=	0.41	0.95
AM	PF3D7_0615900	=	0.54	0.97
AM	PF3D7_1122900	=	0.38	0.93
AM	PF3D7_1141900	=	0.25	0.96
AM	PF3D7_1475600	=	0.42	0.98
AM	PF3D7_0211100	=	1.79	1.00
AM	PF3D7_0203000	=	0.05	0.74

AM	PF3D7_0404600	=	1.97	0.97
AM	PF3D7_0416500	=	1.21	0.99
AM	PF3D7_1018400	=	0.54	0.98
AM	PF3D7_0511500	=	0.50	0.99
AM	PF3D7_1360200	=	0.82	0.87
AM	PF3D7_0611800	=	1.14	0.99
AM	PF3D7_0619900	=	0.70	1.00
AM	PF3D7_0521900	=	0.92	1.00
AM	PF3D7_1219300	=	0.88	1.00
AM	PF3D7_1133700	=	0.96	0.92
AM	PF3D7_0808700	=	0.54	0.98
AM	PF3D7_1136000	=	1.62	0.98
AM	PF3D7_0931000	=	3.29	0.99
AM	PF3D7_0830900	=	6.13	0.93
AM	PF3D7_1231800	=	6.60	0.95
AM	PF3D7_1035800	=	1.45	0.96
AM	PF3D7_1444100	=	3.08	0.95
AM	PF3D7_1138900	=	3.33	0.94
AM	PF3D7_1441800	=	0.37	0.93
AM	PF3D7_1345800	=	2.78	0.99
AM	PF3D7_1432400	=	0.79	0.98
AM	PF3D7_0819800	=	0.43	0.98
AM	PF3D7_1207800	=	2.94	1.00
AM	PF3D7_1143500	=	0.68	1.00
AM	PF3D7_1001600	=	1.08	1.00
AM	PF3D7_1019400	=	0.58	0.97
AM	PF3D7_1311900	=	4.60	0.99
AM	PF3D7_1035700	=	2.44	0.91
AM	PF3D7_1103100	=	2.93	1.00
AM	PF3D7_0812500	=	5.92	0.86
AM	PF3D7_1216100	=	0.75	0.98
AM	PF3D7_0206100	=	10.58	0.90
AM	PF3D7_1352500	=	9.29	0.87
AM	PF3D7_1226900	=	1.40	0.98
AM	PF3D7_1368800	=	16.88	0.87
AM	PF3D7_1432400	=	2.34	0.93
AM	PF3D7_1343900	=	4.40	0.85
AM	PF3D7_1328800	=	7.46	0.88
AM	PF3D7_1138700	=	0.85	0.99
AM	PF3D7_1327100	=	0.29	0.98
AM	PF3D7_1136600	=	0.67	0.99
AM	PF3D7_0902200	=	0.70	0.96
AM	PF3D7_0727100	=	1.20	0.99
AM	PF3D7_0105500	=	0.18	0.98
AM	PF3D7_1126100	=	0.52	0.97
AM	PF3D7_1343700	=	0.49	0.97
AM	PF3D7_1330500	=	0.67	0.98
AM	PF3D7_1343000	=	0.69	0.99
AM	PF3D7_1302000	=	0.55	0.98
AM	PF3D7_1107900	=	0.37	0.99
AM	PF3D7_0606000	=	1.26	0.98
AM	PF3D7_0614500	=	1.32	1.00

AM	PF3D7_0203700	=	1.03	0.97
AM	PF3D7_0601700	=	1.79	0.99
AM	PF3D7_1001900	=	7.42	0.98
AM	PF3D7_1409500	=	3.28	0.99
AM	PF3D7_0916700	=	5.29	0.99
AM	PF3D7_0402000	=	4.42	1.00
AM	PF3D7_0102600	=	1.13	0.99
AM	PF3D7_1433400	=	7.67	0.99
AM	PF3D7_0322000	=	1.35	0.98
AM	PF3D7_0623900	=	3.05	0.82
AM	PF3D7_1013500	=	1.09	0.95

MFQ	PF3D7_0811300	=	1.00	0.95
MFQ	PF3D7_1305500	=	1.15	0.99
MFQ	PF3D7_0615900	=	1.00	0.99
MFQ	PF3D7_1122900	=	1.00	0.98
MFQ	PF3D7_1141900	=	1.00	0.99
MFQ	PF3D7_1475600	=	1.00	0.98
MFQ	PF3D7_0211100	=	1.00	0.92
MFQ	PF3D7_0203000	=	1.00	0.99
MFQ	PF3D7_0404600	=	1.00	1.00
MFQ	PF3D7_0416500	=	0.52	0.97
MFQ	PF3D7_1018400	=	0.50	0.99
MFQ	PF3D7_0511500	=	0.39	0.99
MFQ	PF3D7_1360200	=	1.00	0.91
MFQ	PF3D7_0611800	=	0.93	0.98
MFQ	PF3D7_0619900	=	0.46	1.00
MFQ	PF3D7_0521900	=	0.51	1.00
MFQ	PF3D7_1219300	=	0.43	0.97
MFQ	PF3D7_1133700	=	1.01	0.99
MFQ	PF3D7_0808700	=	0.53	0.92
MFQ	PF3D7_1136000	=	1.72	1.00
MFQ	PF3D7_0931000	=	1.90	1.00
MFQ	PF3D7_0830900	=	2.65	0.99
MFQ	PF3D7_1231800	=	2.80	0.99
MFQ	PF3D7_1035800	=	1.01	0.97
MFQ	PF3D7_1444100	=	1.80	0.99
MFQ	PF3D7_1138900	=	1.66	0.98
MFQ	PF3D7_1441800	=	3.85	0.99
MFQ	PF3D7_1345800	=	4.10	0.93
MFQ	PF3D7_1432400	=	0.90	0.99
MFQ	PF3D7_0819800	=	1.50	1.00
MFQ	PF3D7_1207800	=	2.18	1.00
MFQ	PF3D7_1143500	=	0.56	0.99
MFQ	PF3D7_1001600	=	1.75	1.00
MFQ	PF3D7_1019400	=	1.28	0.99
MFQ	PF3D7_1311900	=	2.35	1.00
MFQ	PF3D7_1035700	=	4.56	0.99
MFQ	PF3D7_1103100	=	1.16	0.98
MFQ	PF3D7_0812500	=	0.77	0.90

MFQ	PF3D7_1216100	=	0.69	0.98
MFQ	PF3D7_0206100	=	3.53	0.94
MFQ	PF3D7_1352500	=	3.83	0.93
MFQ	PF3D7_1226900	=	0.70	0.99
MFQ	PF3D7_1368800	=	4.96	0.94
MFQ	PF3D7_1432400	=	2.54	0.91
MFQ	PF3D7_1343900	=	1.70	0.91
MFQ	PF3D7_1328800	=	2.63	0.89
MFQ	PF3D7_1138700	=	1.01	0.98
MFQ	PF3D7_1327100	=	0.30	0.99
MFQ	PF3D7_1136600	=	0.62	0.97
MFQ	PF3D7_0902200	=	0.90	0.97
MFQ	PF3D7_0727100	=	0.91	0.99
MFQ	PF3D7_0105500	=	0.40	0.98
MFQ	PF3D7_1126100	=	0.77	0.91
MFQ	PF3D7_1343700	=	0.57	0.99
MFQ	PF3D7_1330500	=	0.74	0.98
MFQ	PF3D7_1343000	=	1.29	0.99
MFQ	PF3D7_1302000	=	1.06	0.99
MFQ	PF3D7_1107900	=	0.77	0.99
MFQ	PF3D7_0606000	=	1.22	1.00
MFQ	PF3D7_0614500	=	1.50	0.99
MFQ	PF3D7_0203700	=	1.91	0.99
MFQ	PF3D7_0601700	=	1.54	1.00
MFQ	PF3D7_1001900	=	3.12	0.98
MFQ	PF3D7_1409500	=	0.87	0.98
MFQ	PF3D7_0916700	=	0.88	0.99
MFQ	PF3D7_0402000	=	1.15	1.00
MFQ	PF3D7_0102600	=	0.88	0.99
MFQ	PF3D7_1433400	=	1.19	0.98
MFQ	PF3D7_0322000	=	1.51	1.00
MFQ	PF3D7_0623900	=	2.48	0.91
MFQ	PF3D7_1013500	=	0.02	0.98

LUMF	PF3D7_0811300	=	0.25	0.95
LUMF	PF3D7_1305500	=	0.42	0.96
LUMF	PF3D7_0615900	=	0.66	0.94
LUMF	PF3D7_1122900	=	0.83	0.97
LUMF	PF3D7_1141900	=	0.40	0.97
LUMF	PF3D7_1475600	=	34.56	0.73
LUMF	PF3D7_0211100	=	1.25	1.00
LUMF	PF3D7_0203000	=	0.21	0.93
LUMF	PF3D7_0404600	=	0.67	0.96
LUMF	PF3D7_0416500	=	0.15	0.96
LUMF	PF3D7_1018400	=	0.22	0.96
LUMF	PF3D7_0511500	=	0.11	0.94
LUMF	PF3D7_1360200	=	1.05	0.92
LUMF	PF3D7_0611800	=	0.97	0.94
LUMF	PF3D7_0619900	=	0.44	0.97

LUMF	PF3D7_0521900	=	4.60	0.96
LUMF	PF3D7_1219300	=	0.82	0.91
LUMF	PF3D7_1133700	=	4.84	0.94
LUMF	PF3D7_0808700	=	2.45	0.91
LUMF	PF3D7_1136000	=	9.30	0.98
LUMF	PF3D7_0931000	=	20.59	0.98
LUMF	PF3D7_0830900	=	21.98	0.92
LUMF	PF3D7_1231800	=	24.91	0.91
LUMF	PF3D7_1035800	=	12.52	0.97
LUMF	PF3D7_1444100	=	21.35	0.97
LUMF	PF3D7_1138900	=	15.96	0.96
LUMF	PF3D7_1441800	=	0.44	0.84
LUMF	PF3D7_1345800	=	2.59	1.00
LUMF	PF3D7_1432400	=	0.93	1.00
LUMF	PF3D7_0819800	=	0.48	0.97
LUMF	PF3D7_1207800	=	0.88	0.99
LUMF	PF3D7_1143500	=	0.22	0.93
LUMF	PF3D7_1001600	=	0.63	0.96
LUMF	PF3D7_1019400	=	0.39	0.95
LUMF	PF3D7_1311900	=	34.56	0.98
LUMF	PF3D7_1035700	=	1.09	0.93
LUMF	PF3D7_1103100	=	4.00	0.92
LUMF	PF3D7_0812500	=	3.01	0.89
LUMF	PF3D7_1216100	=	0.79	0.98
LUMF	PF3D7_0206100	=	2.34	0.99
LUMF	PF3D7_1352500	=	1.43	1.00
LUMF	PF3D7_1226900	=	0.72	0.96
LUMF	PF3D7_1368800	=	2.95	1.00
LUMF	PF3D7_1432400	=	3.16	0.99
LUMF	PF3D7_1343900	=	0.76	1.00
LUMF	PF3D7_1328800	=	2.56	1.00
LUMF	PF3D7_1138700	=	0.75	1.00
LUMF	PF3D7_1327100	=	0.05	0.96
LUMF	PF3D7_1136600	=	0.41	0.96
LUMF	PF3D7_0902200	=	0.49	0.98
LUMF	PF3D7_0727100	=	0.63	0.96
LUMF	PF3D7_0105500	=	0.01	0.96
LUMF	PF3D7_1126100	=	0.15	0.98
LUMF	PF3D7_1343700	=	0.03	0.97
LUMF	PF3D7_1330500	=	2.20	0.99
LUMF	PF3D7_1343000	=	1.35	0.99
LUMF	PF3D7_1302000	=	2.40	0.99
LUMF	PF3D7_1107900	=	0.23	0.95
LUMF	PF3D7_0606000	=	2.50	0.94
LUMF	PF3D7_0614500	=	2.16	0.97
LUMF	PF3D7_0203700	=	1.79	0.94
LUMF	PF3D7_0601700	=	2.05	0.97
LUMF	PF3D7_1001900	=	2.98	0.98
LUMF	PF3D7_1409500	=	0.56	0.98
LUMF	PF3D7_0916700	=	0.78	0.99
LUMF	PF3D7_0402000	=	0.94	0.97
LUMF	PF3D7_0102600	=	0.74	0.96

LUMF	PF3D7_1433400	=	1.14	0.97
LUMF	PF3D7_0322000	=	1.38	0.97
LUMF	PF3D7_0623900	=	1.12	0.98
LUMF	PF3D7_1013500	=	0.11	0.97
PIP	PF3D7_0811300	=	0.32	0.93
PIP	PF3D7_1305500	=	0.74	0.95
PIP	PF3D7_0615900	=	1.12	0.97
PIP	PF3D7_1122900	=	0.43	0.92
PIP	PF3D7_1141900	=	0.50	0.97
PIP	PF3D7_1475600	=	0.67	0.97
PIP	PF3D7_0211100	=	0.19	1.00
PIP	PF3D7_0203000	=	0.28	0.94
PIP	PF3D7_0404600	=	0.73	0.92
PIP	PF3D7_0416500	=	0.26	0.86
PIP	PF3D7_1018400	=	0.82	0.94
PIP	PF3D7_0511500	=	0.77	0.98
PIP	PF3D7_1360200	=	0.61	0.89
PIP	PF3D7_0611800	=	0.93	0.96
PIP	PF3D7_0619900	=	0.73	1.00
PIP	PF3D7_0521900	=	1.07	0.99
PIP	PF3D7_1219300	=	0.97	0.92
PIP	PF3D7_1133700	=	0.99	0.95
PIP	PF3D7_0808700	=	1.15	0.94
PIP	PF3D7_1136000	=	1.14	0.99
PIP	PF3D7_0931000	=	1.57	0.94
PIP	PF3D7_0830900	=	0.66	0.82
PIP	PF3D7_1231800	=	1.98	0.93
PIP	PF3D7_1035800	=	1.86	0.98
PIP	PF3D7_1444100	=	1.92	0.94
PIP	PF3D7_1138900	=	0.68	0.84
PIP	PF3D7_1441800	=	0.60	0.90
PIP	PF3D7_1345800	=	1.20	0.98
PIP	PF3D7_1432400	=	0.76	0.97
PIP	PF3D7_0819800	=	0.71	0.95
PIP	PF3D7_1207800	=	0.82	1.00
PIP	PF3D7_1143500	=	0.72	0.94
PIP	PF3D7_1001600	=	0.68	1.00
PIP	PF3D7_1019400	=	0.85	0.94
PIP	PF3D7_1311900	=	0.62	0.96
PIP	PF3D7_1035700	=	0.70	0.90
PIP	PF3D7_1103100	=	0.63	0.96
PIP	PF3D7_0812500	=	0.31	0.87
PIP	PF3D7_1216100	=	0.90	0.98
PIP	PF3D7_0206100	=	1.05	0.99
PIP	PF3D7_1352500	=	1.05	0.99
PIP	PF3D7_1226900	=	0.66	0.97
PIP	PF3D7_1368800	=	1.13	0.99
PIP	PF3D7_1432400	=	1.14	0.98
PIP	PF3D7_1343900	=	0.83	1.00

PIP	PF3D7_1328800	=	0.82	0.98
PIP	PF3D7_1138700	=	1.33	1.00
PIP	PF3D7_1327100	=	0.51	0.99
PIP	PF3D7_1136600	=	0.59	0.99
PIP	PF3D7_0902200	=	0.74	0.97
PIP	PF3D7_0727100	=	0.73	0.99
PIP	PF3D7_0105500	=	0.28	0.98
PIP	PF3D7_1126100	=	0.57	0.99
PIP	PF3D7_1343700	=	0.23	0.99
PIP	PF3D7_1330500	=	0.98	1.00
PIP	PF3D7_1343000	=	0.59	0.98
PIP	PF3D7_1302000	=	1.01	0.99
PIP	PF3D7_1107900	=	1.17	0.98
PIP	PF3D7_0606000	=	1.02	1.00
PIP	PF3D7_0614500	=	1.07	0.99
PIP	PF3D7_0203700	=	1.56	0.98
PIP	PF3D7_0601700	=	0.65	0.99
PIP	PF3D7_1001900	=	2.17	0.99
PIP	PF3D7_1409500	=	0.98	0.98
PIP	PF3D7_0916700	=	0.94	0.99
PIP	PF3D7_0402000	=	1.21	0.99
PIP	PF3D7_0102600	=	0.81	0.98
PIP	PF3D7_1433400	=	1.02	0.99
PIP	PF3D7_0322000	=	1.34	0.99
PIP	PF3D7_0623900	=	1.82	0.98
	PF3D7_1013500	=	0.00	0.93

PHEN	PF3D7_0811300	=	1.08	0.97
PHEN	PF3D7_1305500	=	1.10	0.95
PHEN	PF3D7_0615900	=	1.40	0.97
PHEN	PF3D7_1122900	=	1.28	0.96
PHEN	PF3D7_1141900	=	1.86	0.97
PHEN	PF3D7_1475600	=	0.75	0.95
PHEN	PF3D7_0211100	=	0.84	0.98
PHEN	PF3D7_0203000	=	0.43	0.91
PHEN	PF3D7_0404600	=	1.81	0.98
PHEN	PF3D7_0416500	=	1.31	0.99
PHEN	PF3D7_1018400	=	1.91	0.98
PHEN	PF3D7_0511500	=	0.02	0.77
PHEN	PF3D7_1360200	=	2.00	0.98
PHEN	PF3D7_0611800	=	2.53	0.95
PHEN	PF3D7_0619900	=	0.92	0.98
PHEN	PF3D7_0521900	=	2.12	0.97
PHEN	PF3D7_1219300	=	2.08	0.99
PHEN	PF3D7_1133700	=	2.81	0.91
PHEN	PF3D7_0808700	=	0.77	0.95
PHEN	PF3D7_1136000	=	1.17	0.96
PHEN	PF3D7_0931000	=	0.81	1.00
PHEN	PF3D7_0830900	=	0.69	0.98
PHEN	PF3D7_1231800	=	1.19	0.98
PHEN	PF3D7_1035800	=	1.19	0.97

PHEN	PF3D7_1444100	=	1.17	0.99
PHEN	PF3D7_1138900	=	1.24	0.99
PHEN	PF3D7_1441800	=	1.41	0.96
PHEN	PF3D7_1345800	=	1.35	0.99
PHEN	PF3D7_1432400	=	1.78	1.00
PHEN	PF3D7_0819800	=	1.10	1.00
PHEN	PF3D7_1207800	=	1.02	0.94
PHEN	PF3D7_1143500	=	0.99	0.94
PHEN	PF3D7_1001600	=	1.20	0.95
PHEN	PF3D7_1019400	=	1.70	0.98
PHEN	PF3D7_1311900	=	0.93	0.96
PHEN	PF3D7_1035700	=	1.06	0.95
PHEN	PF3D7_1103100	=	1.29	1.00
PHEN	PF3D7_0812500	=	0.69	0.97
PHEN	PF3D7_1216100	=	0.39	1.00
PHEN	PF3D7_0206100	=	1.39	0.97
PHEN	PF3D7_1352500	=	0.84	1.00
PHEN	PF3D7_1226900	=	1.44	0.99
PHEN	PF3D7_1368800	=	0.20	0.96
PHEN	PF3D7_1432400	=	1.45	0.97
PHEN	PF3D7_1343900	=	0.67	0.98
PHEN	PF3D7_1328800	=	1.01	0.99
PHEN	PF3D7_1330500	=	1.46	0.98
PHEN	PF3D7_1343000	=	3.31	0.99
PHEN	PF3D7_1302000	=	3.00	0.99
PHEN	PF3D7_1107900	=	1.12	0.96
PHEN	PF3D7_0606000	=	3.12	0.98
PHEN	PF3D7_0614500	=	1.68	0.98
PHEN	PF3D7_0203700	=	0.83	0.89
PHEN	PF3D7_0601700	=	1.72	0.99
PHEN	PF3D7_1001900	=	1.18	0.92
PHEN	PF3D7_1409500	=	0.64	0.99
PHEN	PF3D7_0916700	=	0.59	0.99
PHEN	PF3D7_0402000	=	0.68	0.99
PHEN	PF3D7_0102600	=	2.22	0.87
PHEN	PF3D7_1433400	=	1.51	0.98
PHEN	PF3D7_0322000	=	1.01	0.99
PHEN	PF3D7_0623900	=	1.46	0.99
PHEN	PF3D7_1013500	=	0.81	0.99

ROT	PF3D7_0811300	=	0.51	0.92
ROT	PF3D7_1305500	=	0.69	0.96
ROT	PF3D7_0615900	=	0.89	0.97
ROT	PF3D7_1122900	=	0.04	0.97
ROT	PF3D7_1141900	=	0.39	0.97
ROT	PF3D7_1475600	=	0.65	0.98
ROT	PF3D7_0211100	=	0.30	0.95
ROT	PF3D7_0203000	=	0.15	0.78
ROT	PF3D7_0404600	=	6.94	0.96
ROT	PF3D7_0416500	=	3.70	0.94

ROT	PF3D7_1018400	=	4.10	0.97
ROT	PF3D7_0511500	=	1.28	0.94
ROT	PF3D7_1360200	=	2.92	0.97
ROT	PF3D7_0611800	=	25.53	0.97
ROT	PF3D7_0619900	=	3.70	0.95
ROT	PF3D7_0521900	=	12.80	0.96
ROT	PF3D7_1219300	=	3.70	0.89
ROT	PF3D7_1133700	=	13.75	0.89
ROT	PF3D7_0808700	=	3.35	0.95
ROT	PF3D7_1136000	=	0.62	0.92
ROT	PF3D7_0931000	=	6.22	0.97
ROT	PF3D7_0830900	=	3.85	0.99
ROT	PF3D7_1231800	=	13.22	0.96
ROT	PF3D7_1035800	=	24.88	0.97
ROT	PF3D7_1444100	=	11.51	0.93
ROT	PF3D7_1138900	=	10.99	0.93
ROT	PF3D7_1441800	=	0.21	0.85
ROT	PF3D7_1345800	=	2.32	0.98
ROT	PF3D7_1432400	=	0.70	0.98
ROT	PF3D7_0819800	=	0.69	0.95
ROT	PF3D7_1207800	=	1.08	1.00
ROT	PF3D7_1143500	=	0.08	0.92
ROT	PF3D7_1001600	=	0.51	0.98
ROT	PF3D7_1019400	=	2.64	0.96
ROT	PF3D7_1311900	=	8.80	0.99
ROT	PF3D7_1035700	=	2.50	0.94
ROT	PF3D7_1103100	=	1.60	0.99
ROT	PF3D7_0812500	=	9.80	0.96
ROT	PF3D7_1216100	=	0.68	0.99
ROT	PF3D7_0206100	=	1.35	0.98
ROT	PF3D7_1352500	=	1.29	0.99
ROT	PF3D7_1226900	=	0.44	0.98
ROT	PF3D7_1368800	=	2.16	0.99
ROT	PF3D7_1432400	=	1.49	0.99
ROT	PF3D7_1343900	=	1.22	0.98
ROT	PF3D7_1328800	=	1.15	0.99
ROT	PF3D7_1138700	=	0.29	1.00
ROT	PF3D7_1327100	=	0.17	0.99
ROT	PF3D7_1136600	=	0.41	0.99
ROT	PF3D7_0902200	=	0.06	0.99
ROT	PF3D7_0727100	=	0.11	0.99
ROT	PF3D7_0105500	=	0.05	0.97
ROT	PF3D7_1126100	=	0.05	0.97
ROT	PF3D7_1343700	=	0.05	0.98
ROT	PF3D7_1330500	=	0.27	0.98
ROT	PF3D7_1343000	=	0.11	0.98
ROT	PF3D7_1302000	=	0.15	0.98
ROT	PF3D7_1107900	=	0.02	0.97
ROT	PF3D7_0606000	=	0.13	0.99
ROT	PF3D7_0614500	=	0.36	0.99
ROT	PF3D7_0203700	=	0.06	0.97
ROT	PF3D7_0601700	=	0.10	0.99

ROT	PF3D7_1001900	=	1.96	0.86
ROT	PF3D7_1409500	=	4.58	0.95
ROT	PF3D7_0916700	=	1.27	0.97
ROT	PF3D7_0402000	=	3.27	0.97
ROT	PF3D7_0102600	=	0.69	0.96
ROT	PF3D7_1433400	=	0.96	0.95
ROT	PF3D7_0322000	=	6.76	0.98
ROT	PF3D7_0623900	=	2.54	0.97
ROT	PF3D7_1013500	=	1.33	0.96

TTFA	PF3D7_0811300	=	0.79	0.99
TTFA	PF3D7_1305500	=	0.78	0.98
TTFA	PF3D7_0615900	=	0.64	0.92
TTFA	PF3D7_1122900	=	0.56	0.95
TTFA	PF3D7_1141900	=	0.58	0.92
TTFA	PF3D7_1475600	=	0.64	0.95
TTFA	PF3D7_0211100	=	0.32	0.99
TTFA	PF3D7_0203000	=	0.35	0.98
TTFA	PF3D7_0404600	=	1.10	0.92
TTFA	PF3D7_0416500	=	0.30	0.93
TTFA	PF3D7_1018400	=	1.50	0.95
TTFA	PF3D7_0511500	=	0.00	0.72
TTFA	PF3D7_1360200	=	0.66	0.99
TTFA	PF3D7_0611800	=	0.11	0.94
TTFA	PF3D7_0619900	=	0.49	0.98
TTFA	PF3D7_0521900	=	2.61	0.98
TTFA	PF3D7_1219300	=	1.93	1.00
TTFA	PF3D7_1133700	=	1.14	1.00
TTFA	PF3D7_0808700	=	2.09	0.97
TTFA	PF3D7_1136000	=	1.53	0.98
TTFA	PF3D7_0931000	=	2.81	0.99
TTFA	PF3D7_0830900	=	0.92	0.94
TTFA	PF3D7_1231800	=	3.09	0.96
TTFA	PF3D7_1035800	=	1.97	0.95
TTFA	PF3D7_1444100	=	4.26	0.98
TTFA	PF3D7_1138900	=	1.97	0.98
TTFA	PF3D7_1441800	=	0.73	0.97
TTFA	PF3D7_1345800	=	0.69	0.99
TTFA	PF3D7_1432400	=	2.09	0.99
TTFA	PF3D7_0819800	=	0.82	0.98
TTFA	PF3D7_1207800	=	0.09	0.87
TTFA	PF3D7_1143500	=	0.64	0.93
TTFA	PF3D7_1001600	=	0.40	0.97
TTFA	PF3D7_1019400	=	1.37	0.98
TTFA	PF3D7_1311900	=	0.88	0.98
TTFA	PF3D7_1035700	=	0.63	0.98
TTFA	PF3D7_1103100	=	1.43	1.00
TTFA	PF3D7_0812500	=	0.49	0.99
TTFA	PF3D7_1216100	=	0.41	0.99
TTFA	PF3D7_0206100	=	0.66	0.99

TTFA	PF3D7_1352500	=	0.54	0.99
TTFA	PF3D7_1226900	=	0.91	0.97
TTFA	PF3D7_1368800	=	0.36	0.98
TTFA	PF3D7_1432400	=	6.76	0.99
TTFA	PF3D7_1343900	=	0.84	0.98
TTFA	PF3D7_1328800	=	0.57	0.91
TTFA	PF3D7_1138700	=	0.23	0.95
TTFA	PF3D7_1327100	=	0.18	0.91
TTFA	PF3D7_1136600	=	0.10	0.91
TTFA	PF3D7_0902200	=	0.25	0.92
TTFA	PF3D7_0727100	=	0.10	0.89
TTFA	PF3D7_0105500	=	0.21	0.93
TTFA	PF3D7_1126100	=	0.11	0.92
TTFA	PF3D7_1343700	=	0.05	0.89
TTFA	PF3D7_1330500	=	1.19	0.98
TTFA	PF3D7_1343000	=	1.83	0.98
TTFA	PF3D7_1302000	=	2.67	0.99
TTFA	PF3D7_1107900	=	1.10	0.98
TTFA	PF3D7_0606000	=	1.71	0.97
TTFA	PF3D7_0614500	=	0.98	0.96
TTFA	PF3D7_0203700	=	1.08	0.98
TTFA	PF3D7_0601700	=	0.90	0.99
TTFA	PF3D7_1001900	=	0.51	0.93
TTFA	PF3D7_1409500	=	0.50	0.99
TTFA	PF3D7_0916700	=	0.34	0.99
TTFA	PF3D7_0402000	=	0.89	1.00
TTFA	PF3D7_0102600	=	0.84	0.97
TTFA	PF3D7_1433400	=	0.80	0.99
TTFA	PF3D7_0322000	=	0.70	0.96
TTFA	PF3D7_0623900	=	0.68	0.98
TTFA	PF3D7_1013500	=	0.51	0.99

EHNA	PF3D7_0811300	=	0.35	0.98
EHNA	PF3D7_1305500	=	0.33	0.98
EHNA	PF3D7_0615900	=	0.23	0.91
EHNA	PF3D7_1122900	=	0.12	0.90
EHNA	PF3D7_1141900	=	0.66	0.93
EHNA	PF3D7_1475600	=	0.06	0.93
EHNA	PF3D7_0211100	=	0.12	0.93
EHNA	PF3D7_0203000	=	0.21	0.95
EHNA	PF3D7_0404600	=	0.95	0.99
EHNA	PF3D7_0416500	=	0.18	0.97
EHNA	PF3D7_1018400	=	1.22	0.97
EHNA	PF3D7_0511500	=	0.00	0.65
EHNA	PF3D7_1360200	=	0.47	0.97
EHNA	PF3D7_0611800	=	0.31	0.98
EHNA	PF3D7_0619900	=	1.07	0.97
EHNA	PF3D7_0521900	=	0.98	0.96
EHNA	PF3D7_1219300	=	1.32	0.99
EHNA	PF3D7_1133700	=	0.27	0.93

EHNA	PF3D7_0808700	=	0.20	0.98
EHNA	PF3D7_1136000	=	0.32	0.97
EHNA	PF3D7_0931000	=	0.76	0.99
EHNA	PF3D7_0830900	=	0.11	0.94
EHNA	PF3D7_1231800	=	1.22	0.93
EHNA	PF3D7_1035800	=	0.83	0.96
EHNA	PF3D7_1444100	=	1.38	0.97
EHNA	PF3D7_0203600	=	0.03	0.90
EHNA	PF3D7_1138900	=	0.40	0.95
EHNA	PF3D7_1441800	=	0.48	0.97
EHNA	PF3D7_1345800	=	0.80	1.00
EHNA	PF3D7_1432400	=	1.87	0.99
EHNA	PF3D7_0819800	=	0.45	1.00
EHNA	PF3D7_1207800	=	0.07	0.92
EHNA	PF3D7_1143500	=	0.76	0.93
EHNA	PF3D7_1001600	=	0.12	0.93
EHNA	PF3D7_1019400	=	0.52	0.95
EHNA	PF3D7_1311900	=	0.35	0.93
EHNA	PF3D7_1035700	=	0.46	0.99
EHNA	PF3D7_1103100	=	3.10	0.99
EHNA	PF3D7_0812500	=	0.42	0.96
EHNA	PF3D7_1216100	=	0.82	0.97
EHNA	PF3D7_0206100	=	0.31	0.94
EHNA	PF3D7_1352500	=	0.45	0.98
EHNA	PF3D7_1226900	=	0.65	0.99
EHNA	PF3D7_1368800	=	0.17	0.98
EHNA	PF3D7_1432400	=	1.31	0.98
EHNA	PF3D7_1343900	=	0.97	0.98
EHNA	PF3D7_1328800	=	0.37	0.97
EHNA	PF3D7_1138700	=	0.81	0.98
EHNA	PF3D7_1327100	=	0.72	0.99
EHNA	PF3D7_1136600	=	0.34	0.96
EHNA	PF3D7_0902200	=	0.18	0.97
EHNA	PF3D7_0727100	=	0.28	0.98
EHNA	PF3D7_0105500	=	0.39	0.98
EHNA	PF3D7_1126100	=	0.05	0.94
EHNA	PF3D7_1343700	=	0.21	0.96
EHNA	PF3D7_1330500	=	1.61	0.99
EHNA	PF3D7_1343000	=	3.93	0.98
EHNA	PF3D7_1302000	=	2.62	0.98
EHNA	PF3D7_0203700	=	1.13	0.99
EHNA	PF3D7_1107900	=	1.41	0.97
EHNA	PF3D7_0606000	=	2.61	0.97
EHNA	PF3D7_0614500	=	1.51	0.98
EHNA	PF3D7_0203700	=	0.44	0.98
EHNA	PF3D7_0601700	=	1.28	0.99
ALLO	PF3D7_0811300	=	0.01	0.61
ALLO	PF3D7_1305500	=	1.14	0.96
ALLO	PF3D7_0615900	=	1.16	0.92

ALLO	PF3D7_1122900	=	0.70	0.99
ALLO	PF3D7_1141900	=	0.98	0.97
ALLO	PF3D7_1475600	=	0.48	0.95
ALLO	PF3D7_0211100	=	0.26	0.96
ALLO	PF3D7_0203000	=	0.53	0.98
ALLO	PF3D7_0404600	=	1.08	1.00
ALLO	PF3D7_0416500	=	0.41	0.99
ALLO	PF3D7_1018400	=	1.51	1.00
ALLO	PF3D7_0511500	=	0.00	0.74
ALLO	PF3D7_1360200	=	1.14	0.99
ALLO	PF3D7_0611800	=	1.12	0.98
ALLO	PF3D7_0619900	=	1.36	0.99
ALLO	PF3D7_0521900	=	1.46	1.00
ALLO	PF3D7_1219300	=	1.45	1.00
ALLO	PF3D7_1133700	=	1.61	1.00
ALLO	PF3D7_0808700	=	1.60	0.96
ALLO	PF3D7_1136000	=	1.01	0.99
ALLO	PF3D7_0931000	=	1.40	1.00
ALLO	PF3D7_0830900	=	0.63	0.98
ALLO	PF3D7_1231800	=	1.61	0.97
ALLO	PF3D7_1035800	=	1.52	1.00
ALLO	PF3D7_1444100	=	1.80	1.00
ALLO	PF3D7_1138900	=	1.18	0.99
ALLO	PF3D7_1441800	=	0.46	0.95
ALLO	PF3D7_1345800	=	0.99	1.00
ALLO	PF3D7_1432400	=	1.18	1.00
ALLO	PF3D7_0819800	=	0.55	0.97
ALLO	PF3D7_1207800	=	0.34	0.97
ALLO	PF3D7_1143500	=	0.59	0.95
ALLO	PF3D7_1001600	=	0.34	0.95
ALLO	PF3D7_1019400	=	0.54	0.95
ALLO	PF3D7_1311900	=	0.14	0.95
ALLO	PF3D7_1035700	=	0.56	0.99
ALLO	PF3D7_1103100	=	1.42	0.99
ALLO	PF3D7_0812500	=	1.06	1.00
ALLO	PF3D7_1216100	=	0.84	1.00
ALLO	PF3D7_0206100	=	0.80	1.00
ALLO	PF3D7_1352500	=	0.59	0.99
ALLO	PF3D7_1226900	=	0.90	0.96
ALLO	PF3D7_1368800	=	0.18	0.99
ALLO	PF3D7_1432400	=	1.84	1.00
ALLO	PF3D7_1343900	=	0.63	0.97
ALLO	PF3D7_1328800	=	3.76	0.89
ALLO	PF3D7_1138700	=	0.78	1.00
ALLO	PF3D7_1327100	=	0.60	0.98
ALLO	PF3D7_1136600	=	0.69	1.00
ALLO	PF3D7_0902200	=	0.40	0.97
ALLO	PF3D7_0727100	=	0.39	0.99
ALLO	PF3D7_0105500	=	0.39	0.98
ALLO	PF3D7_1126100	=	0.23	0.99
ALLO	PF3D7_1343700	=	0.16	0.99
ALLO	PF3D7_1330500	=	0.91	0.98

ALLO	PF3D7_1343000	=	1.57	1.00
ALLO	PF3D7_1302000	=	1.26	0.99
ALLO	PF3D7_1107900	=	1.78	0.96
ALLO	PF3D7_0606000	=	1.22	0.98
ALLO	PF3D7_0614500	=	0.58	0.99
ALLO	PF3D7_0203700	=	1.60	1.00
ALLO	PF3D7_0601700	=	0.83	1.00
ALLO	PF3D7_1001900	=	1.21	0.91
ALLO	PF3D7_1409500	=	1.69	0.98
ALLO	PF3D7_0916700	=	1.08	0.97
ALLO	PF3D7_0402000	=	0.37	0.95
ALLO	PF3D7_0102600	=	1.33	1.00
ALLO	PF3D7_1433400	=	2.15	0.95
ALLO	PF3D7_0322000	=	0.43	0.90
ALLO	PF3D7_0623900	=	1.20	0.99
ALLO	PF3D7_1013500	=	0.69	1.00

PYR	PF3D7_0811300	=	100.31	0.96
PYR	PF3D7_1305500	=	272.51	0.97
PYR	PF3D7_0615900	=	392.63	0.99
PYR	PF3D7_1122900	=	263.54	0.97
PYR	PF3D7_1141900	=	289.38	0.99
PYR	PF3D7_1475600	=	201.92	0.99
PYR	PF3D7_0211100	=	468.35	1.00
PYR	PF3D7_0203000	=	59.08	0.82
PYR	PF3D7_0404600	=	78.28	0.95
PYR	PF3D7_0416500	=	183.06	0.87
PYR	PF3D7_1018400	=	157.30	0.92
PYR	PF3D7_0511500	=	201.12	0.99
PYR	PF3D7_1360200	=	132.86	0.96
PYR	PF3D7_0611800	=	1.14	0.98
PYR	PF3D7_0619900	=	143.53	0.98
PYR	PF3D7_0521900	=	1.85	0.99
PYR	PF3D7_1219300	=	1.29	0.97
PYR	PF3D7_1133700	=	48.02	0.97
PYR	PF3D7_0808700	=	0.65	0.93
PYR	PF3D7_1136000	=	105.98	0.99
PYR	PF3D7_0931000	=	175.60	0.95
PYR	PF3D7_0830900	=	150.32	0.92
PYR	PF3D7_1231800	=	159.32	0.97
PYR	PF3D7_1035800	=	129.45	0.98
PYR	PF3D7_1444100	=	153.17	0.96
PYR	PF3D7_1138900	=	64.16	0.89
PYR	PF3D7_1441800	=	77.18	0.87
PYR	PF3D7_1345800	=	152.20	1.00
PYR	PF3D7_1432400	=	120.75	0.97
PYR	PF3D7_0819800	=	95.72	0.96
PYR	PF3D7_1207800	=	1.41	0.96
PYR	PF3D7_1143500	=	105.64	0.95
PYR	PF3D7_1001600	=	97.59	0.99

PYR	PF3D7_1019400	=	102.46	0.97
PYR	PF3D7_1311900	=	88.83	0.94
PYR	PF3D7_1035700	=	58.09	0.93
PYR	PF3D7_1103100	=	80.33	0.92
PYR	PF3D7_0812500	=	94.60	0.92
PYR	PF3D7_1216100	=	86.00	0.98
PYR	PF3D7_0206100	=	174.13	1.00
PYR	PF3D7_1352500	=	0.84	0.99
PYR	PF3D7_1226900	=	69.46	0.99
PYR	PF3D7_1368800	=	165.63	0.99
PYR	PF3D7_1432400	=	135.48	0.99
PYR	PF3D7_1343900	=	94.89	1.00
PYR	PF3D7_1328800	=	101.21	0.98
PYR	PF3D7_1138700	=	644.93	1.00
PYR	PF3D7_1327100	=	294.09	0.99
PYR	PF3D7_1136600	=	453.49	0.99
PYR	PF3D7_0902200	=	437.83	0.98
PYR	PF3D7_0727100	=	415.68	0.99
PYR	PF3D7_0105500	=	326.95	0.98
PYR	PF3D7_1126100	=	332.55	0.97
PYR	PF3D7_1343700	=	242.07	1.00
PYR	PF3D7_1330500	=	1191.52	1.00
PYR	PF3D7_1343000	=	883.42	0.97
PYR	PF3D7_1302000	=	1.01	0.99
PYR	PF3D7_1107900	=	388.92	0.98
PYR	PF3D7_0606000	=	1147.52	0.99
PYR	PF3D7_0614500	=	1049.70	0.99
PYR	PF3D7_0203700	=	1092.31	1.00
PYR	PF3D7_0601700	=	841.72	0.99
PYR	PF3D7_1001900	=	936.48	0.98
PYR	PF3D7_1409500	=	583.59	0.98
PYR	PF3D7_0916700	=	479.53	1.00
PYR	PF3D7_0402000	=	0.66	0.98
PYR	PF3D7_0102600	=	5.00	0.96
PYR	PF3D7_1433400	=	4.01	0.96
PYR	PF3D7_0322000	=	372.31	0.99
PYR	PF3D7_0623900	=	502.36	0.99
PYR	PF3D7_1013500	=	828.49	1.00

CG	PF3D7_0811300	=	2310.00	0.97
CG	PF3D7_1305500	=	3120.00	0.99
CG	PF3D7_0615900	=	3970.00	0.98
CG	PF3D7_1122900	=	2600.00	0.95
CG	PF3D7_1141900	=	4720.00	0.95
CG	PF3D7_1475600	=	680.00	0.97
CG	PF3D7_0211100	=	0.34	0.92
CG	PF3D7_0203000	=	3710.00	0.99
CG	PF3D7_0404600	=	4320.00	1.00
CG	PF3D7_0416500	=	630.82	0.98
CG	PF3D7_1018400	=	810.37	0.96

CG	PF3D7_0511500	=	1147.62	0.97
CG	PF3D7_1360200	=	778.89	0.97
CG	PF3D7_0611800	=	780.32	0.98
CG	PF3D7_0619900	=	390.04	0.97
CG	PF3D7_0521900	=	478.36	0.94
CG	PF3D7_1219300	=	1.87	0.97
CG	PF3D7_1133700	=	690.78	0.98
CG	PF3D7_0808700	=	2.22	0.96
CG	PF3D7_1136000	=	733.71	0.97
CG	PF3D7_0931000	=	468.74	0.96
CG	PF3D7_0830900	=	468.74	0.92
CG	PF3D7_1231800	=	792.52	0.94
CG	PF3D7_1035800	=	754.98	0.96
CG	PF3D7_1444100	=	833.68	0.95
CG	PF3D7_0203600	=	1.40	0.98
CG	PF3D7_1138900	=	77.81	0.97
CG	PF3D7_1441800	=	400.58	0.82
CG	PF3D7_1345800	=	420.65	1.00
CG	PF3D7_1432400	=	616.08	1.00
CG	PF3D7_0819800	=	565.96	1.00
CG	PF3D7_1207800	=	30.50	0.98
CG	PF3D7_1143500	=	402.95	0.96
CG	PF3D7_1001600	=	269.30	0.99
CG	PF3D7_1019400	=	274.44	0.99
CG	PF3D7_1311900	=	274.44	0.99
CG	PF3D7_1035700	=	623.52	0.97
CG	PF3D7_1103100	=	661.37	1.00
CG	PF3D7_0812500	=	602.96	0.97
CG	PF3D7_1216100	=	1293.05	1.00
CG	PF3D7_0206100	=	786.55	0.96
CG	PF3D7_1352500	=	0.81	0.88
CG	PF3D7_1226900	=	1140.01	0.96
CG	PF3D7_1368800	=	584.72	0.98
CG	PF3D7_1432400	=	1140.68	0.99
CG	PF3D7_1343900	=	784.93	0.95
CG	PF3D7_1328800	=	460.72	0.97
CG	PF3D7_1138700	=	14040.00	0.98
CG	PF3D7_1327100	=	9984.50	0.99
CG	PF3D7_1136600	=	15021.25	0.99
CG	PF3D7_0902200	=	13466.00	0.99
CG	PF3D7_0727100	=	2969.25	0.97
CG	PF3D7_0105500	=	18275.25	1.00
CG	PF3D7_1126100	=	7807.50	0.98
CG	PF3D7_1343700	=	9331.50	0.99
CG	PF3D7_1330500	=	4552.30	1.00
CG	PF3D7_1343000	=	4467.18	0.99
CG	PF3D7_1302000	=	1.83	0.98
CG	PF3D7_0203700	=	1924.73	0.99
CG	PF3D7_1107900	=	3938.05	0.98
CG	PF3D7_0606000	=	5120.33	1.00
CG	PF3D7_0614500	=	2717.95	0.99
CG	PF3D7_0203700	=	3374.58	0.99

CG	PF3D7_0601700	=	2697.43	0.99
SDZ	PF3D7_0811300	=	0.24	0.92
SDZ	PF3D7_1305500	=	0.55	0.97
SDZ	PF3D7_0615900	=	0.87	1.00
SDZ	PF3D7_1122900	=	0.40	0.96
SDZ	PF3D7_1141900	=	0.98	0.97
SDZ	PF3D7_1475600	=	0.76	1.00
SDZ	PF3D7_0211100	=	0.15	0.87
SDZ	PF3D7_0203000	=	0.73	0.91
SDZ	PF3D7_0404600	=	0.32	0.94
SDZ	PF3D7_0416500	=	2.54	0.96
SDZ	PF3D7_1018400	=	1.35	0.89
SDZ	PF3D7_0511500	=	2.55	0.96
SDZ	PF3D7_1360200	=	0.87	0.98
SDZ	PF3D7_0611800	=	2.29	0.98
SDZ	PF3D7_0619900	=	1.07	0.97
SDZ	PF3D7_0521900	=	2.63	0.98
SDZ	PF3D7_1219300	=	2.07	0.95
SDZ	PF3D7_1133700	=	1.00	0.99
SDZ	PF3D7_0808700	=	1.00	0.99
SDZ	PF3D7_1432400	=	0.77	0.96
SDZ	PF3D7_0819800	=	0.47	0.99
SDZ	PF3D7_1207800	=	0.69	0.98
SDZ	PF3D7_1143500	=	0.68	0.99
SDZ	PF3D7_1001600	=	0.66	0.97
SDZ	PF3D7_1019400	=	1.96	0.99
SDZ	PF3D7_1311900	=	0.49	1.00
SDZ	PF3D7_1035700	=	0.46	0.99
SDZ	PF3D7_1103100	=	0.05	0.99
SDZ	PF3D7_0812500	=	0.67	0.83
SDZ	PF3D7_1216100	=	0.66	1.00
SDZ	PF3D7_0206100	=	0.80	1.00
SDZ	PF3D7_1352500	=	0.80	1.00
SDZ	PF3D7_1226900	=	2.51	0.99
SDZ	PF3D7_1368800	=	0.93	1.00
SDZ	PF3D7_1432400	=	0.69	1.00
SDZ	PF3D7_1343900	=	0.58	0.99
SDZ	PF3D7_1328800	=	0.95	1.00
SDZ	PF3D7_1138700	=	0.57	0.98
SDZ	PF3D7_1327100	=	0.23	0.98
SDZ	PF3D7_1136600	=	0.55	0.99
SDZ	PF3D7_0902200	=	0.67	1.00
SDZ	PF3D7_0727100	=	0.66	0.99
SDZ	PF3D7_0105500	=	0.37	0.99
SDZ	PF3D7_1126100	=	0.41	0.97
SDZ	PF3D7_1343700	=	0.20	0.99
SDZ	PF3D7_1330500	=	1.54	0.99
SDZ	PF3D7_1343000	=	0.71	0.97
SDZ	PF3D7_1302000	=	1.88	0.99

SDZ	PF3D7_0203700	=	2.44	1.00
SDZ	PF3D7_1107900	=	0.58	0.99
SDZ	PF3D7_0606000	=	1.58	0.99
SDZ	PF3D7_0614500	=	1.46	0.98
SDZ	PF3D7_0203700	=	1.35	0.99
SDZ	PF3D7_0601700	=	1.33	0.99
SDZ	PF3D7_0702300	=	1.71	0.98
SDZ	PF3D7_1001900	=	0.55	0.96
SDZ	PF3D7_1409500	=	0.06	0.99
SDZ	PF3D7_0916700	=	0.81	1.00
SDZ	PF3D7_0402000	=	0.90	0.99
SDZ	PF3D7_0102600	=	0.86	0.99
SDZ	PF3D7_1433400	=	0.92	0.99
SDZ	PF3D7_0322000	=	0.85	1.00
SDZ	PF3D7_0623900	=	1.26	0.99
SDZ	PF3D7_0103300	>	3330 uM	
SDZ	PF3D7_1013500	=	10.07	0.96

IB	PF3D7_0811300	=	6.97	0.98
IB	PF3D7_1305500	=	1.12	0.99
IB	PF3D7_0615900	=	0.15	0.79
IB	PF3D7_1122900	=	4.17	0.98
IB	PF3D7_1141900	=	0.65	0.92
IB	PF3D7_1475600	=	1.30	0.96
IB	PF3D7_0211100	=	0.41	0.84
IB	PF3D7_0203000	=	0.73	0.86
IB	PF3D7_0404600	=	1.00	0.99
IB	PF3D7_0416500	=	0.34	0.99
IB	PF3D7_1018400	=	0.41	0.98
IB	PF3D7_0511500	=	1.51	0.98
IB	PF3D7_1360200	=	0.38	0.99
IB	PF3D7_0611800	=	0.43	0.98
IB	PF3D7_0619900	=	0.91	0.99
IB	PF3D7_0521900	=	0.46	0.86
IB	PF3D7_1219300	=	0.49	0.98
IB	PF3D7_1133700	=	1.24	0.99
IB	PF3D7_0808700	=	3.63	0.96
IB	PF3D7_1136000	=	2.47	0.98
IB	PF3D7_0931000	=	1.71	1.00
IB	PF3D7_0830900	=	0.93	0.99
IB	PF3D7_1231800	=	1.67	0.97
IB	PF3D7_1035800	=	7.02	0.94
IB	PF3D7_1444100	=	1.87	0.90
IB	PF3D7_1138900	=	0.86	0.84
IB	PF3D7_1441800	=	0.59	0.84
IB	PF3D7_1345800	=	0.72	0.91
IB	PF3D7_1432400	=	0.88	1.00
IB	PF3D7_0819800	=	0.74	1.00
IB	PF3D7_1207800	=	1.08	0.99
IB	PF3D7_1143500	=	0.75	0.99

IB	PF3D7_1001600	=	0.47	0.99
IB	PF3D7_1019400	=	0.67	0.99
IB	PF3D7_1311900	=	5.86	0.89
IB	PF3D7_1035700	=	0.76	0.93
IB	PF3D7_1103100	=	0.89	1.00
IB	PF3D7_0812500	=	1.00	0.98
IB	PF3D7_1216100	=	4.68	1.00
IB	PF3D7_0206100	=	0.81	0.99
IB	PF3D7_1352500	=	0.67	1.00
IB	PF3D7_1226900	=	1.71	0.99
IB	PF3D7_1368800	=	0.98	1.00
IB	PF3D7_1432400	=	2.99	0.99
IB	PF3D7_1343900	=	0.17	0.89
IB	PF3D7_1328800	=	0.26	0.88
IB	PF3D7_1138700	=	0.22	0.98
IB	PF3D7_1327100	=	0.45	0.94
IB	PF3D7_1136600	=	0.33	1.00
IB	PF3D7_0902200	=	0.33	0.98
IB	PF3D7_0727100	=	0.23	1.00
IB	PF3D7_0105500	=	0.37	0.90
IB	PF3D7_1126100	=	0.22	0.96
IB	PF3D7_1343700	=	0.21	0.99
IB	PF3D7_1330500	=	1.40	0.92
IB	PF3D7_1343000	=	2.83	0.99
IB	PF3D7_1302000	=	2.75	0.95
IB	PF3D7_1107900	=	1.78	0.98
IB	PF3D7_0606000	=	1.57	0.94
IB	PF3D7_0614500	=	1.20	0.91
IB	PF3D7_0203700	=	1.07	0.93
IB	PF3D7_0601700	=	1.44	0.87
IB	PF3D7_1001900	=	1.68	0.97
IB	PF3D7_1409500	=	1.65	0.99
IB	PF3D7_0916700	=	1.25	0.89
IB	PF3D7_0402000	=	1.18	0.94
IB	PF3D7_0102600	=	0.79	0.97
IB	PF3D7_1433400	=	1.76	0.94
IB	PF3D7_0322000	=	1.33	0.99
IB	PF3D7_0623900	=	1.02	0.99
IB	PF3D7_1013500	=	0.64	0.97

FADE	PF3D7_0811300	=	0.02	0.98
FADE	PF3D7_1305500	=	0.63	0.99
FADE	PF3D7_0615900	=	0.31	0.95
FADE	PF3D7_1122900	=	0.41	0.88
FADE	PF3D7_1141900	=	1.83	0.97
FADE	PF3D7_1475600	=	0.26	0.95
FADE	PF3D7_0211100	=	0.15	0.90
FADE	PF3D7_0203000	=	0.23	0.92
FADE	PF3D7_0404600	=	1.07	0.99
FADE	PF3D7_0416500	=	0.76	0.93

FADE	PF3D7_1018400	=	2.85	0.98
FADE	PF3D7_0511500	=	0.00	0.45
FADE	PF3D7_1360200	=	2.79	0.94
FADE	PF3D7_0611800	=	1.35	0.80
FADE	PF3D7_0619900	=	2.48	0.97
FADE	PF3D7_0521900	=	6.10	0.91
FADE	PF3D7_1219300	=	5.02	0.96
FADE	PF3D7_1133700	=	2.77	0.98
FADE	PF3D7_0808700	=	6.82	0.97
FADE	PF3D7_1136000	=	3.59	0.98
FADE	PF3D7_0931000	=	1.55	0.94
FADE	PF3D7_0830900	=	1.29	0.97
FADE	PF3D7_1231800	=	6.04	0.94
FADE	PF3D7_1035800	=	5.59	0.97
FADE	PF3D7_1444100	=	13.55	0.99
FADE	PF3D7_1138900	=	2.33	0.98
FADE	PF3D7_1441800	=	0.83	0.97
FADE	PF3D7_1345800	=	0.94	0.94
FADE	PF3D7_1432400	=	2.38	0.99
FADE	PF3D7_0819800	=	0.99	0.98
FADE	PF3D7_1207800	=	0.33	0.83
FADE	PF3D7_1143500	=	1.26	0.96
FADE	PF3D7_1001600	=	0.40	0.88
FADE	PF3D7_1019400	=	0.97	0.99
FADE	PF3D7_1311900	=	0.67	0.93
FADE	PF3D7_1035700	=	0.56	0.91
FADE	PF3D7_1103100	=	0.68	0.99
FADE	PF3D7_0812500	=	0.41	0.98
FADE	PF3D7_1216100	=	1.11	0.99
FADE	PF3D7_0206100	=	0.30	0.98
FADE	PF3D7_1352500	=	0.33	0.98
FADE	PF3D7_1226900	=	0.52	0.87
FADE	PF3D7_1368800	=	0.11	0.99
FADE	PF3D7_1432400	=	1.61	0.99
FADE	PF3D7_1343900	=	0.75	0.98
FADE	PF3D7_1328800	=	0.54	0.96
FADE	PF3D7_1138700	=	0.02	0.97
FADE	PF3D7_1327100	=	0.02	0.96
FADE	PF3D7_1136600	=	0.01	0.92
FADE	PF3D7_0902200	=	0.02	0.96
FADE	PF3D7_0727100	=	0.02	0.96
FADE	PF3D7_0105500	=	0.04	0.97
FADE	PF3D7_1126100	=	0.03	0.97
FADE	PF3D7_1343700	=	0.03	0.97
FADE	PF3D7_1330500	=	8.78	0.99
FADE	PF3D7_1343000	=	28.94	0.99
FADE	PF3D7_1302000	=	4.82	0.97
FADE	PF3D7_1107900	=	5.72	0.97
FADE	PF3D7_0606000	=	13.88	0.99
FADE	PF3D7_0614500	=	3.65	0.98
FADE	PF3D7_0203700	=	2.87	0.96
FADE	PF3D7_0601700	=	6.03	0.99

FADE	PF3D7_1001900	=	1.49	0.98
FADE	PF3D7_1409500	=	0.86	0.97
FADE	PF3D7_0916700	=	1.05	0.94
FADE	PF3D7_0402000	=	0.66	0.99
FADE	PF3D7_0102600	=	6.24	0.93
FADE	PF3D7_1433400	=	1.82	0.96
FADE	PF3D7_0322000	=	1.83	0.95
FADE	PF3D7_0623900	=	1.69	0.97
FADE	PF3D7_1013500	=	0.42	0.99

TLM	PF3D7_0811300	=	0.71	0.96
TLM	PF3D7_1305500	=	0.84	0.97
TLM	PF3D7_0615900	=	0.79	0.93
TLM	PF3D7_1122900	=	1.23	0.90
TLM	PF3D7_1141900	=	1.21	0.95
TLM	PF3D7_1475600	=	0.44	0.93
TLM	PF3D7_0211100	=	0.38	0.95
TLM	PF3D7_0203000	=	0.44	0.97
TLM	PF3D7_0404600	=	1.07	0.99
TLM	PF3D7_0416500	=	1.12	0.96
TLM	PF3D7_1018400	>	475.7 uM	
TLM	PF3D7_0511500	=	0.00	0.74
TLM	PF3D7_1360200	=	1.66	0.96
TLM	PF3D7_0611800	=	1.45	0.95
TLM	PF3D7_0619900	=	0.90	0.99
TLM	PF3D7_0521900	=	2.12	0.99
TLM	PF3D7_1219300	=	2.23	0.99
TLM	PF3D7_1133700	=	63.36	0.91
TLM	PF3D7_0808700	=	1.65	0.99
TLM	PF3D7_1136000	=	1.83	1.00
TLM	PF3D7_0931000	=	1.71	0.99
TLM	PF3D7_0830900	=	0.82	0.96
TLM	PF3D7_1231800	=	2.16	0.99
TLM	PF3D7_1035800	=	1.74	0.98
TLM	PF3D7_1444100	=	2.51	0.98
TLM	PF3D7_0203600	=	0.93	0.96
TLM	PF3D7_1138900	=	1.32	0.98
TLM	PF3D7_1441800	=	0.54	0.95
TLM	PF3D7_1345800	=	0.56	0.94
TLM	PF3D7_1432400	=	1.23	0.99
TLM	PF3D7_0819800	=	0.54	0.99
TLM	PF3D7_1207800	=	0.31	0.94
TLM	PF3D7_1143500	=	1.53	0.94
TLM	PF3D7_1001600	=	1364.83	0.94
TLM	PF3D7_1019400	=	0.42	0.96
TLM	PF3D7_1311900	=	0.69	0.96
TLM	PF3D7_1035700	=	0.58	0.99
TLM	PF3D7_1103100	>	475.70	
TLM	PF3D7_0812500	=	0.63	0.93
TLM	PF3D7_1216100	=	0.65	0.99

TLM	PF3D7_0206100	=	0.87	0.96
TLM	PF3D7_1352500	=	0.33	1.00
TLM	PF3D7_1226900	=	0.81	0.95
TLM	PF3D7_1368800	=	0.12	0.96
TLM	PF3D7_1432400	=	0.82	0.99
TLM	PF3D7_1343900	>	475.70	
TLM	PF3D7_1328800	=	2.33	0.89
TLM	PF3D7_1138700	=	0.76	0.98
TLM	PF3D7_1327100	=	0.42	0.99
TLM	PF3D7_1136600	=	0.39	0.98
TLM	PF3D7_0902200	=	0.48	0.98
TLM	PF3D7_0727100	=	0.22	0.99
TLM	PF3D7_0105500	=	0.43	0.98
TLM	PF3D7_1126100	=	0.26	0.97
TLM	PF3D7_1343700	=	0.22	0.99
TLM	PF3D7_1330500	=	1.37	0.99
TLM	PF3D7_1343000	=	3.05	0.99
TLM	PF3D7_1302000	=	3.11	0.98
TLM	PF3D7_0203700	=	0.43	0.99
TLM	PF3D7_1107900	=	1.33	0.97
TLM	PF3D7_0606000	=	1.60	0.98
TLM	PF3D7_0614500	=	1.18	0.98
TLM	PF3D7_0203700	=	0.97	0.99
TLM	PF3D7_0601700	=	0.90	0.98

DAN	PF3D7_0811300	=	5.00	0.97
DAN	PF3D7_1305500	=	1.30	0.97
DAN	PF3D7_0615900	=	1.27	0.85
DAN	PF3D7_1122900	=	1.70	0.94
DAN	PF3D7_1141900	=	1.94	0.90
DAN	PF3D7_1475600	=	0.30	0.90
DAN	PF3D7_0211100	=	1.20	0.91
DAN	PF3D7_0203000	=	1.27	0.99
DAN	PF3D7_0404600	=	1.76	0.92
DAN	PF3D7_0416500	=	1.21	0.98
DAN	PF3D7_1018400	=	1.99	0.99
DAN	PF3D7_0511500	=	1.61	1.00
DAN	PF3D7_1360200	=	1.84	0.97
DAN	PF3D7_0611800	=	1.79	0.94
DAN	PF3D7_0619900	=	1.12	0.98
DAN	PF3D7_0521900	=	3.17	1.00
DAN	PF3D7_1219300	=	2.19	0.99
DAN	PF3D7_1133700	=	1.37	0.99
DAN	PF3D7_0808700	=	1.06	0.93
DAN	PF3D7_1136000	=	1.27	0.98
DAN	PF3D7_0931000	=	1.14	0.97
DAN	PF3D7_0830900	=	0.84	0.84
DAN	PF3D7_1231800	=	1.19	0.98
DAN	PF3D7_1035800	=	1.11	0.95
DAN	PF3D7_1444100	=	1.13	0.98

DAN	PF3D7_1138900	=	1.06	0.94
DAN	PF3D7_1441800	=	0.98	0.96
DAN	PF3D7_1345800	=	0.96	0.99
DAN	PF3D7_1432400	=	1.33	1.00
DAN	PF3D7_0819800	=	0.81	0.97
DAN	PF3D7_1207800	=	0.76	0.94
DAN	PF3D7_1143500	=	0.50	0.98
DAN	PF3D7_1001600	=	1.81	0.85
DAN	PF3D7_1019400	=	1.11	0.96
DAN	PF3D7_1311900	=	0.60	0.94
DAN	PF3D7_1035700	=	0.94	0.95
DAN	PF3D7_1103100	=	0.84	0.98
DAN	PF3D7_0812500	=	0.95	0.92

TABLE S4

SUMMARY OF HIGH-THROUGHPUT FORWARD GENETICS SCREEN

The Wild Type values are the Mean±SD Growth Inhibition 50% (GI50) from all batches represented as uM units.

Inhibitor	Column1	GI50 (Mean±SD)	R^2
AL	=	33.62±6.8 (n=8)	1.00
ALLO	=	610.09±82.7 (n=8)	0.81
AM	=	0.6±0.07 (n=8)	0.99
AQ	=	22.63±8.3 (n=8)	0.99
AS	=	0.94±0.03 (n=8)	0.99
ATQ	=	0.42±0.001 (n=8)	0.99
BZD	=	3684.7±536.2 (n=8)	0.91
C-MIR	=	186.01±37.9 (n=8)	0.91
CG	=	0.01±0.0008 (n=8)	0.99
CQ	=	0.03±0.009 (n=8)	0.99
CRL	=	17.7±2.54 (n=8)	0.98
CUR	=	20.97±0.6 (n=8)	0.91
CYC	=	158.96±32.65 (n=8)	0.97
CYTD	=	0.10±0.03 (n=8)	0.99
DAN	=	40.48±18.3 (n=8)	0.92
DHA	=	0.01±0.005 (n=8)	1.00
DNR	=	3.5±0.9 (n=8)	0.99
DNTA	=	8.14±2.6 (n=8)	0.91
E-64	=	0.61±0.23 (n=8)	0.99
E-PPMP	=	15.26±6.29 (n=8)	0.99
EHNA	=	134.8±5.1 (n=8)	0.99
FADE	=	1.8±0.3 (n=8)	0.93
IB	=	52.6±13.9 (n=8)	0.91
LUMF	=	10.6±2.8 (n=8)	0.95
MFQ	=	10.5±3.2 (n=8)	1.00
MTX	=	0.016±0.001 (n=8)	1.00
MZB	=	192.93±27.42 (n=8)	0.98
PEP-A	=	33.18±16.8 (n=8)	0.98
PHEN	=	0.84±0.07 (n=8)	0.89
PIP	=	4115.11±1119.6 (n=8)	0.98
PQ	=	3.51±1.9 (n=10)	0.99
PYR	=	0.05±0.007 (n=8)	0.99
QHS	=	0.04±0.008 (n=8)	0.99
ROT	=	51.36±13.8 (n=8)	0.97
SDZ	=	874.6+92.7 (n=8)	0.98
SF	=	1.1+0.27 (n=8)	0.99
SP	=	4589.2±893.6 (n=8)	0.97
T-MIR	=	297.4±65.1 (n=8)	0.96
T-PPMP	=	1.16±0.03 (n=8)	0.99
TCPA	=	250.9+97.43 (n=8)	0.99
TLM	=	134.27±62.92 (n=8)	0.99
TOFA	=	75.34±12.8 (n=8)	0.98
TRI	=	20.29±5.61 (n=8)	0.97
TSA	=	0.03+0.01 (n=8)	0.99
TTFA	=	8.3±1.9 (n=8)	0.92
VER	=	3.72±1.4 (n=8)	0.99

TABLE S5

Drug1	Drug2	Correlation	Number of Permutation	SamePathway?
C-MIR	T-MIR	0.8369467	0	Yes
DHA	QHS	0.8201761	0	Yes
CG	MTX	0.7811595	0	Yes
CG	PYR	0.692375	0	Yes
AM	AS	0.6910947	0	Yes
PEP-A	PQ	0.6465371	0	Yes
FADE	SF	0.6357308	0	Yes
AM	AL	0.6265279	0	Yes
AQ	PIP	0.6140244	0	Yes
CRL	TLM	0.5807819	0	Yes
AS	AL	0.5648325	0	Yes
MTX	PYR	0.5403937	0	Yes
AQ	MFQ	0.5214111	0	Yes
E-64	PQ	0.516537	0	Yes
E-64	PEP-A	0.5099284	0	Yes
CQ	VER	0.4964025	0	Yes
CQ	PEP-A	0.4951855	0	Yes
ALLO	EHNA	0.4878488	0	Yes
CUR	TSA	0.4631567	0	Yes
MFQ	PIP	0.444929	0	Yes
ATQ	ROT	0.4377922	0	Yes
ALLO	MZB	0.4260388	0	Yes
AQ	PEP-A	0.4210027	0	Yes
CQ	E-64	0.4188448	0	Yes
PEP-A	PIP	0.4171183	0	Yes
ATQ	TTFA	0.4089251	0	Yes
TCPA	TLM	0.407226	0	Yes
TOFA	TRI	0.3955533	0	Yes
CRL	TOFA	0.3878575	0	Yes
PIP	PQ	0.3671882	0	Yes
AQ	PQ	0.3571045	3	Yes
CQ	PIP	0.3564938	2	Yes
CRL	TRI	0.3558507	3	Yes
CQ	PQ	0.346782	2	Yes
BZD	DNTA	0.3332943	1	Yes
CRL	TCPA	0.328903	1	Yes
AM	QHS	0.2768355	9	Yes
TLM	TOFA	0.2543435	27	Yes
BZD	CYTD	0.2533785	22	Yes
MFQ	PEP-A	0.2494083	22	Yes
CYC	T-MIR	0.2341463	31	Yes
CYC	E-PPMP	0.2189928	58	Yes
AQ	CQ	0.2125273	41	Yes
TLM	TRI	0.2051632	77	Yes
MFQ	PQ	0.2015263	63	Yes
AM	DHA	0.1971373	74	Yes

AQ	E-64	0.1934744	72	Yes
AL	QHS	0.1862745	101	Yes
E-PPMP	T-PPMP	0.17744	120	Yes
AL	DHA	0.1599851	135	Yes
CQ	MFQ	-0.003362	968	Yes
PYR	SP	-0.045484	700	Yes
TCPA	TOFA	-0.102755	371	Yes
PHEN	ROT	-0.140313	255	Yes
CG	SDZ	-0.154254	215	Yes
TCPA	TRI	-0.178942	104	Yes
MTX	SDZ	-0.284935	9	Yes
CG	SP	-0.314556	7	Yes
MTX	SP	-0.363091	2	Yes
MZB	VER	0.8368837	0	No
CRL	TSA	0.8343491	0	No
E-PPMP	TOFA	0.8084237	0	No
CRL	FADE	0.7923113	0	No
DAN	IB	0.7885026	0	No
BZD	VER	0.7767861	0	No
FADE	TSA	0.755663	0	No
DAN	QHS	0.7540454	0	No
SDZ	TOFA	0.7503743	0	No
PQ	SF	0.735329	0	No
CYTD	E-PPMP	0.7347192	0	No
BZD	MZB	0.7335443	0	No
CYTD	TOFA	0.7289716	0	No
BZD	DHA	0.7207736	0	No
DAN	PEP-A	0.7206721	0	No
CRL	VER	0.7149245	0	No
PEP-A	QHS	0.7095689	0	No
AM	AQ	0.7093496	0	No
CRL	MZB	0.695804	0	No
E-64	FADE	0.692732	0	No
CYTD	T-MIR	0.6907824	0	No
FADE	TTFA	0.6904662	0	No
E-PPMP	T-MIR	0.6894535	0	No
DHA	PQ	0.6826046	0	No
MZB	PQ	0.6792721	0	No
DNTA	ROT	0.6775938	0	No
DHA	MZB	0.6753282	0	No
DHA	VER	0.6742987	0	No
BZD	CRL	0.6740019	0	No
ALLO	TLM	0.6736943	0	No
E-PPMP	SDZ	0.6716296	0	No
ALLO	FADE	0.6681572	0	No
AQ	LUMF	0.6674609	0	No
ALLO	CRL	0.6665495	0	No
DHA	SF	0.6627349	0	No

FADE	TLM	0.6603972	0	No
IB	QHS	0.6572416	0	No
BZD	FADE	0.6566104	0	No
CYC	EHNA	0.6530137	0	No
CRL	DHA	0.6525773	0	No
PEP-A	SF	0.652262	0	No
SF	TTFA	0.6513548	0	No
BZD	SF	0.6468041	0	No
AM	LUMF	0.6452837	0	No
DHA	PEP-A	0.642184	0	No
DHA	FADE	0.6417323	0	No
FADE	VER	0.6402927	0	No
CRL	TTFA	0.6365054	0	No
TTFA	TLM	0.6344263	0	No
CYC	TTFA	0.6343003	0	No
DHA	E-64	0.6337406	0	No
PQ	VER	0.6330921	0	No
CRL	E-64	0.6320478	0	No
FADE	QHS	0.6312247	0	No
CUR	SDZ	0.6254527	0	No
AM	MFQ	0.6239784	0	No
DAN	LUMF	0.6206293	0	No
FADE	MZB	0.6205059	0	No
SP	T-MIR	0.6179348	0	No
E-64	TSA	0.617898	0	No
C-MIR	SP	0.6163427	0	No
IB	PEP-A	0.6163053	0	No
ALLO	E-64	0.6160717	0	No
QHS	SF	0.6152161	0	No
C-MIR	LUMF	0.6148635	0	No
T-MIR	TOFA	0.6145438	0	No
E-64	TLM	0.6139849	0	No
E-64	SF	0.6137829	0	No
ATQ	LUMF	0.6129438	0	No
EHNA	TTFA	0.6084963	0	No
QHS	TTFA	0.6073151	0	No
DHA	TSA	0.604367	0	No
PEP-A	VER	0.6002066	0	No
ALLO	TSA	0.5977326	0	No
BZD	PQ	0.5974484	0	No
BZD	E-64	0.5969093	0	No
SF	VER	0.5966531	0	No
CRL	CYC	0.5966479	0	No
DAN	SF	0.5964024	0	No
BZD	TSA	0.5962165	0	No
AL	C-MIR	0.5949672	0	No
CRL	PEP-A	0.5928739	0	No
TTFA	TSA	0.591252	0	No

LUMF	PEP-A	0.5890616	0	No
C-MIR	E-PPMP	0.5889792	0	No
CUR	TOFA	0.5887341	0	No
CYC	FADE	0.5854562	0	No
CUR	E-PPMP	0.5849401	0	No
AL	T-MIR	0.5823655	0	No
CRL	SF	0.5823639	0	No
QHS	VER	0.5805494	0	No
LUMF	MFQ	0.5802332	0	No
FADE	PEP-A	0.578907	0	No
PEP-A	TTFA	0.5778633	0	No
FADE	PQ	0.5775439	0	No
CYC	E-64	0.5771035	0	No
C-MIR	PIP	0.5743902	0	No
EHNA	PHEN	0.5740483	0	No
BZD	PEP-A	0.5711674	0	No
BZD	CQ	0.570494	0	No
SP	TRI	0.5704615	0	No
EHNA	FADE	0.5697664	0	No
LUMF	T-MIR	0.5674264	0	No
CRL	QHS	0.5660001	0	No
AS	MFQ	0.5651457	0	No
E-64	TTFA	0.563108	0	No
E-64	QHS	0.5617819	0	No
CRL	PQ	0.561173	0	No
ATQ	C-MIR	0.5605917	0	No
BZD	QHS	0.5600225	0	No
TSA	VER	0.5593993	0	No
ATQ	DNTA	0.5576797	0	No
ALLO	DHA	0.5574207	0	No
PQ	QHS	0.5572312	0	No
CRL	EHNA	0.5571063	0	No
C-MIR	CYTD	0.5569394	0	No
MZB	SF	0.5563943	0	No
AM	C-MIR	0.5554201	0	No
AQ	C-MIR	0.5547877	0	No
DHA	TLM	0.5546826	0	No
AM	T-MIR	0.5500678	0	No
DAN	TTFA	0.5491486	0	No
AS	T-MIR	0.5478125	0	No
AQ	DAN	0.5462686	0	No
LUMF	QHS	0.54586	0	No
AS	SP	0.5456475	0	No
MZB	TSA	0.5452811	0	No
CQ	QHS	0.5433711	0	No
PQ	TTFA	0.5420936	0	No
C-MIR	TOFA	0.5411472	0	No
ALLO	CYC	0.540131	0	No

TTFA	VER	0.5398804	0	No
LUMF	PQ	0.53789	0	No
DHA	TTFA	0.5373935	0	No
EHNA	TCPA	0.5367684	0	No
AM	ROT	0.5364168	0	No
CYC	VER	0.5360464	0	No
QHS	TSA	0.5341592	0	No
LUMF	PIP	0.5341155	0	No
CYC	TLM	0.5335141	0	No
MZB	PEP-A	0.532972	0	No
ALLO	QHS	0.532442	0	No
C-MIR	FADE	0.5309395	0	No
CYC	TSA	0.5299126	0	No
LUMF	TTFA	0.5290514	0	No
CYC	QHS	0.5271348	0	No
EHNA	VER	0.5266602	0	No
SDZ	TSA	0.5249928	0	No
ALLO	BZD	0.5232302	0	No
LUMF	SF	0.522612	0	No
AS	TOFA	0.5204862	0	No
TLM	TSA	0.5192728	0	No
PHEN	TLM	0.5186996	0	No
AM	SP	0.5180924	0	No
CUR	TRI	0.5177226	0	No
CRL	PHEN	0.5174875	0	No
CYC	DHA	0.5173654	0	No
CQ	FADE	0.5161152	0	No
SF	TSA	0.5156988	0	No
IB	TTFA	0.5155546	0	No
TLM	VER	0.5147451	0	No
CYC	MZB	0.5140244	0	No
CYC	SF	0.5128078	0	No
AS	PIP	0.51274	0	No
BZD	CYC	0.5122832	0	No
DAN	VER	0.5113964	0	No
MZB	QHS	0.5102645	0	No
LUMF	SP	0.5093441	0	No
C-MIR	ROT	0.5093385	0	No
C-MIR	TRI	0.5083136	0	No
ALLO	TTFA	0.5076729	0	No
PEP-A	TSA	0.5074189	0	No
AS	LUMF	0.5063178	0	No
SF	TLM	0.5063118	0	No
AS	C-MIR	0.5059423	0	No
C-MIR	SF	0.5054342	0	No
AQ	SP	0.5048809	0	No
ATQ	CRL	0.5047351	0	No
CYC	IB	0.5039973	0	No

LUMF	ROT	0.5039776	0	No
AS	ATQ	0.5036049	0	No
CYTD	SDZ	0.5023877	0	No
QHS	TLM	0.5017934	0	No
E-64	VER	0.501643	0	No
BZD	TTFA	0.4991708	0	No
CUR	CYTD	0.4989188	0	No
AS	TRI	0.4985264	0	No
ATQ	T-MIR	0.4963866	0	No
PHEN	TSA	0.4962986	0	No
AL	TRI	0.4953534	0	No
BZD	EHNA	0.4951407	0	No
FADE	PHEN	0.4939302	0	No
PQ	TSA	0.4937583	0	No
DAN	FADE	0.4936483	0	No
AS	E-PPMP	0.4908113	0	No
AM	ATQ	0.4906278	0	No
ALLO	VER	0.4900095	0	No
DAN	DHA	0.4885406	0	No
IB	SF	0.4883948	0	No
ROT	T-MIR	0.4881095	0	No
IB	VER	0.4870624	0	No
AL	MFQ	0.4866184	0	No
PHEN	TCPA	0.485882	0	No
SP	TOFA	0.4855041	0	No
ATQ	SP	0.4854476	0	No
FADE	T-MIR	0.4852529	0	No
PQ	TLM	0.4844413	0	No
ATQ	PEP-A	0.4836947	0	No
BZD	TLM	0.4831567	0	No
DAN	PQ	0.4826753	0	No
ATQ	BZD	0.4822785	0	No
CRL	T-PPMP	0.4819766	0	No
DHA	IB	0.4817707	0	No
CRL	SDZ	0.4805461	0	No
CQ	MZB	0.4794628	0	No
E-PPMP	SP	0.4792484	0	No
CQ	TSA	0.4785023	0	No
CYC	PEP-A	0.4781617	0	No
EHNA	MZB	0.4780859	0	No
AS	ROT	0.4773848	0	No
MZB	TLM	0.4771761	0	No
FADE	SDZ	0.4756125	0	No
DHA	ROT	0.4755479	0	No
AL	ROT	0.4754538	0	No
CYTD	SP	0.4736982	0	No
EHNA	TLM	0.4731869	0	No
AQ	AS	0.4731847	0	No

AM	PIP	0.4729675	0	No
DAN	MFQ	0.4710196	0	No
IB	LUMF	0.4704295	0	No
DNTA	TOFA	0.4696583	0	No
AQ	ATQ	0.4687444	0	No
PIP	TOFA	0.4685411	0	No
DNTA	E-PPMP	0.4684726	0	No
CYTD	FADE	0.4683318	0	No
AS	CYTD	0.4681199	0	No
CQ	DHA	0.4674676	0	No
ALLO	PQ	0.4632027	0	No
PEP-A	TLM	0.4621993	0	No
AL	SP	0.4620535	0	No
AQ	QHS	0.4617539	0	No
ALLO	SF	0.4612153	0	No
PIP	TRI	0.4604362	0	No
DHA	T-PPMP	0.4581037	0	No
E-PPMP	ROT	0.4568757	0	No
CYC	DAN	0.4549961	0	No
ATQ	FADE	0.4545619	0	No
FADE	LUMF	0.4541015	0	No
AL	E-PPMP	0.4538982	0	No
BZD	IB	0.4522269	1	No
BZD	C-MIR	0.4521331	0	No
AL	LUMF	0.4518795	0	No
ATQ	CYTD	0.4513262	0	No
DAN	PIP	0.4505923	1	No
C-MIR	PEP-A	0.4500452	0	No
C-MIR	DNTA	0.4499989	0	No
PIP	T-MIR	0.4499322	0	No
MZB	TTFA	0.4496324	0	No
ATQ	TRI	0.4490766	0	No
CRL	CUR	0.4480453	0	No
AQ	AL	0.4474732	0	No
CUR	SP	0.4471144	0	No
CYC	PQ	0.4467279	0	No
ATQ	MZB	0.445551	0	No
DHA	DNTA	0.4446389	0	No
FADE	PIP	0.4434282	0	No
LUMF	TRI	0.4430493	0	No
ATQ	TSA	0.4403442	0	No
C-MIR	TSA	0.4398135	0	No
E-64	ROT	0.4374785	0	No
DIA	TLM	0.4364082	0	No
SF	T-MIR	0.4362491	0	No
E-PPMP	TRI	0.4354586	0	No
DAN	TSA	0.4345111	0	No
ATQ	VER	0.4340133	0	No

DNTA	LUMF	0.4338481	0	No
ALLO	PEP-A	0.4336269	0	No
PHEN	TTFA	0.433622	0	No
PIP	TSA	0.433456	0	No
LUMF	TOFA	0.4326244	0	No
AQ	T-MIR	0.4317525	0	No
AQ	TSA	0.4314799	0	No
CUR	FADE	0.4308516	0	No
C-MIR	SDZ	0.4307496	0	No
PIP	SP	0.4307048	1	No
E-64	E-PPMP	0.430637	0	No
CUR	MZB	0.4303434	0	No
QHS	T-PPMP	0.430037	0	No
EHNA	TSA	0.4291638	0	No
CUR	DNTA	0.4284561	0	No
E-64	MZB	0.427927	0	No
E-PPMP	FADE	0.4273713	0	No
ROT	TRI	0.4273269	0	No
C-MIR	MFQ	0.4268083	0	No
AS	DNTA	0.4263783	0	No
E-PPMP	SF	0.4252621	0	No
MFQ	T-MIR	0.4243907	0	No
CRL	CQ	0.4242829	0	No
ROT	TOFA	0.4236771	0	No
ROT	SP	0.4236231	0	No
AL	PIP	0.4233651	0	No
CRL	DAN	0.4230828	0	No
IB	PQ	0.4225747	0	No
C-MIR	DHA	0.4219531	1	No
C-MIR	TTFA	0.4218882	1	No
PQ	SDZ	0.4198061	0	No
C-MIR	QHS	0.4190248	0	No
PIP	VER	0.4184757	0	No
PIP	QHS	0.4182795	0	No
C-MIR	CRL	0.4181435	0	No
TOFA	TSA	0.4169019	0	No
IB	MZB	0.4156504	0	No
ALLO	PIP	0.4147245	0	No
AQ	SDZ	0.4129405	0	No
FADE	TOFA	0.4119467	0	No
FADE	IB	0.4116305	0	No
CYTD	TTFA	0.4073263	1	No
ALLO	TCPA	0.4072042	0	No
EHNA	SF	0.4071245	2	No
SP	SDZ	0.4064786	0	No
LUMF	TSA	0.4062219	2	No
DNTA	SDZ	0.4053184	0	No
DHA	LUMF	0.4033901	0	No

PEP-A	T-MIR	0.4019648	0	No
C-MIR	CUR	0.4014928	0	No
SDZ	T-MIR	0.4013494	1	No
C-MIR	TLM	0.4013122	0	No
ATQ	TOFA	0.4005194	1	No
MZB	SDZ	0.4003639	1	No
CUR	TTFA	0.3993123	1	No
ATQ	CUR	0.3991441	0	No
CYTD	DNTA	0.3990537	0	No
DNTA	T-PPMP	0.3978319	0	No
E-PPMP	TSA	0.3969715	0	No
MFQ	TRI	0.3968504	0	No
T-MIR	TTFA	0.3962104	0	No
CYC	PHEN	0.3953722	1	No
CYTD	DIA	0.3950529	1	No
CYTD	LUMF	0.3948576	0	No
MFQ	SP	0.3948508	0	No
C-MIR	CQ	0.3942079	0	No
ATQ	PIP	0.3941509	0	No
E-64	T-MIR	0.393261	1	No
CQ	DAN	0.3930038	1	No
AQ	IB	0.3927733	0	No
CRL	PIP	0.391233	0	No
ROT	SDZ	0.391011	0	No
MZB	T-PPMP	0.3909349	0	No
PIP	ROT	0.3893719	0	No
T-PPMP	TSA	0.3889676	0	No
PEP-A	T-PPMP	0.388654	2	No
CUR	DIA	0.3880534	0	No
CUR	SF	0.3875213	0	No
CQ	TRI	0.3871959	2	No
PIP	SDZ	0.387142	0	No
DIA	TOFA	0.3863366	2	No
PEP-A	SP	0.3862036	0	No
CUR	DHA	0.3859201	0	No
AQ	SF	0.3857745	0	No
BZD	SDZ	0.3852458	1	No
PQ	T-PPMP	0.3851782	1	No
E-64	IB	0.3850406	0	No
C-MIR	PQ	0.3848599	0	No
AQ	CRL	0.3842475	1	No
DIA	DNTA	0.3810275	1	No
PQ	ROT	0.3805324	1	No
CQ	IB	0.3804506	1	No
ATQ	QHS	0.3801576	1	No
PIP	TTFA	0.3800404	1	No
CRL	LUMF	0.3773815	0	No
CRL	CYTD	0.3772665	0	No

DHA	DIA	0.3767768	0	No
DNTA	T-MIR	0.37677	0	No
ALLO	DAN	0.3764722	2	No
ATQ	DAN	0.3762351	1	No
CUR	QHS	0.3761963	0	No
GYLP	PHEN	0.3761643	4	No
AQ	TOFA	0.3747516	0	No
DIA	T-PPMP	0.3745648	3	No
ATQ	PQ	0.3744149	0	No
E-PPMP	LUMF	0.3731503	2	No
CYC	T-PPMP	0.3727889	1	No
T-MIR	TRI	0.3727099	2	No
ATQ	E-PPMP	0.3724255	0	No
E-64	LUMF	0.3721248	1	No
ATQ	E-64	0.3719646	1	No
DNTA	TRI	0.3714141	0	No
E-64	SDZ	0.3710832	1	No
IB	T-PPMP	0.3707903	0	No
DAN	E-64	0.3707493	1	No
ALLO	T-PPMP	0.3706999	1	No
ATQ	SF	0.3704176	1	No
CUR	T-PPMP	0.369426	1	No
QHS	ROT	0.3680609	0	No
AQ	VER	0.3679108	1	No
BZD	DAN	0.3671259	0	No
T-PPMP	TLM	0.3665503	4	No
CYTD	PIP	0.3660159	0	No
ATQ	CYC	0.3656504	0	No
ATQ	DHA	0.3637164	1	No
EHNA	PEP-A	0.3622255	1	No
SDZ	TRI	0.3613104	1	No
ALLO	CQ	0.3609553	1	No
AQ	DHA	0.3601931	2	No
DAN	MZB	0.3594327	3	No
DHA	E-PPMP	0.3576861	0	No
ATQ	SDZ	0.3572608	2	No
T-PPMP	TTFA	0.357165	0	No
C-MIR	VER	0.3570594	0	No
CRL	IB	0.3564229	2	No
DNTA	MZB	0.3562185	2	No
AM	DAN	0.3561355	0	No
ROT	SF	0.3558592	0	No
AL	ATQ	0.3552979	0	No
E-64	T-PPMP	0.3551418	3	No
C-MIR	E-64	0.3539202	1	No
CYTD	TSA	0.3533826	0	No
E-PPMP	PQ	0.3531072	2	No
DHA	SDZ	0.3524285	3	No

AQ	ROT	0.3521082	2	No
QHS	SP	0.3519295	0	No
CYTD	PEP-A	0.35163	2	No
SF	SDZ	0.3506205	4	No
E-64	EHNA	0.3504711	4	No
IB	TSA	0.3501434	2	No
AQ	FADE	0.3500226	1	No
C-MIR	DAN	0.349349	3	No
DNTA	PQ	0.3482144	0	No
IB	PIP	0.3471319	0	No
SDZ	T-PPMP	0.3468331	0	No
SF	TRI	0.3467067	0	No
SP	TTFA	0.3463586	1	No
CQ	SF	0.3456356	2	No
BZD	E-PPMP	0.3456165	2	No
CRL	DNTA	0.3451792	3	No
CQ	CUR	0.343834	3	No
CUR	LUMF	0.3435337	3	No
DIA	E-PPMP	0.3430822	6	No
E-PPMP	TTFA	0.3425174	2	No
E-64	TOFA	0.3424929	4	No
AQ	TRI	0.3417363	3	No
ALLO	LUMF	0.3414202	3	No
E-64	PHEN	0.341281	4	No
QHS	TRI	0.3393953	2	No
DIA	PHEN	0.3392533	8	No
DIA	E-64	0.3386194	3	No
CUR	PQ	0.3381813	3	No
SDZ	VER	0.3377506	4	No
CYC	TCPA	0.337421	0	No
T-PPMP	TOFA	0.3362937	1	No
CRL	E-PPMP	0.3362869	2	No
TSA	TRI	0.3355221	4	No
TTFA	TRI	0.3354167	1	No
T-MIR	TSA	0.3344701	0	No
FADE	TRI	0.3310656	4	No
DHA	TOFA	0.3306513	2	No
PQ	TOFA	0.329688	2	No
BZD	PIP	0.3290202	1	No
TTFA	TOFA	0.3288542	3	No
T-MIR	TLM	0.3281778	5	No
SP	SF	0.3276799	1	No
DHA	PIP	0.3269134	2	No
ATQ	CQ	0.3263248	5	No
PEP-A	TRI	0.3261762	6	No
DAN	EHNA	0.3259964	8	No
AQ	BZD	0.3256212	5	No
LUMF	TLM	0.3250313	9	No

T-PPMP	VER	0.3246649	3	No
EHNA	QHS	0.3232551	5	No
MFQ	QHS	0.3229378	7	No
DNTA	E-64	0.322762	4	No
DIA	SDZ	0.3213199	4	No
ATQ	MFQ	0.3209648	7	No
PQ	TRI	0.3207805	5	No
CUR	T-MIR	0.319322	4	No
LUMF	T-PPMP	0.3181664	2	No
EHNA	IB	0.31782	6	No
SDZ	TTFA	0.3177279	3	No
E-64	TRI	0.31728	5	No
DNTA	SP	0.3171329	3	No
DIA	MZB	0.3169619	2	No
SP	TSA	0.3167321	2	No
CRL	SP	0.3166451	3	No
AS	SDZ	0.3166115	5	No
AM	TRI	0.315607	5	No
CYTD	TRI	0.3153509	7	No
CYTD	E-64	0.3146056	12	No
CYC	LUMF	0.3143536	4	No
DAN	SP	0.3143233	6	No
ATQ	IB	0.3140018	3	No
CQ	TLM	0.3128507	18	No
SF	TOFA	0.3126373	5	No
CYTD	TLM	0.3124688	10	No
DAN	T-MIR	0.3122777	4	No
BZD	CUR	0.3122128	6	No
ATQ	T-PPMP	0.3121404	2	No
LUMF	SDZ	0.3103349	9	No
ALLO	ATQ	0.3101174	4	No
AM	TOFA	0.3075881	9	No
ALLO	PHEN	0.3061704	11	No
CYTD	T-PPMP	0.3058317	5	No
AM	SF	0.3057267	5	No
CUR	VER	0.3045258	7	No
SDZ	TLM	0.3045148	6	No
DAN	TLM	0.3044931	4	No
CQ	TTFA	0.3044384	7	No
BZD	T-MIR	0.3034459	9	No
PQ	T-MIR	0.3032086	9	No
DAN	T-PPMP	0.3031555	7	No
DHA	EHNA	0.3028277	5	No
LUMF	VER	0.3027586	11	No
IB	TLM	0.3027032	14	No
BZD	ROT	0.3024684	8	No
AM	E-PPMP	0.3019648	8	No
CYTD	SF	0.30184	7	No

QHS	T-MIR	0.3016554	7	No
FADE	T-PPMP	0.3013567	6	No
CYC	PIP	0.3010614	10	No
ALLO	ROT	0.300594	4	No
DIA	ROT	0.3005857	13	No
PEP-A	ROT	0.3005262	5	No
AQ	CYTD	0.2988855	9	No
CYTD	ROT	0.2986099	8	No
ROT	TLM	0.2981996	14	No
AQ	MZB	0.2980578	6	No
E-PPMP	TLM	0.2977342	10	No
AQ	CYC	0.2976513	7	No
AM	DNTA	0.2957045	10	No
CRL	DIA	0.2955322	14	No
DHA	T-MIR	0.2954067	14	No
AQ	DNTA	0.2950947	2	No
CUR	PEP-A	0.294559	10	No
ATQ	TLM	0.29434	20	No
FADE	SP	0.294288	5	No
AL	DNTA	0.2932389	13	No
DNTA	PEP-A	0.2929154	4	No
CQ	SP	0.2929044	13	No
CYTD	VER	0.2920838	10	No
ALLO	C-MIR	0.2915537	11	No
CYTD	IB	0.2911957	7	No
TOFA	VER	0.2910021	9	No
CUR	E-64	0.2893664	8	No
TCPA	TTFA	0.28931	17	No
AL	CYTD	0.2888599	8	No
PIP	TLM	0.2885312	19	No
AQ	E-PPMP	0.2882114	8	No
CQ	EHNA	0.2869971	18	No
C-MIR	MZB	0.2855917	13	No
CRL	T-MIR	0.2847398	8	No
ALLO	IB	0.2838076	8	No
CYTD	DAN	0.2836277	13	No
E-PPMP	PIP	0.2836043	4	No
TRI	VER	0.2829752	9	No
MZB	PHEN	0.2828974	18	No
GYLP	TLM	0.2820219	28	No
DHA	TRI	0.281586	15	No
QHS	TOFA	0.2811491	18	No
EHNA	PQ	0.2808003	14	No
E-64	SP	0.280762	6	No
DNTA	VER	0.2803038	13	No
CYTD	QHS	0.2794358	15	No
PEP-A	TOFA	0.279065	8	No
DHA	SP	0.2789434	10	No

AL	TOFA	0.2785924	12	No
AQ	CUR	0.2776777	20	No
C-MIR	IB	0.2765583	15	No
CUR	ROT	0.2761144	11	No
CQ	CYC	0.2742999	8	No
DNTA	QHS	0.2739735	10	No
E-PPMP	MZB	0.2714092	15	No
MZB	TCPA	0.2708672	16	No
ROT	T-PPMP	0.270186	15	No
ALLO	DIA	0.2698615	25	No
PIP	SF	0.2689943	17	No
BZD	TOFA	0.268917	20	No
CUR	TLM	0.2686928	23	No
BZD	TRI	0.268567	12	No
PHEN	SF	0.2676246	31	No
CYTD	MZB	0.2675504	15	No
DAN	TRI	0.2662195	20	No
AS	CUR	0.2653034	17	No
SP	VER	0.264129	10	No
FADE	ROT	0.2631496	20	No
CYTD	MFQ	0.2625895	21	No
ALLO	SDZ	0.2623142	16	No
AQ	TTFA	0.2621077	17	No
BZD	SP	0.2619448	22	No
E-PPMP	VER	0.2606948	17	No
E-PPMP	QHS	0.258294	16	No
DIA	TSA	0.2550148	35	No
CYC	CYTD	0.2547115	24	No
CYC	SDZ	0.2541488	30	No
C-MIR	CYC	0.2526423	17	No
AM	CYC	0.2516486	16	No
ATQ	EHNA	0.251579	36	No
MZB	TRI	0.2508144	24	No
TCPA	VER	0.249753	20	No
MTX	PHEN	0.2494655	35	No
DNTA	TSA	0.2475383	30	No
LUMF	MZB	0.2470091	27	No
PQ	SP	0.2466153	15	No
DNTA	PIP	0.2461664	20	No
PHEN	VER	0.2456258	43	No
BZD	DIA	0.2443923	49	No
MFQ	SF	0.244021	38	No
CRL	ROT	0.2436297	28	No
CYTD	DHA	0.2435306	28	No
CG	PHEN	0.2417127	60	No
EHNA	PIP	0.2390167	39	No
IB	MFQ	0.2370944	40	No
IB	TOFA	0.2361789	34	No

AQ	T-PPMP	0.2357285	40	No
FADE	TCPA	0.2354562	34	No
DIA	VER	0.2351944	46	No
DHA	PHEN	0.2348118	52	No
MZB	TOFA	0.234327	47	No
MFQ	TTFA	0.234284	34	No
ALLO	TOFA	0.2331301	45	No
T-MIR	VER	0.2330185	39	No
DNTA	TTFA	0.232867	36	No
ALLO	AQ	0.2327913	33	No
BZD	LUMF	0.2321861	41	No
E-PPMP	PEP-A	0.2321816	47	No
PIP	T-PPMP	0.2316635	45	No
AM	CYTD	0.2315293	30	No
MZB	PIP	0.2314589	35	No
IB	T-MIR	0.2309169	30	No
DAN	TOFA	0.230705	44	No
DNTA	TLM	0.2275821	54	No
E-64	PIP	0.2259837	51	No
T-PPMP	TCPA	0.2243351	37	No
CYTD	PQ	0.2234455	44	No
DNTA	MFQ	0.2228988	40	No
ROT	TTFA	0.222241	38	No
E-PPMP	MFQ	0.2206683	47	No
DIA	GYLP	0.2194063	120	No
PEP-A	SDZ	0.2190352	42	No
T-PPMP	TRI	0.2188767	55	No
CQ	T-MIR	0.2172261	54	No
AM	PEP-A	0.2160795	55	No
DNTA	FADE	0.2155085	52	No
DNTA	SF	0.2153968	58	No
EHNA	MTX	0.2153461	73	No
AL	SF	0.2149979	56	No
CUR	PIP	0.2143305	48	No
AS	TSA	0.2129012	47	No
AL	CQ	0.2091231	52	No
MFQ	TOFA	0.2074844	64	No
DIA	TCPA	0.2074497	86	No
AM	IB	0.2070461	62	No
ALLO	T-MIR	0.2051491	89	No
SF	T-PPMP	0.2021929	68	No
AL	SDZ	0.2021306	63	No
MFQ	ROT	0.201332	70	No
CQ	SDZ	0.2003122	66	No
SP	T-PPMP	0.1999289	65	No
CYC	ROT	0.1997787	67	No
ALLO	CYTD	0.1993925	66	No
CQ	LUMF	0.199302	74	No

GYLP	T-PPMP	0.1984993	110	No
AL	BZD	0.1981024	76	No
AS	PQ	0.1976027	70	No
E-64	TCPA	0.1963877	74	No
BZD	T-PPMP	0.1959693	95	No
AL	E-64	0.1959259	76	No
CQ	TOFA	0.1955735	91	No
TCPA	TSA	0.1949231	94	No
CUR	DAN	0.1926862	97	No
MZB	ROT	0.1923711	89	No
DIA	FADE	0.1923025	115	No
BZD	PHEN	0.1899221	104	No
CYC	TRI	0.1895336	93	No
ROT	VER	0.1890297	86	No
AM	SDZ	0.1880277	95	No
PHEN	T-PPMP	0.1873926	126	No
AS	CRL	0.1870733	104	No
CYC	TOFA	0.1864499	103	No
AS	TTFA	0.186199	88	No
CQ	E-PPMP	0.1855209	114	No
AM	PQ	0.1853443	89	No
ROT	TSA	0.1817833	106	No
QHS	SDZ	0.1804179	114	No
AM	E-64	0.1786257	112	No
GYLP	TCPA	0.1785939	160	No
AS	FADE	0.1782982	107	No
SP	TLM	0.1781312	139	No
MTX	TCPA	0.1773949	100	No
CQ	CYTD	0.1759108	110	No
CUR	IB	0.1745267	102	No
AS	CYC	0.1726523	130	No
AS	DAN	0.1713557	137	No
AS	E-64	0.1694577	128	No
MZB	SP	0.1655497	141	No
CYC	MFQ	0.1648375	153	No
CYC	DNTA	0.164412	141	No
AM	TSA	0.163599	147	No
DIA	TTFA	0.1633233	186	No
CQ	T-PPMP	0.1628301	158	No
AQ	TLM	0.1606334	195	No
PHEN	PQ	0.1582341	182	No
PHEN	SDZ	0.1579396	209	No
IB	SP	0.1577809	164	No
CQ	ROT	0.156259	186	No
ALLO	AM	0.1545393	177	No
GYLP	TSA	0.1544548	199	No
ALLO	DNTA	0.1541024	160	No
CUR	MFQ	0.1536202	155	No

AL	CYC	0.1531513	157	No
C-MIR	EHNA	0.153075	207	No
ALLO	CUR	0.1528125	175	No
AS	SF	0.1518084	174	No
AL	PEP-A	0.1516495	165	No
BZD	TCPA	0.151008	186	No
AS	DHA	0.150341	179	No
GYLP	QHS	0.1494477	228	No
IB	ROT	0.1484451	183	No
IB	SDZ	0.1471888	209	No
AS	QHS	0.1462778	207	No
ALLO	AS	0.1454841	189	No
C-MIR	DIA	0.145038	250	No
ATQ	DIA	0.1450015	230	No
CYTD	PYR	0.1448865	187	No
GYLP	VER	0.1443123	234	No
CYC	DIA	0.1441266	187	No
AM	FADE	0.1438347	208	No
DAN	ROT	0.1413553	190	No
AM	BZD	0.1404829	232	No
AS	VER	0.1403471	206	No
AL	FADE	0.1397479	208	No
DNTA	IB	0.1391856	187	No
MZB	T-MIR	0.1389792	207	No
E-PPMP	IB	0.1369919	229	No
AS	T-PPMP	0.1354351	244	No
IB	TCPA	0.1350723	224	No
AS	BZD	0.133463	267	No
DIA	TRI	0.1306757	247	No
ALLO	E-PPMP	0.1280488	248	No
DAN	GYLP	0.1263443	302	No
AM	TTFA	0.1258709	283	No
ALLO	TRI	0.1251814	286	No
AM	CRL	0.1241444	278	No
IB	TRI	0.1226859	264	No
EHNA	SDZ	0.1222889	329	No
CUR	CYC	0.1218729	265	No
GYLP	MFQ	0.1214853	338	No
AQ	EHNA	0.1179774	338	No
AL	PQ	0.1176504	278	No
AL	IB	0.1173561	306	No
CG	EHNA	0.1150576	374	No
CYC	SP	0.1134266	288	No
CQ	DNTA	0.1115861	320	No
AL	DAN	0.1105258	333	No
FADE	MFQ	0.1104979	327	No
CG	GYLP	0.1092232	425	No
DAN	E-PPMP	0.1090911	332	No

EHNA	LUMF	0.1090203	355	No
PHEN	QHS	0.1089238	361	No
CYTD	PHEN	0.1076635	351	No
AL	TTFA	0.106935	376	No
CYC	GYLP	0.1054142	390	No
AL	CUR	0.1049018	358	No
DHA	MFQ	0.1038922	367	No
CYTD	EHNA	0.1016893	429	No
EHNA	T-PPMP	0.1009894	415	No
CG	TCPA	0.0979984	409	No
ALLO	GYLP	0.097759	425	No
DIA	PQ	0.0969906	416	No
AL	TLM	0.0966328	433	No
AS	PEP-A	0.0955403	386	No
MFQ	TSA	0.095102	385	No
DIA	QHS	0.0939125	427	No
DHA	GYLP	0.0903228	452	No
CRL	GYLP	0.089461	486	No
GYLP	PIP	0.0866486	498	No
EHNA	TRI	0.0860475	479	No
DHA	TCPA	0.0855086	429	No
DIA	T-MIR	0.0851964	456	No
AS	DIA	0.0833197	505	No
AM	VER	0.0813126	497	No
PEP-A	PHEN	0.0806506	512	No
GYLP	TTFA	0.0803191	537	No
MFQ	T-PPMP	0.0794537	494	No
AM	TLM	0.0787508	516	No
DIA	PIP	0.078124	530	No
CRL	MFQ	0.0748498	494	No
PQ	TCPA	0.0746617	503	No
EHNA	T-MIR	0.0734319	522	No
AS	IB	0.0723017	511	No
E-PPMP	PHEN	0.0710597	549	No
EHNA	PYR	0.0695827	561	No
AM	T-PPMP	0.0686205	562	No
AL	TSA	0.0655958	548	No
PEP-A	TCPA	0.0624435	574	No
SF	TCPA	0.0618116	546	No
AS	TLM	0.0612375	617	No
CQ	DIA	0.0603881	601	No
DIA	SF	0.0563788	637	No
MFQ	VER	0.0561587	633	No
DIA	SP	0.055668	628	No
DAN	SDZ	0.0552108	652	No
AL	CRL	0.0522507	673	No
CG	CQ	0.051708	681	No
GYLP	IB	0.0511657	667	No

DAN	TCPA	0.0504522	651	No
AS	CQ	0.0503084	685	No
ATQ	PYR	0.0498645	689	No
C-MIR	PYR	0.0470867	668	No
CUR	PHEN	0.0443666	711	No
EHNA	SP	0.0435661	744	No
CQ	PHEN	0.0435136	709	No
CG	DIA	0.0435103	759	No
C-MIR	T-PPMP	0.0389907	709	No
GYLP	PEP-A	0.0385243	774	No
AM	CUR	0.0375228	754	No
FADE	GYLP	0.0366829	769	No
PYR	T-MIR	0.0360434	749	No
CG	T-PPMP	0.0357896	783	No
EHNA	GYLP	0.0345312	797	No
AL	VER	0.0330514	785	No
AS	MZB	0.0307703	782	No
E-64	GYLP	0.0271452	816	No
ALLO	SP	0.0259374	819	No
PYR	VER	0.0258809	829	No
DAN	DNTA	0.0223811	847	No
CUR	EHNA	0.0217306	863	No
GYLP	MZB	0.0195518	884	No
C-MIR	PHEN	0.0187793	872	No
CQ	GYLP	0.0168083	894	No
QHS	TCPA	0.0161928	890	No
MFQ	TLM	0.0156883	899	No
PYR	SDZ	0.0150403	903	No
GYLP	LUMF	0.0149692	906	No
GYLP	TRI	0.0141311	905	No
CQ	PYR	0.0128989	919	No
ALLO	AL	0.0127372	913	No
E-PPMP	GYLP	0.0124759	937	No
DIA	EHNA	0.0122126	914	No
GYLP	MTX	0.0117312	924	No
GYLP	TOFA	0.0110483	926	No
PYR	TOFA	0.0106143	932	No
AM	EHNA	0.0095705	938	No
DIA	PEP-A	0.0061974	965	No
CG	TSA	0.003202	971	No
AQ	PYR	0.0031391	981	No
DAN	PHEN	0.0028337	984	No
AM	MZB	0.0015808	992	No
PYR	TSA	0.0013099	988	No
PIP	TCPA	0.0011743	991	No
IB	PYR	0.001084	992	No
PIP	PYR	-0.0014	988	No
CUR	PYR	-0.003038	986	No

GYLP	SP	-0.003062	979	No
E-PPMP	EHNA	-0.004112	976	No
T-MIR	T-PPMP	-0.005477	964	No
CYTD	TCPA	-0.006945	939	No
PHEN	TRI	-0.00721	947	No
ALLO	CG	-0.01146	934	No
AM	GYLP	-0.011814	931	No
DNTA	TCPA	-0.011891	922	No
AS	EHNA	-0.016849	873	No
ATQ	PHEN	-0.017136	877	No
AM	CQ	-0.01893	865	No
CYTD	GYLP	-0.019355	880	No
ALLO	MFQ	-0.020166	844	No
AL	MZB	-0.023837	822	No
CG	DAN	-0.024198	826	No
EHNA	ROT	-0.025703	828	No
E-64	MFQ	-0.026447	807	No
AS	GYLP	-0.027021	830	No
GYLP	PQ	-0.027083	822	No
DAN	PYR	-0.030804	765	No
MZB	PYR	-0.030827	778	No
DNTA	GYLP	-0.031222	801	No
AL	EHNA	-0.031564	804	No
CYC	PYR	-0.03252	769	No
EHNA	MFQ	-0.033198	808	No
CG	IB	-0.036515	775	No
ATQ	GYLP	-0.041028	742	No
AQ	GYLP	-0.041317	757	No
ALLO	MTX	-0.042299	691	No
MFQ	SDZ	-0.042449	728	No
LUMF	PHEN	-0.043126	712	No
DAN	MTX	-0.043282	699	No
MFQ	MZB	-0.045426	670	No
BZD	MFQ	-0.045608	690	No
FADE	PYR	-0.04589	674	No
AL	DIA	-0.046363	711	No
SDZ	TCPA	-0.049509	655	No
PHEN	PYR	-0.049765	665	No
DIA	IB	-0.050381	693	No
CRL	PYR	-0.051149	670	No
CYC	MTX	-0.051886	653	No
GYLP	T-MIR	-0.052345	665	No
ATQ	TCPA	-0.054336	627	No
CG	VER	-0.055167	641	No
ALLO	PYR	-0.05935	594	No
MTX	TSA	-0.063157	577	No
GYLP	SF	-0.063332	593	No
PHEN	PIP	-0.063514	602	No

PHEN	T-MIR	-0.064789	575	No
DNTA	PHEN	-0.068916	568	No
CG	CYC	-0.069566	579	No
CRL	CG	-0.070553	551	No
GYLP	PYR	-0.07109	577	No
DIA	PYR	-0.071125	519	No
ROT	TCPA	-0.07114	534	No
BZD	PYR	-0.07384	513	No
GYLP	ROT	-0.075458	539	No
C-MIR	GYLP	-0.076821	521	No
DNTA	EHNA	-0.078544	499	No
PYR	TCPA	-0.079765	473	No
MFQ	PYR	-0.079973	490	No
IB	PHEN	-0.082461	507	No
CQ	MTX	-0.082488	458	No
EHNA	TOFA	-0.082915	513	No
BZD	GYLP	-0.084266	517	No
LUMF	TCPA	-0.086247	441	No
PEP-A	PYR	-0.08645	445	No
AS	PYR	-0.087105	441	No
PYR	T-PPMP	-0.087252	436	No
CG	MZB	-0.087833	482	No
PHEN	TOFA	-0.09118	468	No
AL	T-PPMP	-0.092944	424	No
E-PPMP	PYR	-0.09379	383	No
CQ	TCPA	-0.095488	377	No
CRL	MTX	-0.109201	324	No
LUMF	PYR	-0.111778	342	No
GYLP	SDZ	-0.112593	374	No
AM	PYR	-0.114544	326	No
CUR	GYLP	-0.124429	311	No
MTX	VER	-0.126424	269	No
MTX	T-PPMP	-0.128423	274	No
CG	CYTD	-0.130491	273	No
MFQ	PHEN	-0.132875	283	No
MTX	PIP	-0.132916	242	No
IB	MTX	-0.135592	231	No
AQ	PHEN	-0.13605	247	No
CG	PIP	-0.139675	248	No
PYR	TTFA	-0.144548	196	No
DIA	MTX	-0.146242	232	No
DIA	LUMF	-0.148448	240	No
AL	GYLP	-0.150809	228	No
AQ	TCPA	-0.151061	184	No
BZD	CG	-0.157968	190	No
AS	TCPA	-0.15952	166	No
PHEN	SP	-0.162527	178	No
CG	PEP-A	-0.162543	187	No

PYR	QHS	-0.163825	144	No
AQ	DIA	-0.164906	170	No
AL	PHEN	-0.17252	151	No
CG	FADE	-0.175246	146	No
C-MIR	TCPA	-0.175339	117	No
MTX	MZB	-0.176537	122	No
CG	TLM	-0.18081	178	No
T-MIR	TCPA	-0.184824	98	No
AM	TCPA	-0.188121	105	No
AQ	MTX	-0.188258	91	No
CG	TOFA	-0.190765	110	No
FADE	MTX	-0.192311	93	No
MTX	TTFA	-0.193838	91	No
DHA	PYR	-0.201485	67	No
CUR	TCPA	-0.206584	72	No
PQ	PYR	-0.207601	62	No
MTX	MFQ	-0.20883	64	No
E-PPMP	TCPA	-0.208853	59	No
MFQ	TCPA	-0.20893	64	No
DNTA	PYR	-0.209185	55	No
ATQ	MTX	-0.210288	62	No
AM	DIA	-0.210822	85	No
AL	PYR	-0.213337	50	No
PYR	SF	-0.215403	48	No
CG	TTFA	-0.216944	63	No
DAN	DIA	-0.219502	61	No
CG	SF	-0.219584	66	No
PYR	TLM	-0.225615	53	No
E-64	PYR	-0.228999	42	No
ATQ	CG	-0.230045	44	No
MTX	TLM	-0.231933	69	No
CG	CUR	-0.232794	64	No
SP	TCPA	-0.236587	40	No
CG	PQ	-0.245847	45	No
CG	E-64	-0.246669	39	No
CG	QHS	-0.273569	21	No
CG	DHA	-0.278317	20	No
MTX	SF	-0.279113	9	No
BZD	MTX	-0.279268	11	No
DIA	MFQ	-0.288377	17	No
AM	MTX	-0.296738	10	No
AM	PHEN	-0.299631	9	No
MTX	PEP-A	-0.301718	9	No
CG	MFQ	-0.305753	14	No
CUR	MTX	-0.3074	6	No
MTX	QHS	-0.307608	4	No
CYTD	MTX	-0.311252	4	No
AQ	CG	-0.313906	6	No

AS	PHEN	-0.315244	11	No
AL	TCPA	-0.317493	5	No
PYR	TRI	-0.321964	2	No
CG	E-PPMP	-0.325419	4	No
AS	MTX	-0.331135	2	No
PYR	ROT	-0.334086	2	No
LUMF	MTX	-0.335144	2	No
E-64	MTX	-0.338972	5	No
C-MIR	CG	-0.341183	6	No
AS	CG	-0.345683	2	No
MTX	PQ	-0.349541	0	No
DHA	MTX	-0.370991	0	No
CG	LUMF	-0.373162	3	No
C-MIR	MTX	-0.382827	1	No
MTX	TOFA	-0.385662	0	No
CG	T-MIR	-0.387549	0	No
AM	CG	-0.406848	1	No
CG	DNTA	-0.407833	0	No
MTX	TRI	-0.412493	0	No
AL	MTX	-0.433124	0	No
CG	TRI	-0.444277	0	No
AL	CG	-0.450121	0	No
CG	ROT	-0.478111	0	No
MTX	T-MIR	-0.482207	0	No
E-PPMP	MTX	-0.508788	0	No
DNTA	MTX	-0.554702	0	No
MTX	ROT	-0.572487	0	No

TABLE S6:

Gene ID/Mutant	ATQ	ROT	LUMF	AL
PF3D7_1013500	0.02833	1.329	0.11	0.79
PF3D7_1126100	0.05725	0.047	0.15	0.46
PF3D7_1327100	0.11932	0.175	0.05	0.69
PF3D7_1226900	0.18584	0.442	0.72	0.9
PF3D7_1441800	0.27144	0.21	0.44	1.03
PF3D7_1107900	0.28667	0.018	0.23	0.5
PF3D7_0727100	0.28756	0.114	0.63	1.92
PF3D7_1001600	0.3791	0.51	0.63	1.2
PF3D7_0902200	0.45218	0.063	0.49	0.73
PF3D7_1019400	0.48074	2.64	0.39	1.91
PF3D7_0812500	0.51611	9.8	3.01	1.09
PF3D7_1143500	0.51954	0.08	0.22	1.32
PF3D7_1216100	0.71458	0.68	0.79	0.45
PF3D7_1433400	0.73	0.957	1.14	2.44
PF3D7_1219300	0.74293	3.7	0.82	1.34
PF3D7_1207800	0.74775	1.08	0.88	2.38
PF3D7_0402000	0.76333	3.266	0.94	2.43
PF3D7_1133700	0.85212	13.75	4.84	0.92
PF3D7_1475600	0.92029	0.653	34.6	0.53
PF3D7_1103100	0.94474	1.6	4	1.38
PF3D7_0322000	1.04	6.764	1.38	0.91
PF3D7_0916700	1.13833	1.267	0.78	1.79
PF3D7_1343000	1.32566	0.113	1.35	0.82
PF3D7_1136000	1.52049	0.62	9.3	1.3
PF3D7_1001900	1.62667	1.955	2.98	2.25
PF3D7_0511500	1.67194	1.28	0.11	5
PF3D7_0103300	1.80833	191.8	3.34	3.65
PF3D7_1330500	1.92931	0.271	2.2	0.79
PF3D7_0702300	2.10833	6.139	2.6	1.68
PF3D7_0203600	2.40405	33.82	20.7	2.5
PF3D7_1343900	3.03323	1.217	0.76	0.8
PF3D7_0211100	3.08333	0.301	1.25	0.81
PF3D7_1432400	3.29082	1.488	3.16	0.7

PF3D7_0404600	3.51087	6.937	0.67	1.13
PF3D7_0203700	3.63561	0.057	1.79	0.84
PF3D7_0521900	3.76835	12.8	4.6	1.57
PF3D7_0931000	4.10741	6.22	20.6	1.6
PF3D7_0614500	4.11894	0.359	2.16	1.19
PF3D7_1138900	4.74844	10.99	16	1.66
PF3D7_0619900	4.90408	3.7	0.44	1.14
PF3D7_1444100	5.20928	11.51	21.3	2.1
PF3D7_0601700	5.74296	0.102	2.05	1.28
PF3D7_1035800	7.02833	24.88	12.5	1.5
PF3D7_1302000	9.18243	0.155	2.4	0.6
PF3D7_1311900	13.3487	8.8	34.6	1.82

Legends to Table:

Table S6: A rank comparison of fold change from wild type GI_{50} values in presence of mitochondrial inhibitors (ATQ, ROT) with non mitochondrial inhibitors AL. A Spearman correlation of the chemo-genetic values (RPR) between the drug groups ATQ-LUMF shows positive correlation (0.53) whereas AL-LUMF was negative (0.1) indicating LUMF might also act on mitochondrial targets.

Table S7 : P. falciparum gene clusters of drug responses. (The clusters are defined with Ward's minimum variance criterion, the number of clusters are determined by using maximum Cubic Clustering Criteria while increasing the cluster number)

Cluster	PB ID	PlasmoDB ID	Genomic Location	Annotation	GO Annotations
**cluster_DNR**					
	PB-11	PF3D7_0416500	Pf3D7_04_v3: 723,073 - 724,242 (-)	repressor of RNA polymerase III transcription MAF1, putative	null
	PB-12	PF3D7_1018400	Pf3D7_10_v3: 734,722 - 735,738 (+)	conserved Plasmodium protein, unknown function	null
	PB-13	PF3D7_1360200	Pf3D7_13_v3: 2,406,838 - 2,408,385 (-)	conserved protein, unknown function	null
	PB-15	PF3D7_0611800	Pf3D7_06_v3: 490,858 - 501,140 (-)	conserved Plasmodium protein, unknown function	null
	PB-17	PF3D7_0521900	Pf3D7_05_v3: 888,657 - 895,144 (+)	conserved Plasmodium protein, unknown function	ATP binding, actin binding, calmodulin binding, motor activity
	PB-18	PF3D7_1219300	Pf3D7_12_v3: 766,654 - 774,197 (-)	erythrocyte membrane protein 1, PfEMP1 (VAR)	cell adhesion molecule binding, host cell surface receptor binding, receptor activity
	PB-3	PF3D7_0615900	Pf3D7_06_v3: 662,435 - 667,855 (-)	conserved Plasmodium protein, unknown function	catalytic activity
**cluster_TRI**					
	PB-4	PF3D7_1122900	Pf3D7_11_v3: 878,969 - 895,273 (+)	dynein heavy chain, putative	ATP binding, ATPase activity, microtubule motor activity, motor activity
	PB-5	PF3D7_1141900	Pf3D7_11_v3: 1,676,255 - 1,677,769 (-)	inner membrane complex protein 1b, putative (IMC1b)	structural constituent of cytoskeleton
**cluster_CYTD**					
	PB-19	PF3D7_1133700	Pf3D7_11_v3: 1,302,173 - 1,305,987 (+)	conserved Plasmodium protein, unknown function	null
	PB-21	PF3D7_1136000	Pf3D7_11_v3: 1,403,906 - 1,414,512 (+)	conserved Plasmodium protein, unknown function	actin binding, calmodulin binding, motor activity, nucleotide binding
	PB-39	PF3D7_1103100	Pf3D7_11_v3: 140,048 - 140,801 (-)	60S acidic ribosomal protein P1, putative	structural constituent of ribosome
	PB-42	PF3D7_1216100	Pf3D7_12_v3: 646,979 - 647,860 (+)	conserved Plasmodium membrane protein, unknown function	null
	PB-45	PF3D7_1226900	Pf3D7_12_v3: 1,088,536 - 1,090,023 (-)	conserved Plasmodium protein, unknown function	ATP binding, lipoate-protein ligase activity, transferase activity
	PB-64	PF3D7_1107900	Pf3D7_11_v3: 343,913 - 349,351 (-)	mechanosensitive ion channel protein	null
	PB-76	PF3D7_1433400	Pf3D7_14_v3: 1,312,304 - 1,329,577 (-)	conserved Plasmodium membrane protein, unknown function	protein binding, zinc ion binding
	PB-87	PF3D7_0812500	Pf3D7_08_v3: 626,069 - 628,945 (-)	RNA binding protein, putative	nucleic acid binding
**cluster_CUR**					
	PB-71	PF3D7_1001900	Pf3D7_10_v3: 99,379 - 100,361 (-)	Plasmodium exported protein (hyp16), unknown function (PfJ23)	null
	PB-77	PF3D7_0322000	Pf3D7_03_v3: 928,644 - 929,159 (-)	peptidyl-prolyl cis-trans isomerase (CYP19A)	cyclosporin A binding, peptidyl-prolyl cis-trans isomerase activity
	PB-78	PF3D7_0623900	Pf3D7_06_v3: 977,183 - 978,049 (+)	ribonuclease H2 subunit A, putative	RNA binding, ribonuclease H activity
**cluster_ART_FADE_PPMI**					
	PB-35	PF3D7_1001600	Pf3D7_10_v3: 86,538 - 89,009 (-)	alpha/beta hydrolase, putative	null
	PB-54	PF3D7_0902200	Pf3D7_09_v3: 98,175 - 100,339 (-)	serine/threonine protein kinase, FIKK family (FIKK9.3)	ATP binding, protein serine/threonine kinase activity
	PB-50	PF3D7_1138700	Pf3D7_11_v3: 1,525,654 - 1,531,744 (-)	conserved Plasmodium protein, unknown function	ATP binding, actin binding, calmodulin binding, motor activity
	PB-55	PF3D7_0727100	Pf3D7_07_v3: 1,152,155 - 1,155,355 (-)	conserved Plasmodium protein, unknown function	extracellular matrix structural constituent
	PB-52	PF3D7_1136600	Pf3D7_11_v3: 1,441,503 - 1,445,471 (+)	conserved Plasmodium protein, unknown function	ATP binding, actin binding, motor activity
	PB-57	PF3D7_1126100	Pf3D7_11_v3: 1,018,366 - 1,022,495 (-)	autophagy-related protein 7, putative (ATG7)	catalytic activity
	PB-58	PF3D7_1343700	Pf3D7_13_v3: 1,724,817 - 1,726,997 (-)	kelch protein, putative	protein binding, voltage-gated potassium channel activity
**cluster_ROT**					
	PB-60	PF3D7_1330500	Pf3D7_13_v3: 1,284,988 - 1,287,165 (+)	conserved Plasmodium protein, unknown function	null
	PB-65	PF3D7_0606000	Pf3D7_06_v3: 250,612 - 253,803 (-)	conserved Plasmodium protein, unknown function	null
	PB-66	PF3D7_0614500	Pf3D7_06_v3: 606,142 - 607,259 (-)	60S ribosomal protein L19, putative	structural constituent of ribosome
	PB-68	PF3D7_0601700	Pf3D7_06_v3: 70,948 - 71,935 (+)	Plasmodium exported protein (PHISTa), unknown function, pseudog	nucleotide binding
	PB-62	PF3D7_1302000	Pf3D7_13_v3: 112,792 - 113,815 (-)	Plasmodium exported protein, unknown function	null
**cluster_EHNA_TCPA**					
	PB-22	PF3D7_0931000	Pf3D7_09_v3: 1,248,866 - 1,251,601 (-)	elongation factor Tu, putative	GTP binding, GTPase activity
	PB-23	PF3D7_0830900	Pf3D7_08_v3: 1,317,600 - 1,318,341 (-)	Plasmodium exported protein, unknown function	null
	PB-28	PF3D7_1138900	Pf3D7_11_v3: 1,540,804 - 1,544,163 (+)	unspecified product	null

TABLE S8. List of chemical inhibitors used to create chemogenomic profiles.

ALLO	allopurinol	LUMF	lumifantrine
AQ	amodiaquin	MTX	methotrexate
AL	artelinic acid	MFQ	mefloquine
AM	artemether	MZB	mizoribine
AS	artesunate	T-MIR	*trans*-mirincamycin
ATQ	atovaquone	PQ	primaquine
BZD	benzimidazole	PEP-A	pepstatin A
CG	cycloguanil	PHEN	phenethroline
C-MIR	*cis*-mirincamycin	PIP	piperaquine
CRL	cerulinin	E-PPMP	*erythro*-PPMP
CQ	chloroquine	PYR	pyrimethamine
CUR	curcumin	T-PPMP	*threo*-PPMP
CYTD	cytochalasin D	QHS	artemisnin (qinghaosu)
CYC	D-cycloserine	ROT	rotenone
DAN	diazooxonorleucine	SF	sinefungin
DHA	dihydroartemisnin	SDZ	sulfadiazine
DNR	daunorubicin	SP	sulfadoxine
DNTA	dinitroaniline	TCPA	fenoxaprop
E-64	*trans*-epoxysuccinyl-L-leucylamido(4-guanidino)butane	TOFA	5-(tetradecyloxy)-2-furoic acid
		TSA	triclostatin A
EHNA	*erythro*-9-(2-hydroxy-3-noyl)-adenine hydrochloride	TTFA	thenoyltrifluoroacetone
		TLM	thiolactomycin
FADE	2-fluoroadenosine	TRI	triclosan
IB	ibandronate	VER	verapamil

Table S9: Genes directly connected to K13-propeller in the co-expression network that contains SNPs associated with parasite clearance half-life[1].

**Chr.**	**Position**	***P* value**	**FDR corrected *P* value**	**Gene ID**	**Gene Description**	**GO Biological Process**
5	261651	3.73E-05	0.01035	PF3D7_0506300	conserved Plasmodium protein, unknown function	transferase activity
5	852479	7.56E-06	0.00295	PF3D7_0520800	conserved Plasmodium protein, unknown function	nucleotide binding
5	855646	1.70E-06	0.00084	PF3D7_0520800	conserved Plasmodium protein, unknown function	nucleotide binding
11	1765914	4.26E-05	0.01148	PF3D7_1144700	apicoplast import protein Tic20, putative (TIC20)	null
13	1913206	4.17E-07	0.00025	PF3D7_1347900	conserved Plasmodium protein, unknown function	null
13	1916603	1.41E-05	0.00507	PF3D7_1347900	conserved Plasmodium protein, unknown function	null
13	1942524	8.11E-05	0.01790	PF3D7_1348400	conserved Plasmodium membrane protein, unknown function	null
13	2036738	2.84E-05	0.00839	PF3D7_1351000	phosphatidylinositol transfer protein, putative	phospholipid binding, phospholipid transporter activity, protein binding
13	2045445	0.00023	0.03854	PF3D7_1351200	conserved Plasmodium protein, unknown function	beta-galactosidase activity, carbohydrate binding, cation binding
13	2373848	2.18E-05	0.00677	PF3D7_1359600	conserved Plasmodium protein, unknown function	null

SNPs in ART Sensitivity Cluster

Chr.	Position	*P* value	FDR corrected *P* value	Gene ID
7	1152349	0.624352898	0.999168074	PF3D7_0727100
7	1153700	0.052261485	0.640491591	PF3D7_0727100
7	1154285	0.67345175	0.999168074	PF3D7_0727100
7	1154938	0.273834272	0.948151217	PF3D7_0727100
9	100036	0.501048984	0.996524854	PF3D7_0902200
10	86696	0.545224521	0.999168074	PF3D7_1001600
10	87116	0.51030273	0.996524854	PF3D7_1001600
10	87127	0.173511036	0.88878064	PF3D7_1001600
10	87154	0.712222974	0.999168074	PF3D7_1001600
10	87272	0.014655856	0.394309242	PF3D7_1001600
10	87315	0.376018174	0.979304191	PF3D7_1001600
10	88346	0.776104441	0.999168074	PF3D7_1001600
10	88477	0.742600739	0.999168074	PF3D7_1001600
11	1018820	0.084794757	0.722945341	PF3D7_1126100
11	1018899	0.57273469	0.999168074	PF3D7_1126100
11	1018965	0.20484751	0.915418556	PF3D7_1126100
11	1019067	0.652071716	0.999168074	PF3D7_1126100
11	1019333	0.680729181	0.999168074	PF3D7_1126100
11	1019514	0.64560366	0.999168074	PF3D7_1126100
11	1019560	0.013139713	0.374410298	PF3D7_1126100
11	1020026	0.546917491	0.999168074	PF3D7_1126100
11	1020061	0.901987242	0.999168074	PF3D7_1126100
11	1020134	0.76246311	0.999168074	PF3D7_1126100
11	1020397	0.952300571	0.999168074	PF3D7_1126100
11	1020595	0.202865615	0.912304383	PF3D7_1126100
11	1020630	0.875957182	0.999168074	PF3D7_1126100
11	1020787	0.088593034	0.731831185	PF3D7_1126100

11	1020986	0.033312856	0.547897799	PF3D7_1126100
11	1021415	0.024414917	0.488351648	PF3D7_1126100
11	1022005	0.412305606	0.987728487	PF3D7_1126100
11	1022269	0.306718445	0.957937512	PF3D7_1126100
11	1442727	0.755881336	0.999168074	PF3D7_1136600
11	1443302	0.897765595	0.999168074	PF3D7_1136600
11	1444265	0.81416611	0.999168074	PF3D7_1136600
11	1444859	0.798597482	0.999168074	PF3D7_1136600
11	1527675	0.56373269	0.999168074	PF3D7_1138700
11	1528312	0.436448097	0.989556	PF3D7_1138700
11	1528678	0.570476365	0.999168074	PF3D7_1138700
11	1529693	0.543302386	0.999168074	PF3D7_1138700
11	1529778	0.673573926	0.999168074	PF3D7_1138700
11	1529784	0.993416376	0.999651938	PF3D7_1138700
11	1529801	0.996731805	0.999950846	PF3D7_1138700
11	1530261	0.26260381	0.946454468	PF3D7_1138700
13	1724974	0.129794441	0.822232908	PF3D7_1343700
13	1725259	4.23533E-26	7.77E-22	PF3D7_1343700
13	1725370	0.246264559	0.937817021	PF3D7_1343700
13	1725382	0.375687046	0.979021229	PF3D7_1343700
13	1725385	0.287140984	0.954462465	PF3D7_1343700
13	1725521	0.005562035	0.248878488	PF3D7_1343700
13	1726244	0.059778538	0.668744365	PF3D7_1343700
13	1726432	0.33712619	0.967575285	PF3D7_1343700

1 Miotto, O. *et al.* Multiple populations of artemisinin-resistant Plasmodium falciparum in Cambodia. *Nature genetics* **45**, 648-655, doi:10.1038/ng.2624 (2013).
